# Reorganization of Innate Immune Cell Lipid Profiles by Bioinspired Meroterpenoids to Limit Inflammation

**DOI:** 10.1002/advs.76861

**Published:** 2026-08-18

**Authors:** Lorenz Waltl, David Holubek, Klaus Speck, Raphael Wildermuth, Franz‐Lucas Haut, Stephan Permann, Immanuel Plangger, Christian Steinborn, Zhigang Rao, Danilo D'Avino, Ida Cerqua, Julia Stadler, Fiorentina Roviezzo, Peter Schlenke, Anita Siller, Harald Schennach, Solveigh C. Koeberle, Eva‐Maria Pferschy‐Wenzig, Antonietta Rossi, Thomas Magauer, Andreas Koeberle

**Affiliations:** ^1^ Michael Popp Institute and Center for Molecular Biosciences (CMBI) University of Innsbruck Innsbruck Austria; ^2^ Institute of Human Genetics Medical University of Innsbruck Innsbruck Austria; ^3^ Institute of Pharmaceutical Sciences and Excellence Field BioHealth, NAWI Graz University of Graz. Graz Austria; ^4^ Department of Organic Chemistry and Center for Molecular Biosciences (CMBI) University of Innsbruck Innsbruck Austria; ^5^ Department of Pharmacy School of Medicine and Surgery University of Naples Federico II Naples Italy; ^6^ Clinical Department of Blood Group Serology and Transfusion Medicine Medical University of Graz Graz Austria; ^7^ Central Institute for Blood Transfusion and Immunology University Hospital Innsbruck, Tirol Kliniken GmbH Innsbruck Austria

**Keywords:** inflammation, lipid mediators, lipidomics, lipoxygenases, molecular mechanisms, natural products, pharmacology

## Abstract

Lipidomics‐guided screening of unexplored natural product chemical space provides access to small molecules that globally reprogram cellular lipid profiles. Here, we show that the meroterpenoid cyclosmenospongine from *Spongia sp*. reshapes immune cell lipidomes, shifting them from pro‐inflammatory toward anti‐inflammatory and pro‐resolving mediators. Structural variation yielded derivatives that context‐dependently inhibit leukotriene biosynthesis to varying extents while upregulating pro‐resolving lipid mediators, epoxyeicosatrienoic acids, endocannabinoids, sphingosine‐1‐phosphate, and others in resting and activated innate immune cells in vitro, as well as in self‐resolving murine peritonitis and fibrosis in vivo. Mechanistically, meroterpenoids target 5‐lipoxygenase or 5‐lipoxygenase‐activating protein, promote 15‐lipoxygenase‐1 translocation to particulate sites, and inhibit monoacylglycerol lipase. They also redirect arachidonic acid from neutral lipids to specific phospholipids while increasing free arachidonic acid levels. Furthermore, meroterpenoids reprogram immune cell lipid metabolism by reducing neutral lipid, triacylglycerol, and cholesteryl ester levels, a shift that correlates with a lower capacity for leukotriene biosynthesis and is phenocopied by inhibiting sterol‐O‐acyltransferase, which catalyzes cholesterol esterification for storage, and diacylglycerol acyltransferase‐1/2, which catalyze the final step in triacylglycerol biosynthesis. In conclusion, specific meroterpenoids exert anti‐inflammatory effects by rewiring lipid mediator biosynthesis through structure‐controlled switches in lipid mediator classes, and an unexpected link between lipogenesis and inflammation.

## Graphical abstract

Resolution pharmacology is an emerging strategy to tackle inflammatory pathologies. Bioinspired meroterpenoids induce a lipid mediator class switch toward inflammation resolution in vitro and in vivo by targeting key nodes in lipid mediator biosynthesis and neutral lipid dynamics. Network analysis suggests that shifts in neutral lipid levels control leukotriene formation, a mechanism validated by inhibiting neutral‐lipid biosynthetic enzymes.

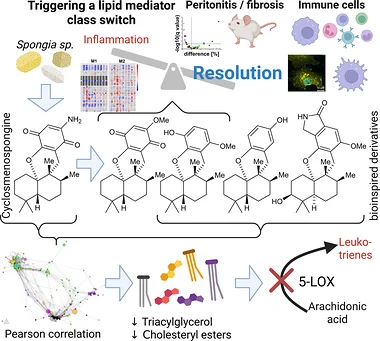

## 1 Introduction

Inflammation is a tightly regulated physiological response to tissue damage or infection that aims to restore homeostasis [1]. Failure to resolve the inflammatory state properly can lead to low‐grade, persistent inflammation, which contributes to a wide range of chronic disorders, including metabolic, autoimmune, neurodegenerative, cardiovascular, and malignant diseases [2, 3, 4, 5]. The initiation, propagation, and termination of inflammation are orchestrated by a multitude of mediators, including cytokines and lipids. The latter constitute a highly diverse class of biomolecules comprising thousands of distinct species [6, 7], among them lipid mediators with pro‐inflammatory, anti‐inflammatory, pro‐resolving, or immunomodulatory activities that act as ligands for membrane‐bound or intracellular receptors [2, 8, 9, 10, 11, 12, 13, 14, 15, 16, 17, 18]. In addition, links between cellular metabolism, immune function, and persistent inflammation [19, 20, 21, 22, 23, 24] are increasingly recognized, blurring the boundaries between structural lipids and signaling molecules, as exemplified by the recent discovery of the lipokine‐like, stress‐protective phosphatidylinositol (PI) 1,2‐dioleoyl‐*sn*‐glycero‐3‐phosphoinositol [25].

Immune and non‐immune cells generate lipid mediators from polyunsaturated fatty acids (PUFAs), which are predominantly released from membrane phospholipids by members of the phospholipase A_2_ (PLA_2_) superfamily [26]. Free PUFAs are subsequently converted into lipid mediators with homeostatic, immunomodulatory, pro‐inflammatory, anti‐inflammatory, or pro‐resolving activities by the coordinated action of di‐ and monooxygenases and additional biosynthetic enzymes [2, 3, 12, 13, 14, 16]. While some lipid mediators can be largely assigned to a specific functional group—such as the pro‐inflammatory leukotrienes (LTs)—others, including prostanoids, exert different biological effects depending on concentration, kinetics, and the (patho)physiological context.

Cyclooxygenase (PTGS, COX) isoenzymes are the primary targets of non‐steroidal anti‐inflammatory drugs (NSAIDs) [16]. These enzymes convert the ω6‐fatty acid arachidonic acid (AA/20:4) into prostaglandin H_2_ (PGH_2_)_,_ the common precursor of prostaglandin E_2_ (PGE_2_) and other prostanoids. PGE_2_ promotes inflammation, fever, and pain, yet it also exhibits homeostatic and anti‐inflammatory functions and can initiate the transition toward resolution of inflammation, depending on its (spatial) concentration and kinetics [16, 27, 28].

5‐Lipoxygenase (ALOX5, 5‐LOX) translocates to the perinuclear membrane, where it accepts AA/20:4 from 5‐lipoxygenase‐activating protein (ALOX5AP, FLAP) and initiates leukotriene (LT) biosynthesis. These lipid mediators attract and activate immune cells, increase vascular permeability, and induce bronchial constriction [3, 14, 16, 29]. In addition, ALOX5 contributes to the formation of specialized pro‐resolving lipid mediators (SPMs), such as lipoxins (LXs), through (transcellular) cooperation with 12‐lipoxygenase (ALOX12, ALOX12B) or 15‐lipoxygenase (ALOX15, ALOX15B) isoenzymes [30, 31]. Notably, phosphorylation of ALOX5 at serine 663 may alter its regiospecificity toward 15‐lipoxygenation [32].

ALOX15, the rate‐limiting and key regulatory enzyme in SPM biosynthesis [33], undergoes a Ca^2+^‐dependent translocation from the cytosol to different cellular membranes [34], including the cytosol‐facing surface of poorly defined particulate compartments [35], observed, among others, in activated M2‐like macrophages [36]. This membrane association stimulates its PUFA‐dioxygenation activity [37]. ALOX15 accepts ω3‐fatty acids and, together with lipoxygenases of different regiospecificity or (partially unknown) oxidoreductases, produces SPMs such as LXs, protectins (PDs), resolvins (Rvs), and maresins (MaRs) [2, 3, 12, 13, 33, 36], which have been proposed to actively promote the resolution of inflammation, possibly through G‐protein‐coupled receptors [2, 38]. SPMs suppress leukocyte infiltration and activation, partly by interfering with nuclear factor (NF)‐κB signaling, which drives the expression of pro‐inflammatory cytokines and chemokines [38, 39] and seem to promote phagocytosis, bacterial clearance, efferocytosis, and tissue repair [2, 33]. Whether SPMs reach biologically effective concentrations in vivo remains a matter of debate, as do the identity of their receptors, the exact biosynthetic routes involved, and the quality of some earlier analytical methods used for their quantification [40, 41, 42, 43, 44]. Interpreting the biological impact of ALOX15 activity is further complicated by the context‐dependent behavior of its products, including 15‐HETE, 13‐hydroxyoctadecadienoic acid (13‐HODE), and 5‐oxo‐15‐HETE, which may act as either anti‐inflammatory/pro‐resolving or pro‐inflammatory mediators [45, 46].

The diversity of lipid mediators is further expanded by the CYP2C and CYP2J families of cytochrome P_450_ monooxygenases (CYP_450_), which primarily catalyze the epoxidation of AA/20:4 and other PUFAs [47, 48]. These epoxy‐fatty acids, among other functions, interfere with immune cell activation, cytokine release, and PTGS2 expression [48]. PUFAs can also be conjugated to form intracellular and extracellular signaling molecules, such as endocannabinoids, which, together with other (lipid) factors like sphingosine‐1‐phosphate (S1P), orchestrate immune cell activation, lymphocyte proliferation, chemotaxis, and cytokine production [49, 50, 51], regulate nociception [51, 52], and are closely linked to cellular metabolism and metabolic diseases [11, 53]. Some of these lipid metabolites, including endocannabinoids, can be further converted by PTGS, ALOX, and CYP_450_ isoenzymes into oxygenated species with potential functional relevance [54].

Current anti‐inflammatory therapies largely rely on glucocorticoids and NSAIDs. Whereas glucocorticoids non‐selectively suppress cytokine and lipid mediator production, NSAIDs inhibit PTGS isoenzymes and are associated with multiple, sometimes severe, adverse effects including ulceration, bleeding, and cardiotoxicity. These side effects are attributed to impaired biosynthesis of homeostatic prostanoids and, potentially, to the redirection of AA/20:4 toward LT formation [16, 55]. Emerging anti‐inflammatory strategies aim to selectively target disease‐related mediators [56, 57, 58] and to promote the resolution of inflammation [4, 33], a concept of particular promise for chronic inflammatory diseases. To date, only a few compounds have been identified that substantially enhance the biosynthesis of SPMs and/or their isomers (SPM/iso) in activated immune cells (up to 10‐fold for MaR1/iso by 8‐methylsocotrin‐4'‐ol) [59, 60, 61, 62, 63, 64, 65, 66, 67, 68, 69, 70, 71, 72, 73, 74]. Even fewer molecules have been shown to induce SPM/iso formation in non‐activated cells (up to 70‐fold for RvD5/iso by cannabidiol) [59, 60, 61, 72, 74, 75, 76]. Glucocorticoids form an exception, as they can promote SPM biosynthesis by upregulating ALOX15B in immune cell types that otherwise produce little to no SPMs [65]. Some of these small molecules also synergize with supplemented ω3‐fatty acids [74]. Overall, the rational adjustment of beneficial lipid mediator profiles remains at an early stage but is expected to advance with the development of dual and multi‐target modulators of lipid mediator metabolism [28, 56, 57, 58, 77].

In this study, we employed lipidome‐wide screening to identify unexplored natural products within an underrepresented chemical space and discovered that the meroterpenoid cyclosmenospongine (**1**; Figure 1) shifts the lipid profile of innate immune cells toward inflammation suppression and resolution. Compound **1**, a *trans*‐decalin meroterpenoid, was first isolated in 2002 from the Australian sponge *Spongia sp*. (order Dictyoceratida, family Spongiidae). Subsequent studies reported its efficacy against methicillin‐resistant *Staphylococcus aureus* strains [78] and its modest inhibition of ascites cancer cell proliferation [78, 79]. Through modular total synthesis, we generated structural derivatives of **1** [78], capable of reprogramming the lipid mediator profile of innate immune cells. These include the synthetic compounds **2**–**5** and **7**–**9**, as well as the natural product (+)‐stachyflin (**6**), previously isolated from *Stachybotrys sp*. RF7260 [80]. These molecules are effective in a murine model of self‐resolving inflammation and redirect lipid mediator formation in a disease model in which an initial inflammation insult triggers pulmonary fibrosis. Depending on innate immune cell type and stimulus, the derivatives context‐dependently inhibit LT biosynthesis to varying degrees, interfere with cytokine expression, and induce SPM/iso formation in activated M2‐like macrophages (up to 3.3‐fold for MaR2/iso) and resting M2‐like macrophages (up to 190‐fold for RvD5/iso). They also upregulate anti‐inflammatory epoxidated fatty acids, such as epoxyeicosatrienoic acids (EETs), and immunomodulatory lipid mediators including PGE_2_, endocannabinoids, and S1P. Mechanistically, the derivatives inhibit ALOX5 or antagonize ALOX5AP, mobilize PUFAs from distinct lipid pools, induce subcellular relocalization of ALOX15, and inhibit monoacylglycerol lipase, an endocannabinoid‐degrading enzyme [11]. They further reprogram cellular metabolism by depleting neutral lipids, thereby supporting the previously proposed link between lipid metabolism and pro‐inflammatory lipid mediator biosynthesis in human innate immune cells. Notably, even minor structural modifications selectively influence the preferential formation of specific SPM/iso subclasses, underscoring the directional manipulability of lipid mediator profiles.

**FIGURE 1:**
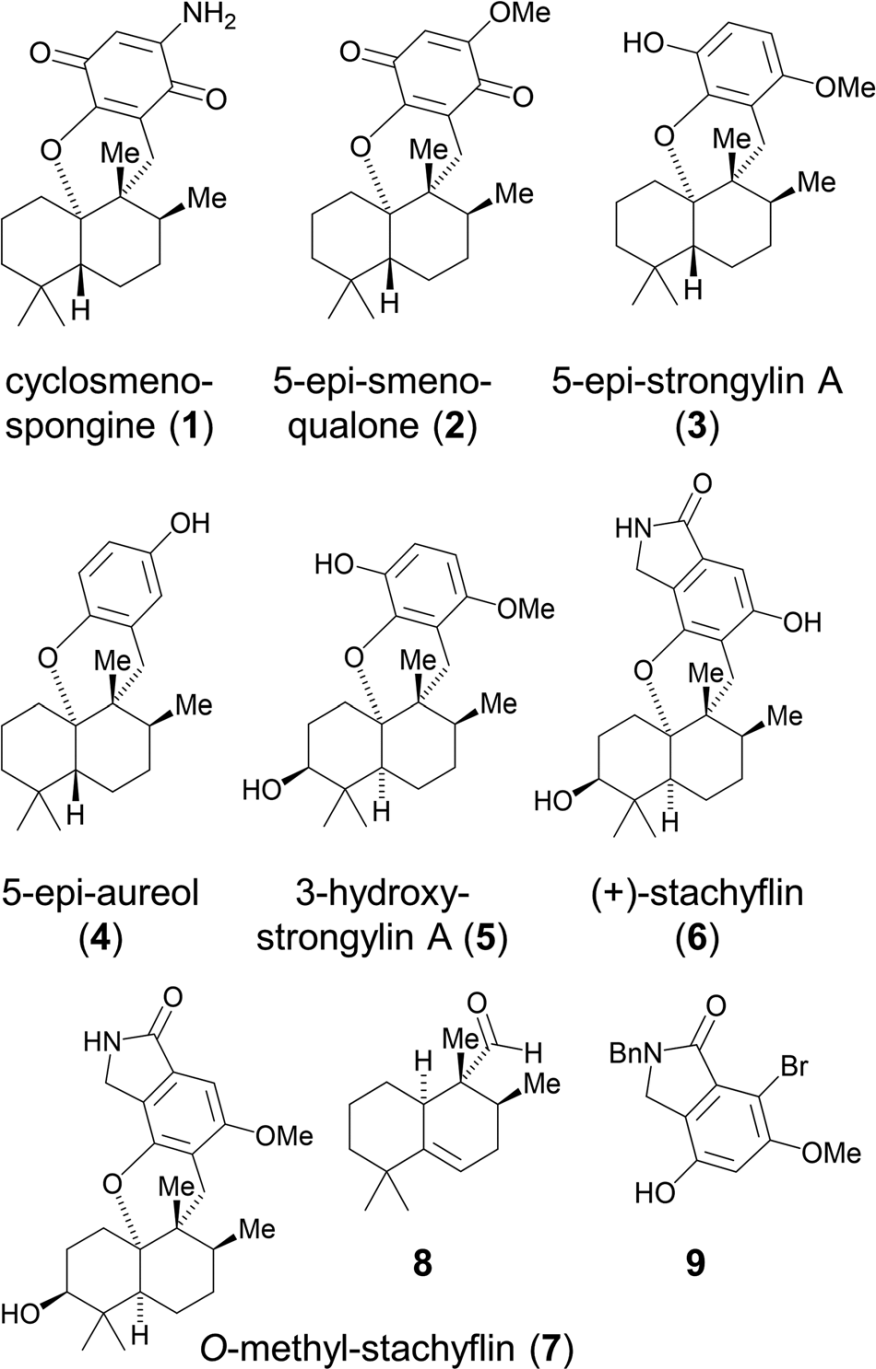
Chemical structures of the natural meroterpenoids 1 and 6, and their structural derivatives 2–5 and 7–9.

## 2 Results

### 2.1 Compound 1 Induces a Lipid Mediator Class Switch From LTs to SPM/Iso

To identify small molecules that suppress pro‐inflammatory while promoting pro‐resolving lipid mediator formation, we first screened natural products in A23187‐activated human peripheral blood mononuclear cells (PBMCs), which consist predominantly of monocytes and also contain dendritic cells, lymphocytes, and natural killer cells [81]. None of the compounds tested showed cytotoxicity within 48 h at concentration up to 10 µM, as assessed by mitochondrial dehydrogenase activity, membrane integrity, and viable cell counts (Figure S1A–D). The hit compound **1** (10 µM; Figure 1) efficiently suppressed the formation of ALOX5 products (LTB_4_, LTB_4_‐isomers, 20‐hydroxy‐LTB_4_ [20‐OH‐LTB_4_], 5‐hydroxyeicosatetraenoic acid [5‐HETE], 5‐hydroxyeicosapentaenoic acid [5‐HEPE], and 5,6‐dihydroxy‐HETE [5,6‐diHETE]) while increasing the production of ALOX12/15 products (Figure 2A and Figure S1E), including precursors of SPMs such as 15‐HETE and 14‐hydroxydocosahexaenoic acid (14‐HDHA) (Figure 2B and Figure S1F; Data Table S1). To evaluate the total output of ALOX12 and ALOX15, we combined their monohydroxylated lipid metabolites, which arise in situ via reduction of the initially formed hydroperoxides, and grouped them by their site of oxygenation. The “mono‐A15‐OH” class (15‐HETE, 15‐HEPE, 17‐HDPA, 17‐HDHA) contains metabolites predominantly generated by ALOX15 but not by ALOX12. In contrast, the “mono‐A12‐OH” class (12‐HETE, 12‐HEPE, 14‐HDPA, 14‐HDHA) comprises metabolites indicative of ALOX12 activity, regardless of whether they were formed by ALOX12 or ALOX15 under the specific experimental conditions.

**FIGURE 2:**
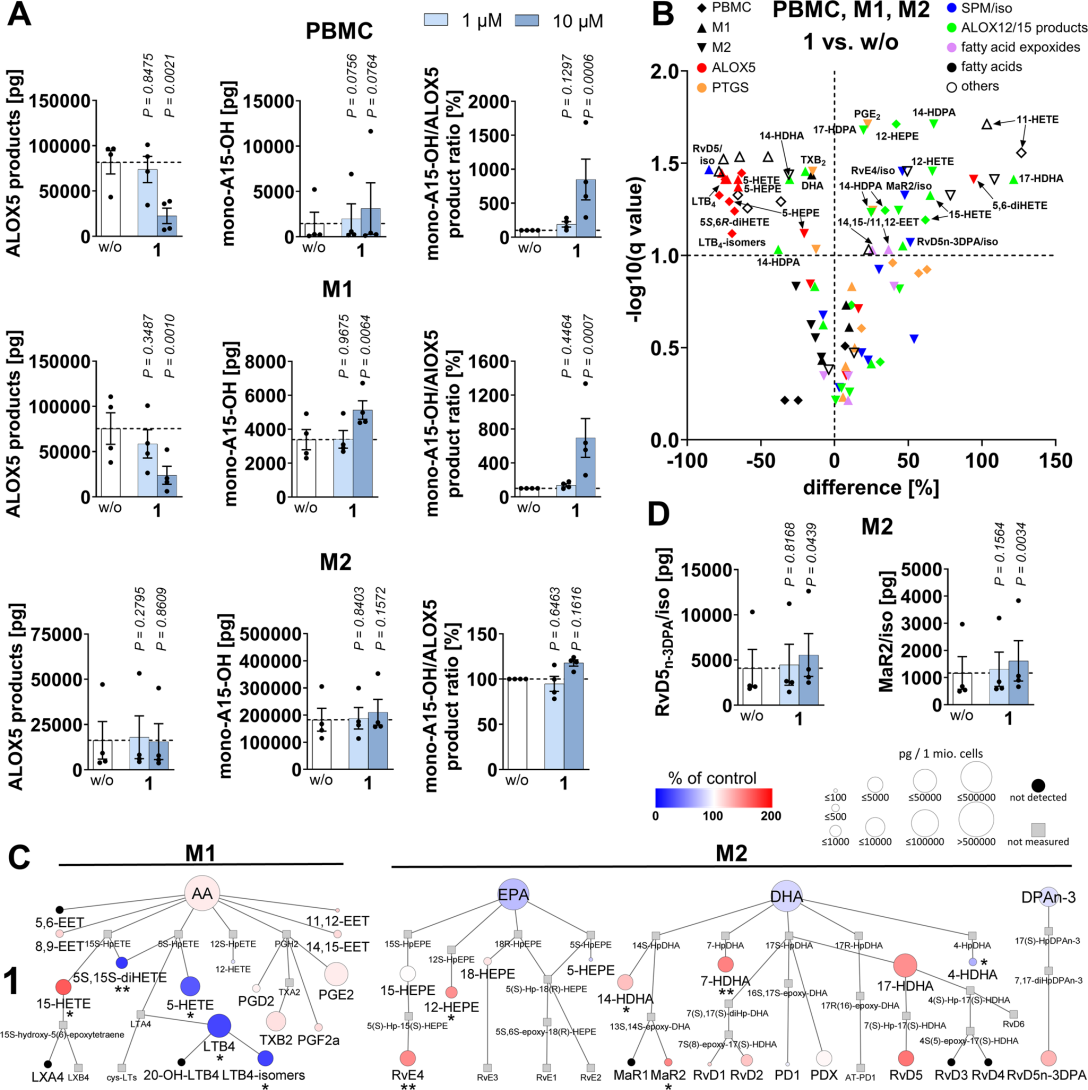
Compound **1** switches the lipid mediator profile of activated innate immune cells toward inflammation suppression and resolution. (A, B) PBMCs were preincubated (10 min) with vehicle (DMSO, 0.1%) or **1** (10 µM unless otherwise indicated) and then challenged with A23187 (10 min). (A–D) M1‐ or M2‐like macrophages were preincubated (15 min) with vehicle (DMSO, 0.1%) or **1** (10 µM unless otherwise indicated) and stimulated with SACM (180 min). (A) ALOX5 products and mono‐A15‐OH levels [per 5 × 10^6^ PBMCs or 2 × 10^6^ M1/M2]. (B) The volcano plot depicts the mean percentage difference relative to vehicle control and the negative log_10_(*q* value) calculated vs. vehicle control; two‐tailed multiple paired Student *t* tests with correction for multiple comparisons (false discovery rate 10%). (C) Pathway diagram showing percentage changes in lipid mediator formation upon treatment with **1** (10 µM) relative to vehicle control. Data for LXA_4_, RvE4, MaR2, RvD1, RvD2, PD1, PDX, RvD5 and RvD5_n‐3DPA_ may include unresolved isomeric species. (D) SPM/iso levels [per 2 × 10^6^ M2]. Mean (B, C) or mean + SEM and single data (A, D) from n = 3–4 (B,C), n = 4 (A, D) independent experiments. *P* values (A–D) or *p < 0.05, **p < 0.01 (C) given vs. vehicle control; repeated measures one‐way ANOVA of log data + Dunnett *post hoc* tests (A, D) or two‐tailed paired Student *t* test of log data (C).

Since PBMCs exhibit a relatively low capacity to produce ALOX5‐derived LTs and ALOX15‐derived SPMs compared to more specialized immune cells such as macrophages [36, 61, 62, 63], we focused subsequent studies on activated monocyte‐derived M1‐ and M2‐like macrophages. To this end, human monocyte‐derived macrophages were polarized using supraphysiological cytokine concentrations [82, 83, 84, 85] and challenged with *Staphylococcus aureus* (LS1)‐conditioned medium (SACM) as a physiological stimulus [86]. M1‐like macrophages contribute to the initiation of inflammation and efficiently produce LTs and PTGS‐derived prostanoids, whereas M2‐like macrophages strongly express ALOX15 and generate a broad spectrum of SPMs [36], which have been proposed to promote the resolution of inflammation [33, 38]. Recent studies have shown that certain di‐ and trihydroxylated oxylipin isomers cannot be completely resolved on conventional non‐chiral chromatographic columns [44]. Accordingly, we collectively denote these analytes as SPMs and/or isomers (SPM/iso), although any species that differed in retention time or MS/MS fragmentation from the authentic standards were excluded from the analysis.

As expected from our findings in PBMCs, compound **1** (10 µM) induced a switch from ALOX5 product formation to mono‐A15‐OH formation in M1‐like macrophages (Figure 2A,C; Figures S1F and S2; Data Table S2) and to mono‐A12‐OH with a smaller increase in mono‐A15‐OH formation in M2‐like macrophages (Figure 2A–C; Figures S1F,G and S2; Data Table S2). As a consequence, SPM/iso levels increased in M2‐like macrophages, showing a trend toward higher total SPM/iso levels (*p* = 0.0810) (Figure S1G; Data Table S2), and reaching significance for MaR2/iso, RvE4/iso and RvD5_n‐3DPA_/iso (Figure 2B–D and Figure S1F). Activation of phospholipases A_2_ can be excluded as a primary mechanism driving ALOX12/15 product formation, as free PUFA levels were not or only minimally elevated (Figure 2C and Figure S1H).

The increase in mono‐A15‐OH formation in PBMCs was accompanied by enhanced biosynthesis of PTGS products such as PGE_2_ (Figure 2B and Figure S1E). Beyond its well‐known roles in promoting inflammation and pain, PGE_2_ also exerts anti‐inflammatory actions and initiates the switch to inflammation resolution [27]. These findings suggest that AA/20:4 is partially diverted from ALOX5 product formation toward other lipid mediator biosynthetic pathways, including prostaglandin biosynthesis [16]. In macrophages, effects on PTGS products were less pronounced or absent (Figure 2B,C; Figures S1F and S2).

Together, compound **1** establishes a pro‐resolving lipid mediator profile in innate immune cells by inducing the biosynthesis of SPM/iso, including ALOX12/15‐derived precursors, while increasing the availability of immunomodulatory prostanoids and suppressing the formation of pro‐inflammatory LTs and other ALOX5 products.

### 2.2 Structural Requirements for Promoting SPM/Iso Production

Starting from compound **1**, we assessed eight structural derivatives (Figure 1) for their ability to induce a lipid mediator class switch in activated PBMCs as well as M1‐ and M2‐like macrophages. These derivatives feature modifications within the *p*‐quinone moiety (**2–4**), incorporate a *cis*‐decalin scaffold (**5**‐**7**), or represent synthetic fragments obtained during total synthesis (**8**, **9**). Notably, the pentacyclic meroterpenoid stachyflin (**6**) is a secondary metabolite from the mold *Stachybotrys sp*. RF‐7260 and has been reported to possess antiviral activity [78, 80].

The NH_2_ group in **1** is essential for potent inhibition of ALOX5 product formation in PBMCs and M1‐like macrophages (**3**). Replacement with another H‐bond acceptor, such as a methoxy (**2**) or alcohol group (**4**), is tolerated in PBMCs, even in the absence of additional functional groups on the upper ring, but not in M1‐like macrophages (**4**) (Figure 3A,B; Figures S2 and S3). Conversion of the *trans*‐ into a *cis*‐decalin scaffold together with the introduction of an alcohol group at the 3‐position (**5**) is also tolerated, as is annulation of the benzene ring with γ‐lactam (**6**, **7**). Interestingly, the fragment **8**, which contains a desaturated decalin, retains ALOX5‐inhibitory activity in PBMCs (but not in M1‐like macrophages), whereas the synthetic building block representing the upper ring system (**9**) shows minimal activity (Figure 3A,B; Figures S3 and S4). ALOX5 product formation is less pronounced in M2‐ than in M1‐like macrophages (Data Table S2) [36] and is also less potently inhibited by meroterpenoids **1–7** in this phenotype (Figures S2 and S5).

**FIGURE 3:**
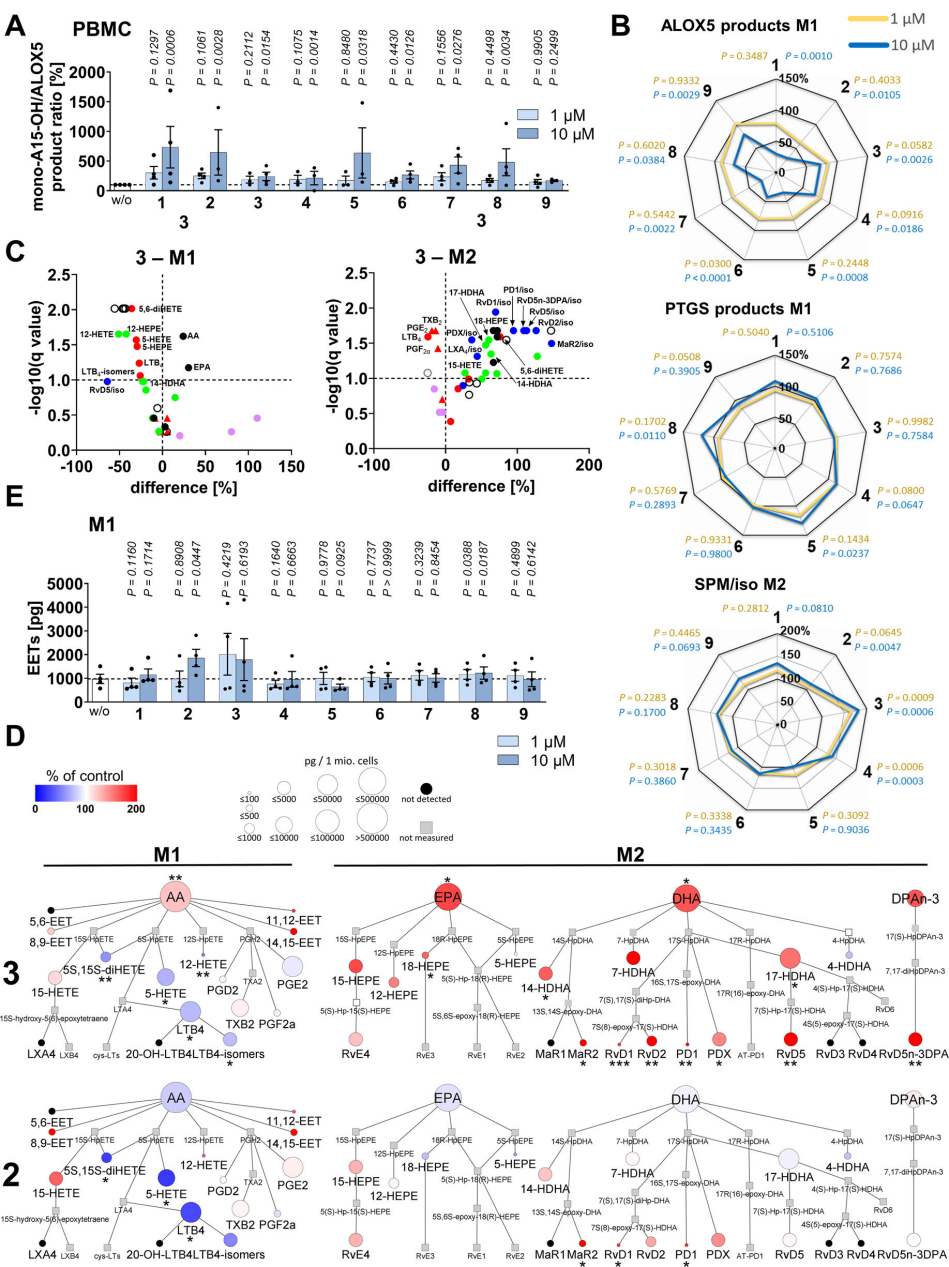
Meroterpenoid derivatives induce anti‐inflammatory and pro‐resolving changes in the lipid mediator profile of activated immune cells. (A) Mono‐A15‐OH/ALOX5 product ratio of PBMCs following 10 min preincubation with vehicle (DMSO, 0.1%) or test compounds and subsequent treatment with the calcium‐ionophore A23187 (10 min) [per 5 × 10^6^ PBMCs]. Data for vehicle control and **1** are identical to Figure 2A. (B–E) M1‐ or M2‐like macrophages were preincubated (15 min) with vehicle (DMSO, 0.1%) or test compounds (10 µM unless otherwise indicated) and activated with SACM (180 min). (B) Levels of ALOX5 products, PTGS products, and SPM/iso. The vehicle control and data for **1** are identical to Figure 2B,C (SPM/iso). (C) Volcano plots depict the mean percentage difference relative to vehicle control and the negative log_10_(*q* value) calculated vs. vehicle control; two‐tailed multiple paired Student *t* tests with correction for multiple comparisons (false discovery rate 10%). (D) Pathway diagrams illustrating percentage changes relative to vehicle control in the lipid mediator profile of M1‐ and M2‐like macrophages treated with **2** (10 µM) or **3** (10 µM). Data for LXA_4_, RvE4, MaR2, RvD1, RvD2, PD1, PDX, RvD5, and RvD5_n‐3DPA_ may include unresolved isomeric species. E) EET levels [per 2 × 10^6^ M1]. Mean (B, C, D) or mean + SEM and single data (A, E) from n = 3–4 independent experiments. *P* values (A‐E) or *p < 0.05, **p < 0.01, ***p < 0.001 (E) given vs. vehicle control; repeated measures one‐way ANOVA of log data or mixed‐effects model (REML) of log data + Dunnett *post hoc* tests (A, B, E) and two‐tailed paired Student *t* test of log data (D).

The ratio of mono‐A15‐OH to ALOX5 products increased in PBMCs treated with **1** or its derivatives (1 and 10 µM, except for the truncated **9**) (Figure 3A). Among these, the *trans*‐decalins (**1–4**) preferentially upregulated mono‐A15‐OH in addition to, or instead of, suppressing ALOX5 product formation (Figures S1E and S3). Similar results were obtained in M2‐like macrophages, where enhanced ALOX12/15 product formation translated into increased SPM/iso biosynthesis (**3**> **4**> **1**, **2**) (Figure 3B,C; Figures S5 and S6A). Interestingly, structural variants allowed partial discrimination between SPM/iso subclasses (Figures S2 and S6A), possibly due to differences in subcellular distribution and, consequently, access to biosynthetic enzymes [87, 88, 89]. Compound **2** (10> 1 µM) was particularly effective in elevating PD1/iso and MaR2/iso levels; compound **3** (1 and 10 µM) robustly increased the production of all major SPM/iso species (including Rv/iso, PD1/iso, MaR2/iso, and LXA_4_/iso), and the otherwise less active *cis*‐decalin **5** (1 and 10 µM) specifically induced PD1/iso biosynthesis (Figure S6A). Mechanistically, the enhanced SPM/iso production by **3** appears to depend (at least partially) on the release of PUFAs and the formation of ALOX12/15‐derived SPM precursors such as 17‐HDHA (D‐series Rv and PD precursor), 14‐HDHA (MaR precursor), and 15‐HETE (LX precursor). In contrast, the stimulatory effect of **2**, which primarily increases PD1/iso, RvD1/iso, and MaR2/iso formation, is not associated with substantially elevated levels of SPM precursors or free PUFAs (Figure 3D; Figures S2, S5, and S6A). These findings imply that stimulated PUFA release by (specific) meroterpenoids contributes to the efficient induction of SPM/iso biosynthesis in innate immune cells, but is not the principal driving mechanism.

The moderate stimulatory effect of **1** on the biosynthesis of PTGS‐derived PGE_2_ and/or other prostanoids was also evident for derivatives **2–9** (10 µM), although the efficiency of inducing PTGS product formation varied considerably between activated innate immune cell types and prostanoid species (Figure 3B; Figures S1E,F, S4, and S5). Individual prostanoid levels (PGE_2_, PGF_2α_, and/or TxB_2_) or total PTGS products were significantly elevated in PBMCs by **2**, **3**, and **5–8** (Figure S3), in M1‐like macrophages by **4**, **5**, and **8** (Figure 3B and Figure S4), and in M2‐like macrophages by **5**, **7**, and **8** (Figure S5). For other compounds or experimental settings, prostanoid production was either minimally affected or decreased (Figures S1E and S2), particularly in M2‐like macrophages treated with **3** (10 µM; Figure 3C).

The ability of meroterpenoids (10> 1 µM) to increase anti‐inflammatory EET levels was markedly enhanced (specifically in M1‐like macrophages) when the amine in **1** was replaced with a methoxy group (**2**). Other derivatives showed little activity, and compound **3** produced variable results across independent datasets derived from immune cells of different platelet donors (Figure 3E, Figures S4 and S6B).

Conclusively, structural modification of **1** yielded derivatives that outperform the parent compound in inducing a favorable lipid mediator class switch from LTs to SPM/iso (**2**‐**4**) and, in part, to EETs (**2**) in polarized macrophages. Compound **2** preferentially elevates PD1/iso, MaR2/iso, and EET levels, whereas compound **3** potently enhances SPM/iso biosynthesis across multiple subclasses (Figure 3D).

### 2.3 Meroterpenoids Actively Trigger SPM/Iso Biosynthesis in Resting Macrophages

The small β‐pore‐forming toxin α‐hemolysin present in SACM induces mono‐A15‐OH and SPM formation in M2‐like macrophages in a metallopeptidase domain 10 (ADAM10)‐dependent manner [86]. We next asked whether meroterpenoids sensitize cells to SACM or whether they activate SPM biosynthesis independently of SACM. To address this question, we exposed non‐activated M2‐like macrophages to **2** and **3** and monitored their lipid mediator profiles. Both compounds potently induced SPM/iso production already at 1 µM, accompanied by a trend towards enhanced ALOX12/15 and PTGS product formation (Figure 4A and Figure S7A; Data Table S3). At higher concentrations (10 µM), both derivatives increased the availability of free PUFAs, whose release likely contributes to the overall induction of lipid mediator biosynthesis, while still favoring the formation of ALOX12/15 products and particularly SPM/iso (Figure 4B and Figure S7B). As a result, the total amount of SPM/iso equaled or even surpassed the low basal levels of ALOX5 products, potentially reaching physiologically relevant concentrations (Figure S7B).

**FIGURE 4:**
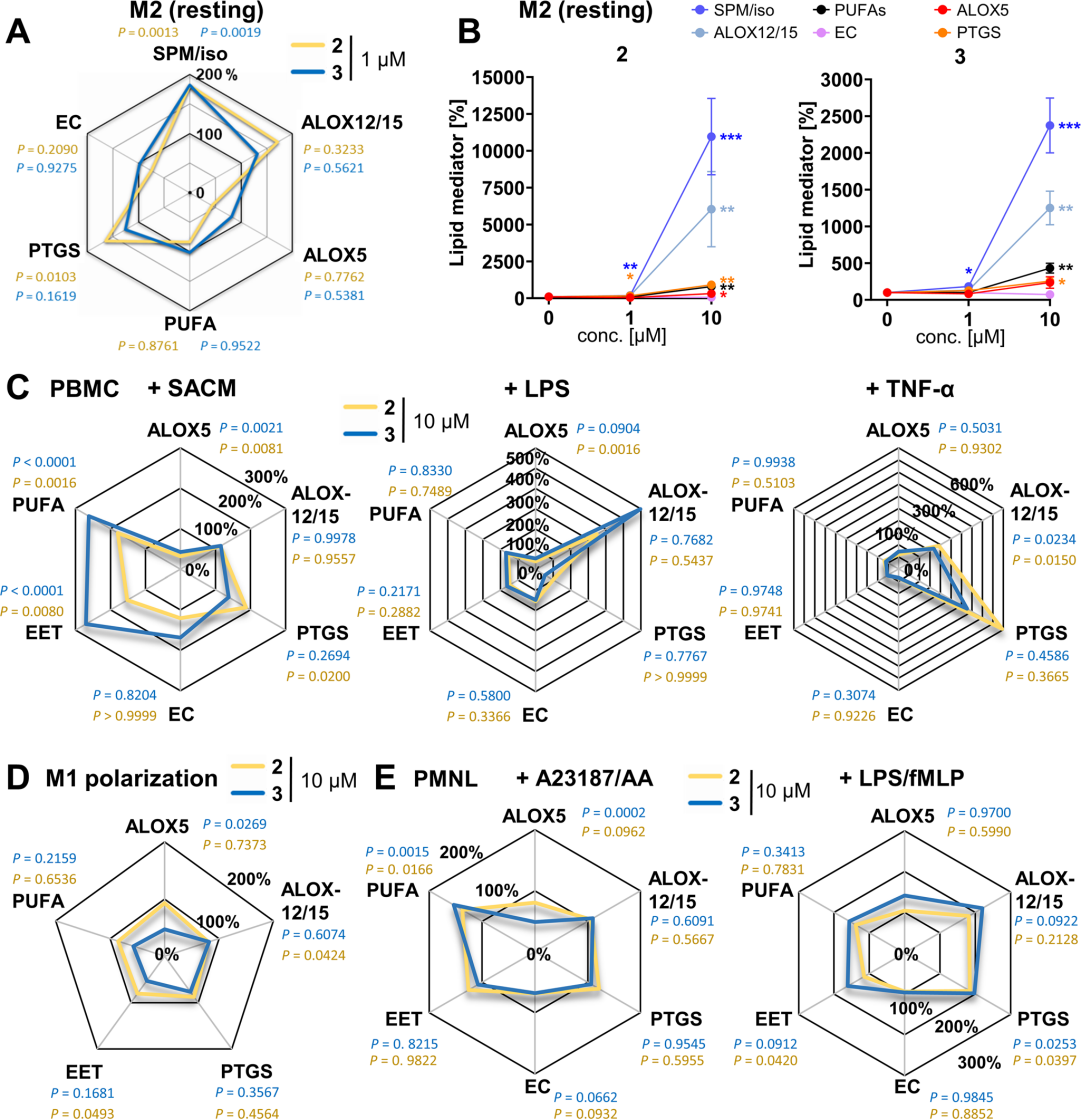
Meroterpenoids context‐dependently manipulate lipid mediator profiles of innate immune cells depending on cell type and stimulation condition. Percentage changes in PUFA and lipid mediator class levels are shown. Lipid mediators belonging to each subclass are defined in Figures S7A, S8A, S9A, and S10A. (A, B) Resting M2‐like macrophages treated with vehicle (DMSO, 0.1%), **2**, or **3** (1 µM each) for 195 min. (C) PBMCs pretreated (10 min) with vehicle (DMSO, 0.1%), **2**, or **3** (10 µM each) and stimulated with SACM (1%, 180 min), LPS (0.1 µg/mL, 24 h), or TNF‐α (20 ng/mL, 24 h). (D) Monocyte‐derived macrophages pretreated with vehicle (DMSO, 0.1%), **2**, or **3** (10 µM each) and polarized for 48 h to M1‐like macrophages (100 ng/mL LPS + 20 ng/mL interferon‐γ) or to M2‐like macrophages (20 ng/mL interleukin‐4) before being stimulated with SACM (180 min). (E) PMNL preincubated (10 min) with vehicle (DMSO, 0.1%), **2**, or **3** (10 µM each) and treated with A23197 (2.5 µM) and AA/20:4 (20 µM) for 10 min (left panel). Alternatively, PMNL were primed with LPS (1 µg/mL, 10 min), pre‐incubated (30 min) with vehicle (DMSO, 0.1%), **2**, or **3** (10 µM each), and stimulated with fMLP (1 µM) for 5 min (right panel). Mean (A, C‐E) or mean + SEM (B) from n = 3–5 (A,B), n = 4 (C, D), or n = 5 (E) independent experiments. *p < 0.05, **p < 0.01, ***p < 0.001 or *p* values given vs. vehicle control; repeated measures one‐way ANOVA of log data (A, C–E) + Dunnett *post hoc* tests and two‐tailed paired Student *t* test of log data (B).

### 2.4 The Lipid Mediator Response of Innate Immune Cells Depends on the Activation Context

The type of innate immune cell and the stimulation condition strongly influence the spectrum of lipid mediators produced [90]. We therefore examined the effects of the meroterpenoids **2** and **3** on lipid mediator profiles under various stimulation conditions in the two major innate immune cell fractions isolated from blood, PBMCs and PMNLs (the latter mainly consisting of neutrophils). PBMCs were activated either by Ca^2^^+^ influx induced with the ionophore A23187 (10 min; Figure S1E), by pore‐forming bacterial toxins (SACM, 3 h; Figure 4C and Figure S8; Data Table S4), by the endotoxin LPS (24 h; Figure 4C and Figure S9; Data Table S5), or by the pro‐inflammatory cytokine tumor necrosis factor (TNF)‐α (24 h; Figure 4C and Figure S10; Data Table S6). To assess lipid mediator changes during macrophage polarization, we also analyzed macrophages stimulated with LPS plus IFNγ to induce M1‐like and with IL‐4 to induce M2‐like phenotypes (48 h; Figure 4D and Figure S11; Data Table S7). For PMNLs, meroterpenoid‐treated cells were either challenged with A23187 (10 min) to assess their lipid mediator‐forming capacity or primed with LPS and subsequently exposed to the physiological stimulus *N*‐formylmethionine‐leucyl‐phenylalanine (fMLP, 5 min; Figure 4E and Figure S12; Data Table S8). The effects of meroterpenoids on M1‐ and M2‐like macrophage subtypes have already been described above (Figures 3B–E and 4A,B).

Our data indicate that the effects of meroterpenoids on the lipid mediator profile are strongly context‐dependent. For example, ALOX5 product formation was potently inhibited in A23187‐ and SACM‐treated PBMCs (Figure 4C, Figures S3 and S8), SACM‐stimulated M1‐like macrophages (Figure 3B), SACM‐stimulated macrophages exposed to meroterpenoids during polarization towards the M1‐like subtype (only by **3**; Figure 4D and Figure S11), and A23187 plus AA/20:4‐activated PMNLs (preferentially by **3**; Figure 4E and Figure S12). In contrast, inhibition was less pronounced in LPS‐primed and fMLP‐activated PMNLs (Figure 4E and Figure S12) and in LPS/fMLP‐treated PBMCs (Figure 4C and Figure S9), and absent in SACM‐stimulated M2‐like macrophages (Figure S2), SACM‐stimulated macrophages exposed to meroterpenoids during polarization towards M2‐like phenotypes (Figure S11), LPS/fMLP‐treated PMNL (Figure 4E and Figure S12), and TNF‐α‐treated PBMCs, where ALOX5 product formation was not efficiently induced by the stimulus itself (Figure 4C and Figure S10).

The induction of PTGS product formation by meroterpenoids was particularly strong in PBMCs, especially when treated with A23187 (Figure S3), and in LPS/fMLP‐activated PMNL (Figure 4E and Figure S12). The effect was associated with high variability in TNF‐α‐stimulated PBMCs and was less pronounced following SACM treatment (Figure 4C and Figure S8) as well as in A23187 plus AA/20:4‐treated PMNLs (Figure 4C and Figure S10). In contrast, during prolonged LPS treatment of PBMCs, compound **3** (but not **2**) rather reduced PTGS product formation, reaching significance for PGF_2α_ (Figure 4C and Figure S9).

The increase in ALOX12/15 products was (neglecting varying efficacies) somewhat more consistent throughout the systems (Figures 3A and 4C,E and Figures S1E, S2, S9, S10, and S12), except for SACM‐stimulated PBMCs (Figure 4C and Figure S8), A23187 plus AA/20:4‐treated PMNL (Figure 4E), and SACM‐stimulated macrophages exposed to meroterpenoids during polarization (Figure 4D and Figure S11), where meroterpenoids were without substantial effect.

The increase of EET levels observed in SACM‐stimulated M1‐like macrophages (Figure 3E) was also found for PBMCs exposed to the same stimulus (Figure 4C and Figure S8) and LPS/fMLP‐stimulated PMNL (Figure 4E and Figure S12), whereas LPS was less effective in PBMCs (Figure 4C and Figure S9). Endocannabinoid levels were specifically increased by meroterpenoids in SACM‐, but not LPS‐ or TNF‐α‐treated PBMCs (Figure 4C and Figures S8–S10), and even tended to decrease in A23187 plus AA/20:4‐ (but not LPS/fMLP‐) activated PMNLs (Figure 4E and Figure S12). This decrease is likely due to inhibition of ALOX5 product formation, as the effect was mimicked by the ALOX5 inhibitor zileuton (Figure S12). In many, but not all systems, meroterpenoid‐induced lipid mediator production was accompanied by the release of PUFAs, most prominently in SACM‐stimulated PBMCs and A23187 plus AA/20:4‐treated PMNL (Figure 4C and Figure S8).

Together, these data indicate that while general principles of meroterpenoid action are conserved across innate immune cell types and stimulation conditions, their precise impact on the lipid mediator profile is highly context‐dependent and, in some cases, differs between individual meroterpenoids.

### 2.5 Meroterpenoids Use Different Strategies to Inhibit ALOX5 Product Formation

The meroterpenoids **3–5** directly and, as shown for **3**, reversibly inhibit human ALOX5 (IC_50_ = 1‐1.7 µM; Figure 5A,B) and are even more effective in cell‐free than in cell‐based assays (Figures 3B and 5A; Figure S3). Enzyme kinetic analysis revealed a predominantly competitive mode of ALOX5 inhibition for **3**, with indications of a mixed‐type non‐competitive component (Figure 5C and Figure S13). Non‐specific inhibition via detergent‐sensitive colloid‐like aggregates [91] could be excluded for **3** (3 µM), as ALOX5 inhibition was unaffected by Triton X‐100 (Figure 5B). All other active derivatives, including the natural products **1** and **6**, suppressed ALOX5 product formation in innate immune cells even more potently (Figures 3B and 5A; Figure S3), pointing to another high‐affinity target within the ALOX5 pathway. The addition of Triton X‐100 further implied that the moderate inhibition of cell‐free ALOX5 by **2** may be artifactual (Figure 5B). Given the overall decrease in ALOX5 products, which is not limited to LTB_4_, LTA_4_ hydrolase was excluded as a target (Figures S1E and S2). Instead, our results are consistent with ALOX5AP antagonism, as exogenous AA/20:4 diminished the inhibitory effect of **1** and **2** on ALOX5 product formation in PBMCs (Figure 5D). ALOX5AP, an integral nuclear membrane protein, transfers AA/20:4 from cPLA_2α_ to ALOX5 and is essential for ALOX5 product formation unless AA/20:4 is externally supplied [92]. Accordingly, **2** and **3** (10 µM, each) suppressed LTB_4_ formation in ALOX5–transfected HEK‐293 cells, which otherwise lack endogenous ALOX5 activity, while co‐expression of ALOX5AP increased LTB_4_ formation and enhanced the sensitivity to **2** over **3** (Figure 5E). We therefore conclude that compound **3** acts as a direct and reversible ALOX5 inhibitor, whereas **2** primarily functions as a ALOX5AP antagonist.

**FIGURE 5:**
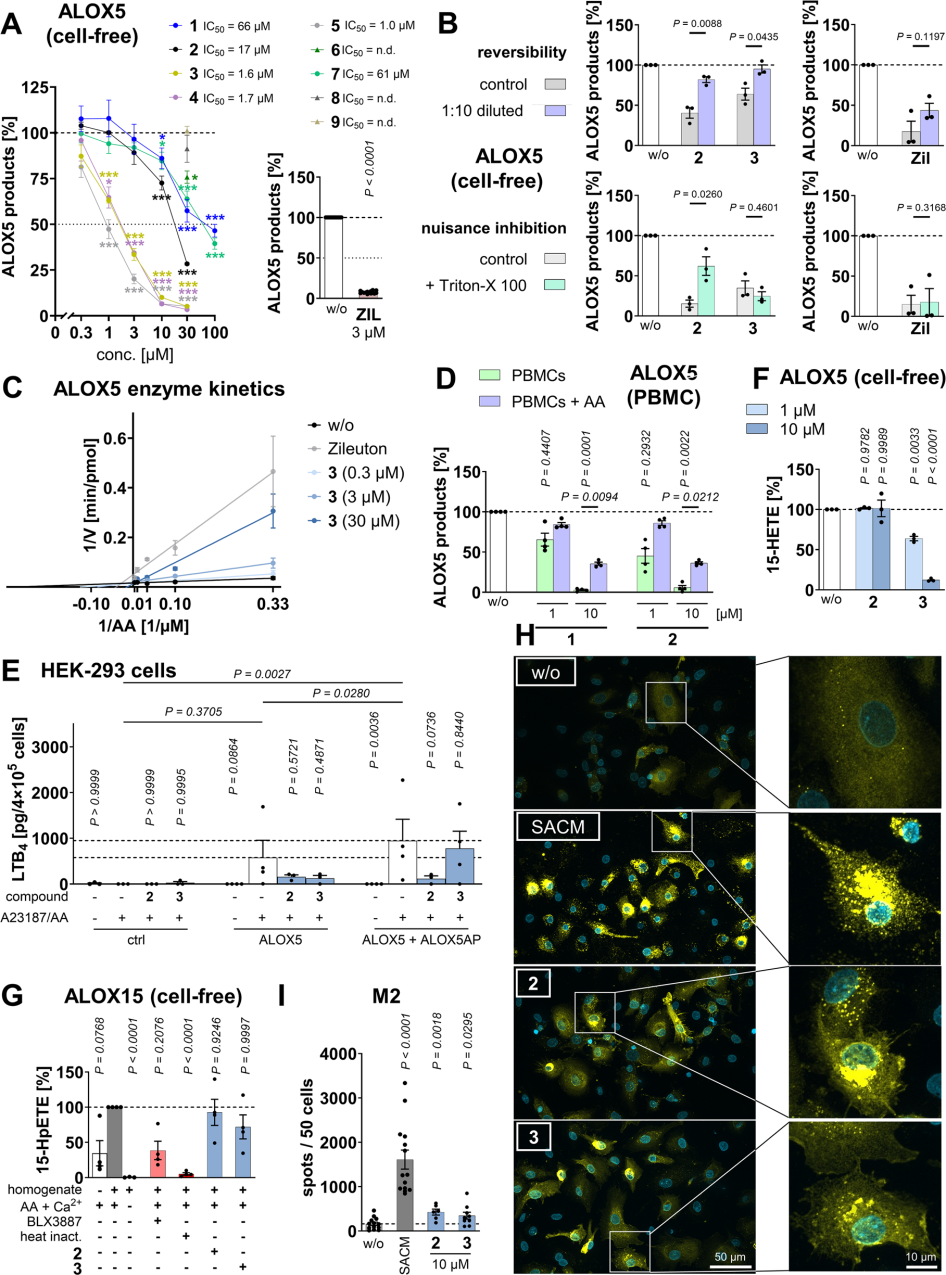
Specific meroterpenoids differentially suppress ALOX5 product formation while inducing ALOX15 translocation. (A, F). Human recombinant ALOX5 was pretreated (10 min) with vehicle (DMSO, 0.1%), zileuton (‘ZIL’), or test compounds. Product formation was initiated by addition of AA/20:4 and CaCl_2_ (10 min). A) Concentration‐dependent effects on total ALOX5 product formation. (B) Effect of Triton X‐100 (0.01%) on ALOX5 inhibition by vehicle (DMSO, 0.1%), **2** (30 µM), or **3** (3 µM). Reversibility of ALOX5 inhibition by **2** (30 µM) and **3** (3 µM) was assessed by pretreating samples (10 min), diluting 10‐fold, incubating for 2 min, and then adding of AA/20:4. (C) Lineweaver–Burk plot generated from activity data of human recombinant ALOX5 treated with vehicle (DMSO, 0.1%), zileuton (3 µM), or **3** (0.3, 3, 30 µM) at varying AA/20:4 concentrations (3, 10, 30, 100, 300 µM). (D) PBMCs were preincubated (10 min) with vehicle (DMSO, 0.1%), **1**, or **2**, and ALOX5 product formation (LTB_4_, LTB_4_‐isomers, 20‐OH‐LTB_4_, 5,6‐diHETE, and 5‐HEPE) was induced by calcium ionophore (A23187) in the presence or absence of AA/20:4. (E) LTB_4_ formation in HEK‐293 cells transfected with a control vector (“ctrl”) or expression vectors encoding ALOX5 or ALOX5AP. Cells were either left unstimulated or preincubated (15 min) with vehicle (DMSO, 0.1%), **2**, or **3** (10 µM) and subsequently treated with A23187 (2.5 µM) and AA/20:4 (2 µM) for 15 min [per 4 × 10^5^ cells]. (F) Conversion of AA/20:4 to 15‐HETE by ALOX5. (G) 15‐HpETE formation (reflecting ALOX15 activity) in M2‐like macrophage homogenates preincubated (15 min) with vehicle (DMSO, 0.1%) or test compounds (10 µM) and then treated with AA/20:4 and CaCl_2_ (15 min). (H, I) M2‐like macrophages treated with vehicle (DMSO, 0.1%), SACM (0.5%), **2** (10 µM), or **3** (10 µM) for 90 min. (H) Immunofluorescence images are stained for ALOX15 (yellow, exposure time 300 ms) and nuclei (DAPI, light blue, exposure 20 ms). Images are representative of > 100 single cells analyzed in n = 3 independent experiments. (I) Quantification of ALOX15‐1 translocation‐derived spots, normalized to cell number. Mean + SEM (A, C) and single data (A, B, D–G, I) from n = 3 (A, B, F, H, I), n = 3–4 (E, G), n = 4 (D), n = 5 (C), n = 15 (A ZIL) independent experiments. *p < 0.05, **p < 0.01, ***p < 0.001 or *p* values given vs. the respective vehicle control or as indicated by bars; repeated measures one‐way ANOVA of log data (A **2–5**,D,F) or mixed‐effects model (REML) of log data (A **1** and **7**,G) + Dunnett *post hoc* tests, two‐way ANOVA of log data (B) or repeated measures mixed‐effects model (REML) (E) + Dunnett *post hoc* tests (B, E) or Tukey *post hoc* tests (E bars), and two‐tailed paired Student *t* test (I) of log data (A **6**, **8–9**, ZIL, B ZIL, D for pairwise comparisons).

### 2.6 Induction of SPM/Iso Biosynthesis is Associated With ALOX15 Translocation

Only a few small molecules are known to stimulate SPM/iso biosynthesis in non‐activated macrophages, [59, 60, 61, 75] among them AKBA (3‐O‐acetyl‐11‐keto‐β‐boswellic acid), which shifts the regiospecificity of cell‐free ALOX5 toward 12/15‐lipoxygenation and induces SPM/iso biosynthesis in HEK‐293 cells stably expressing ALOX5 and ALOX5AP [93]. Such an activity was excluded for **2** and **3** (≤ 10 µM), which not only failed to enhance the conversion of AA/20:4 to 12‐HETE and 15‐HETE by human recombinant ALOX5 (Figure 5F and Figure S14A), but, in case of **3** (10> 1 µM), even significantly reduced this side reaction.

Using the selective ALOX15 inhibitor BLX3887, we confirmed that ALOX15 essentially contributes to SPM precursor (i.e., 15‐HETE) formation, both in vehicle‐ and **2**‐treated SACM‐activated M2‐like macrophages (Figure S14B). Conclusions regarding the ability of **2** to further increase 15‐HETE formation in the presence of the inhibitor are limited by the moderate effect and high inter‐donor variability. We next investigated whether **2** and **3** (10 µM) directly activate human ALOX15, a key enzyme in SPM biosynthesis [36, 59], which was not the case (Figure 5G and Figure S14C).

SACM, as well as previously reported small molecules that actively trigger SPM/iso biosynthesis, promote the redistribution of ALOX15 to particulate sites in the cytoplasm [59, 61, 67, 75, 86]. Similarly, **2** and **3** (10 µM) efficiently stimulate ALOX15 translocation in M2‐like macrophages (Figure 5H) and increase the number of ALOX15‐enriched cytoplasmic particles (Figure 5I), a process considered to initiate SPM precursor formation [35, 36, 94]. Notably, SACM is more effective than the meroterpenoids in both inducing ALOX15 translocation (Figure 5H,I) and triggering SPM/iso biosynthesis (Data Tables S2 and S3), but also exhibits cytotoxicity upon prolonged exposure [86]. In conclusion, **2** and **3** seemingly enhance SPM/iso production in resting macrophages by coordinating the subcellular localization of ALOX15 (Figure 5H,I), possibly acting in synergy with PUFA release at higher concentrations (Figure 4B).

### 2.7 Concomitant Suppression of Cytokine Production

Cytokines play a pivotal role in coordinating inflammatory responses and act in close crosstalk with lipid mediators [95]. We therefore investigated the effect of meroterpenoids on the production of five pro‐inflammatory (TNF‐α, IL‐1β, IL‐6, IL‐12 (p70), IL‐23), two anti‐inflammatory cytokines (IL‐10 and IL‐1ra), and two chemokines (MCP‐1, IL‐8) in LPS‐stimulated PBMCs. Overall, meroterpenoids (**1–7**; 10> 1 µM) moderately reduced cytokine and chemokine expression, with the exception of MCP1, whose levels tended to increase, especially upon treatment with **1** (Figure 6A and Figure S15).

**FIGURE 6:**
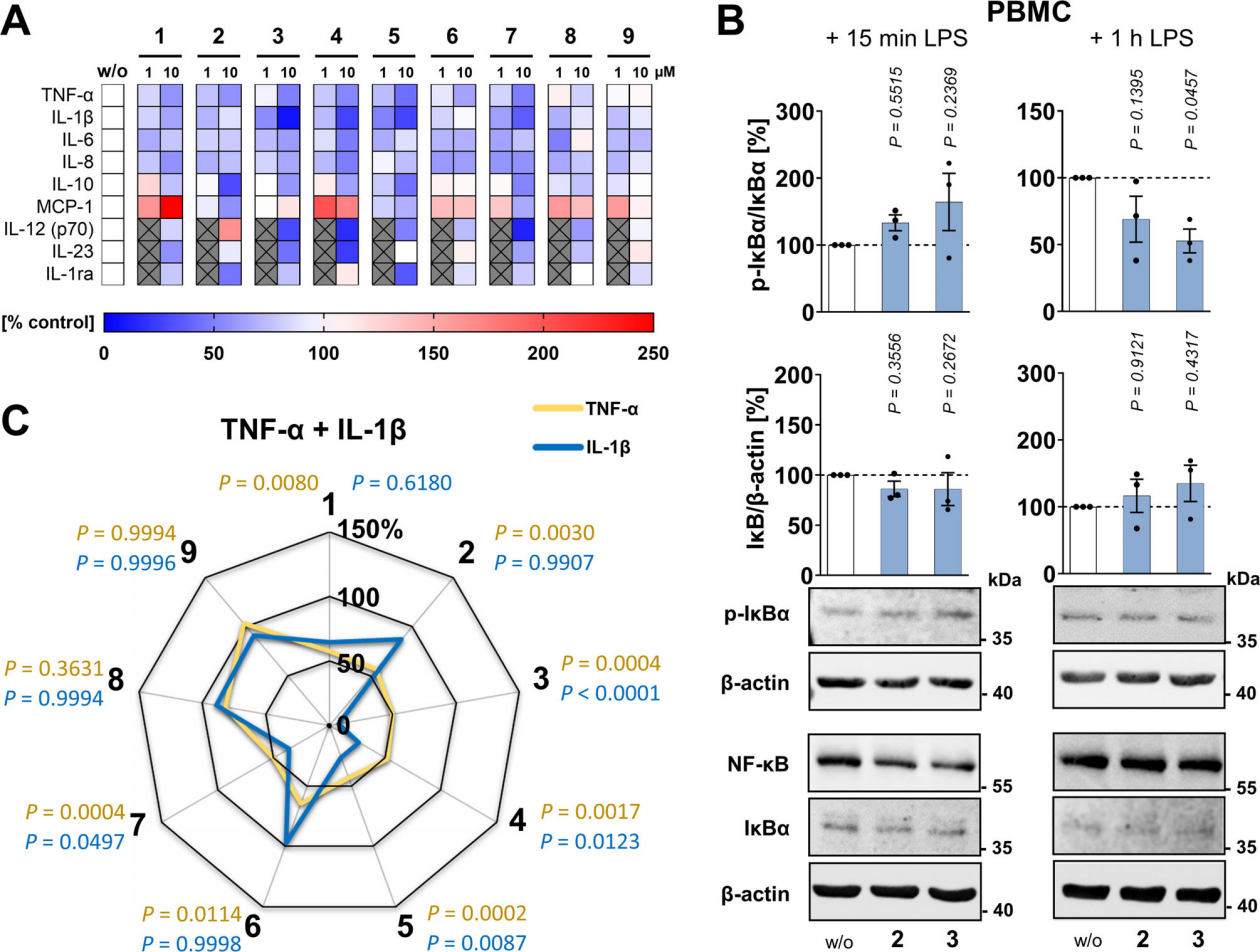
Meroterpenoids interfere with the production of inflammatory cytokines and chemokines. PBMCs were pretreated (30 min) with vehicle (DMSO, 0.1%) or test compounds (10 µM unless otherwise indicated) and subsequently stimulated with LPS for 4 h (TNF‐α, IL‐8), 18 h (IL‐1β, IL‐6, IL‐10, MCP‐1, IL‐12 (p70), IL‐23, IL‐1ra), or for the durations indicated. A) The heatmap depicts changes in cytokine and chemokine profiles expressed as percentage of the vehicle control. B) Cellular IκBα phosphorylation, IκBα levels, and NF‐κB expression. Western blots are representative of n = 3 independent experiments. Uncropped blots are shown in Figure S17A,B (A 15 min LPS, B 1 h LPS). The vehicle controls are identical to [98]. C) Radar chart depicting percentage changes in TNF‐α and IL‐1β expression relative to vehicle control. Mean (A,C) or mean + SEM and single data (B) from n = 3 (B,C), n = 3–4 (A) independent experiments. *P* values given vs. vehicle control (B,C); repeated measures one‐way ANOVA (B) of log data (C IL‐1β) or mixed‐effects model (REML) of log data (C TNF‐α) + Dunnett *post hoc* tests and two‐tailed paired Student *t* test for pairwise comparisons as indicated by bars.

Mechanistically, compounds **3** and, by trend, **2** (10 µM) interfered with the activation of NF‐κB, a central transcription factor driving pro‐inflammatory cytokine expression [96, 97]. Both derivatives suppressed LPS‐induced phosphorylation of inhibitor of κ light chain gene enhancer in B cells α (p‐IκBα) at 1 h, following a transient increase at 15 min, without yet inducing its proteasomal degradation (Figure 6B and Figure S16). Notably, derivatives **3**–**5** and **7** exerted effects preferentially on specific cytokines. Compounds **3**–**5** robustly and preferentially reduced IL‐1β production and additionally decreased either IL‐12 (**3**, **4**), IL‐23 (**4**), or IL‐1ra (**5**) levels (Figure 6C and Figure S15), suggesting that cytokine modulation involves additional mechanisms beyond impaired NF‐κB signaling.

In conclusion, meroterpenoids attenuate cytokine expression in LPS‐stimulated PBMCs by dampening NF‐κB signaling and, possibly, through further yet‐unresolved pathways. While these effects are consistent with a reduced activation state of innate immune cells, it remains elusive whether the diminished cytokine production is secondary to the preceding lipid mediator class switch.

### 2.8 Changes in the Lipid Mediator Profile in Relieved Murine Peritonitis

Whether **2** and **3** induce a lipid mediator class switch from inflammation to resolution in vivo was investigated for zymosan‐induced murine peritonitis (Figure 7A). Initiation, progression, and resolution of inflammation in this self‐resolving model [99] depend, in addition to cytokines [100, 101], essentially on lipid mediators, specifically i) LTs [102, 103] and prostaglandins [104], which promote inflammation, ii) EETs [105, 106] and SPMs [100, 107], which limit or resolve inflammation, and iii) S1P [9, 108, 109] and potentially endocannabinoids [110, 111, 112], which exert immunomodulatory functions. To assess the bioavailability of the meroterpenoids, we measured the concentrations of **2** and **3** (10 mg/kg, i.p.) in plasma and peritoneal exudate at the acute phase (4 h) and the resolving phase (18 h). Compound **2** was detectable in both the peritoneal exudate and the systemic circulation at 4 h but its levels declined rapidly, whereas concentrations of **3** were maintained even 18 h after zymosan injection (Figure S18A). Notably, the peritoneal exudate and plasma concentrations of **2** and **3** (up to 2.6 and 1.0 µM in individual mice) were within the range found to modulate the lipid mediator profile in PBMCs and macrophage subtypes in vitro (Figure 3).

**FIGURE 7:**
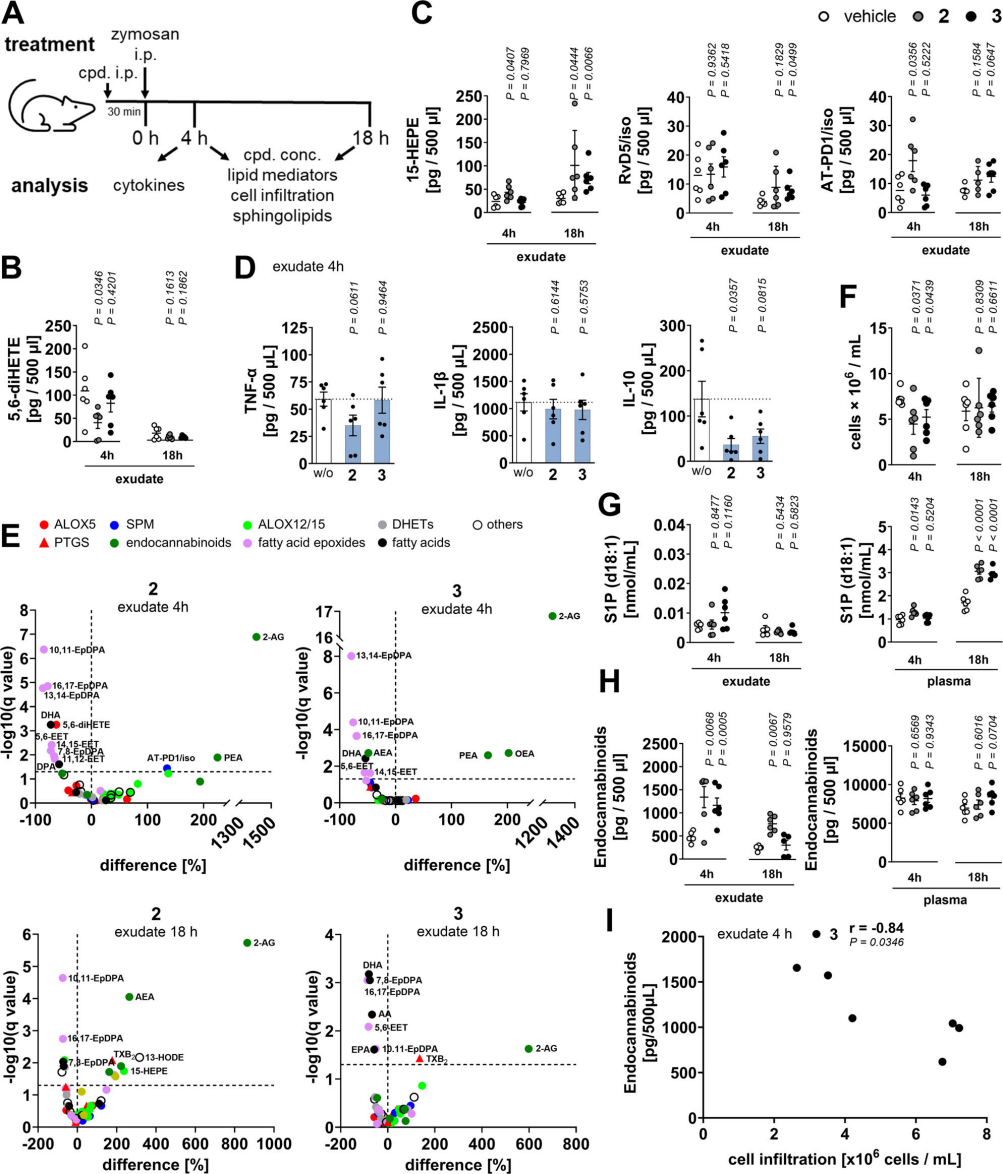
Alleviation of in vivo inflammation by **2** and **3** in murine peritonitis is associated with the reorganization of systemic and exudate lipid mediator profiles. (A) Time course and experimental readouts for zymosan‐induced murine peritonitis. (B–I) Mice received vehicle (2% DMSO in saline; 0.5 mL), **2**, or **3** (10 mg/kg, i.p.) 30 min prior to zymosan injection and were sacrificed after 4 h or 18 h for analyses of lipid mediators and cytokines in plasma and peritoneal exudate (B–E, G–I) and for quantification of immune cell infiltration (F). (B) 5,6‐diHETE. (C) 15‐HEPE, RvD5/iso, and AT‐PD1/iso. (D) TNF‐α, IL‐1β, and IL‐10. (E) Volcano plots depict mean percentage differences relative to vehicle control and ‐log_10_(*q* value) calculated vs. vehicle control; two‐tailed multiple paired Student *t* tests with correction for multiple comparisons (false discovery rate 5%). ((F) Immune cell infiltration. (G) Sphingosine‐1‐phosphate (S1P) (d18:1). (H) Total endocannabinoids. (I) Pearson correlation between peritoneal cell infiltration and total endocannabinoid concentrations, calculated from mean cell numbers and mean endocannabinoid levels. Mean (E, I) or mean + SEM and single data (B–D, F–H) from n = 5–6 (B, C, E, G), n = 6 (D,F,H,I) mice. *P* values given vs. vehicle control (B–H); two‐tailed unpaired Student *t* test (B–D, F, H) of log data (G). The lipid mediators belonging to each subclass are defined in Figure S19.

Compound **2** reduced the exudate concentration of ALOX5‐derived products such as 5,6‐diHETE. However, the effects were restricted to certain species, and **3** showed no comparable inhibitory activity (Figure 7B; Figures S18B and S19; Data Table S9). This tendency toward reduced ALOX5 product levels was even more pronounced in plasma during the resolution phase (18 h) (Figure S18C). In contrast, the levels of specific ALOX12/15‐derived products and SPM/iso were elevated, reaching significance for 15‐HEPE (**2**, 4+18 h; **3**, 18 h), RvD5/iso (**3**, 18 h), and AT‐PD1/iso (**2**, 4 h; **3**, 18 h), mainly in the exudate (Figure 7C and Figure S18D). The general increase in ALOX12/15 products was heterogenous among oxylipin species and encompassed both 12‐ and 15‐lipoxygenated regioisomers. This finding is particularly noteworthy because murine ALOX15 orthologs, unlike their human counterparts, preferentially catalyze 12‐ over 15‐lipoxygenation, thereby shaping the regioisomeric product pattern [31]. Compound **2** (but not **3**) also tended to reduce TNF‐α, but not IL‐1β, levels in the exudate (Figure 7D). Altogether, and in line with the in vitro findings, **2** and **3** modulate the in vivo lipid mediator and cytokine profile in a manner that supports the termination and resolution of inflammation, although with limited efficacy and seemingly accompanied by adjustments that may counteract their overall anti‐inflammatory activity. In this context, both **2** and **3** depleted the peritoneal exudate of anti‐inflammatory mediators such as fatty acid epoxides and IL‐10 (Figure 7D,E).

Despite these mixed alternations in the exudate and systemic lipid mediator profiles with regard to anti‐inflammatory efficacy, both **2** and **3** substantially reduced immune cell infiltration into the peritoneal cavity during acute inflammation (4 h) (Figure 7F). This prompted us to speculate that additional anti‐inflammatory mechanisms may be involved, and we therefore extended our analyses to bioactive sphingolipids and endocannabinoids (Data Table S9).

Ceramides (Cer), ceramide‐1‐phosphate (C1P), and sphingosine‐1‐phosphate (d18:1) (S1P) have pleiotropic functions in the regulation of immunity and inflammation [9, 109, 113]. By activating G‐protein‐coupled S1P receptors, S1P regulates Toll‐like receptor signaling, immune cell trafficking, and maturation, among numerous other pathways [9, 114, 115]. Its plasma concentrations decrease in zymosan‐challenged mice [108] and negatively correlate with peritonitis in humans [116]. Notably, the contribution of S1P receptors to inflammation is complex, encompassing both anti‐ and pro‐inflammatory activities depending on the disease and experimental setting [9, 109, 117, 118, 119].

Compounds **2** and **3** profoundly affect sphingolipid metabolism and substantially elevate plasma S1P levels in mice during the resolution phase (18 h) and, in the case of **2**, also during the acute phase (4 h) (Figure 7G). Profiling of metabolic intermediates implies that **2** and **3** impair S1P degradation rather than enhance its biosynthesis, as plasma levels of its immediate precursor, sphingosine (So), are not elevated (while they are, interestingly, increased in the exudate) (Figure S20). Together, these data suggest that **2** and **3** counteract the depletion of S1P in peritonitis, however, it remains unclear, whether the elevated S1P levels result from a direct effect on sphingolipid turnover or indirectly from amelioration of the inflammatory state.

Endocannabinoids are potent immunomodulatory mediators that bind to cannabinoid (CB_1_ and/or CB_2_) and other receptors as extracellular ligands, while also triggering intracellular signaling cascades [11]. Agonists of these receptors have been shown to limit peritoneal inflammation in mice by blocking neutrophil infiltration into the peritoneal cavity [112] and stimulating LXA_4_ biosynthesis [110, 111]. Compounds **2** and **3** strongly increase endocannabinoid levels (2‐AG > PEA > OEA) during acute inflammation (4 h), specifically in the peritoneal exudate (Figure 7E,H; Figures S18E,F and S19). While endocannabinoid levels are maintained up to 18 h after administration of **2**, they return to baseline in mice treated with **3**, accompanied by a trend towards higher plasma concentrations. We conclude that **2** and **3** induce comprehensive changes in the cytokine and lipid mediator profile over the course of resolving inflammation, with favorable adaptations predominating, and that endocannabinoid accumulation is particularly prominent. Supporting the important role of endocannabinoids, the number of peritoneal infiltrating cells negatively correlated with local endocannabinoid concentrations in **3**, but not **2**‐treated mice (Figure 7I and Figure S21).

### 2.9 Shift Toward Anti‐Inflammatory and Anti‐Fibrotic Lipid Mediators in Murine Pulmonary Fibrosis

The chemotherapeutic agent bleomycin induces pulmonary toxicity, leading to interstitial edema accompanied by infiltration of immune cells, including neutrophils, macrophages, lymphocytes, and mast cells [120]. Activation of these cells by damage‐associated molecular patterns released from necrotic lung tissue amplifies the (necro)inflammatory response through the release of cytokines, lipid mediators, and reactive oxygen species [120, 121, 122]. This initial inflammatory phase subsequently progresses to pulmonary fibrosis, characterized by excessive production and deposition of extracellular matrix components, including collagen [123]. Several lipid mediators have been implicated in promoting pulmonary fibrosis (e.g., PGF_2α_, leukotrienes, AEA), whereas others exert antifibrotic actions or maintain lung function (PGE_2_, PGD_2_, EETs, certain endocannabinoids, and specific SPMs) [122, 124, 125, 126, 127, 128, 129]. For others, such as AEA, the direction of their effect remains controversially or context‐dependent [125, 128, 129].

To investigate how the meroterpenoids influence pulmonary and systemic lipid mediator profiles in fibrosis, mice were repeatedly exposed to bleomycin three times a week for two weeks, accompanied by regular administration of **3** (10 mg/kg, i.p., 1 h before bleomycin; Figure 8A). Under these experimental conditions, the acute inflammatory reaction had already subsided and transitioned into a fibrotic state characterized by low‐grade, persistent inflammation [130, 131]. As expected, bleomycin had only minor effects on lipid mediator profiles at this stage. The most prominent trends were an increase in PTGS, ALOX5, and ALOX12/15 product levels, accompanied by a decrease in pulmonary endocannabinoid levels (Figure 8B–D; Data Table S10). Co‐treatment with meroterpenoid **3** markedly elevated the levels of a subset of eicosapentaenoic acid (EPA)‐derived epoxy fatty acids (EpETEs) with reported anti‐inflammatory activity [132] and restored endocannabinoid levels to those of healthy controls (Figure 8C,D). In addition, compound **3** showed a trend toward increased pulmonary PGE_2_ levels (Figure 8C), which are generally considered anti‐fibrotic and contribute to the maintenance of lung function [122], as well as a trend toward elevated ALOX5 and ALOX12/15 product levels. The latter serve as precursors of SPMs, which, however, were not detected (Figure 8B). Systemic lipid mediator levels in plasma were only marginally affected by **3**, showing merely trends toward an overall decrease (Figure S22; Data Table S10).

**FIGURE 8:**
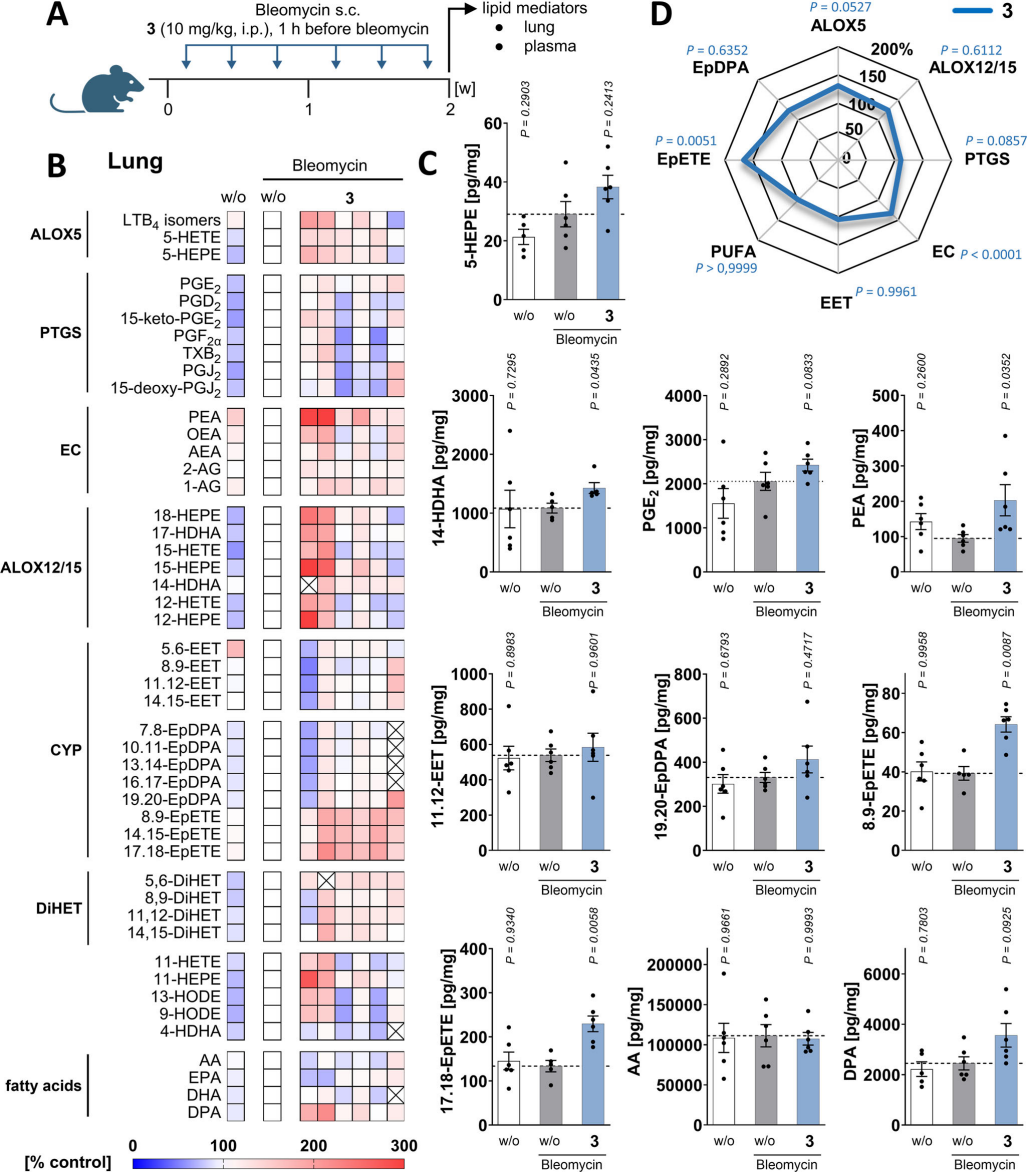
Meroterpenoids redirect the lipid mediator profile towards fatty acid epoxides, endocannabinoids, and PGE_2_ in murine bleomycin‐induced fibrosis. (A) Time course and experimental readouts for bleomycin‐induced fibrosis in mice. (B–D) Mice received vehicle (1% DMSO in saline; 0.5 mL) or 3 (10 mg/kg, i.p.) 1 h before bleomycin or mock injection three times a week over 2 weeks. Mice were sacrificed within 48 h after the last administration, and the lipid mediator profile of lung tissue was analyzed. (B) The heatmap depicts percentage changes in lipid mediator concentrations relative to vehicle control; crossed sections indicate significant outliers. (C) Levels of 5‐HEPE, 14‐HDHA, PGE_2_, PEA, 11,12‐EET, 19,20‐EpDPA, 8,9‐EpETE, 17,18‐EpETE, AA/20:4, and DPA/22:5 [per mg lung tissue]. (D) Percentage changes in the concentrations of ALOX5 products, ALOX12/15 products, PTGS products, endocannabinoids (EC), EETs, EpETEs, EpDPAs, and PUFAs. Mean (B‐w/o and w/o + Bleomycin, (D), individual values (B‐compound **3**), or mean + SEM and single data (C) from n = 5–6 mice. *P* values given vs. bleomycin‐treated vehicle control (C, D); ordinary one‐way ANOVA of log data + Dunnett *post hoc* tests. The lipid mediators belonging to each subclass are defined in panel A.

Together, meroterpenoid **3** redirects lipid mediator synthesis in fibrotic lung tissue toward anti‐inflammatory and anti‐fibrotic species.

### 2.10 Upregulation of Endocannabinoids via Inhibition of MGLL and Other Mechanisms

Endocannabinoids are classified into *N*‐acylethanolamines (AEA, PEA, and OEA), which are hydrolyzed and thereby inactivated by fatty acid amide hydrolase (FAAH), and monoacylglycerols (1‐AG and 2‐AG), which are degraded by monoacylglycerol lipase (MGLL, MAGL) [11]. Compound **3** and to a lesser extent **2** (10 > 1 µM) decreased cell‐free MGLL activity (Figure 9A), although the effect was moderate. To assess the functional relevance of this inhibition, we silenced MGLL in human MCF‐7 breast cancer cells (Figure 9B and Figure S23). Consistent with our findings in murine peritonitis, compound **3** (10 µM) increased 1‐ and 2‐AG levels in SACM‐stimulated, control‐transfected MCF‐7 cells, and *MGLL* knockdown markedly elevated 1‐ and 2‐AG levels, as expected (Figure 9C). Treatment with **3** did not further increase 1‐ and 2‐AG levels in *MGLL*‐silenced cells, confirming MGLL as a functionally relevant target of the meroterpenoid. Neither **2** nor **3** (up to 10 µM) inhibited human recombinant FAAH activity (Figure 9A).

**FIGURE 9:**
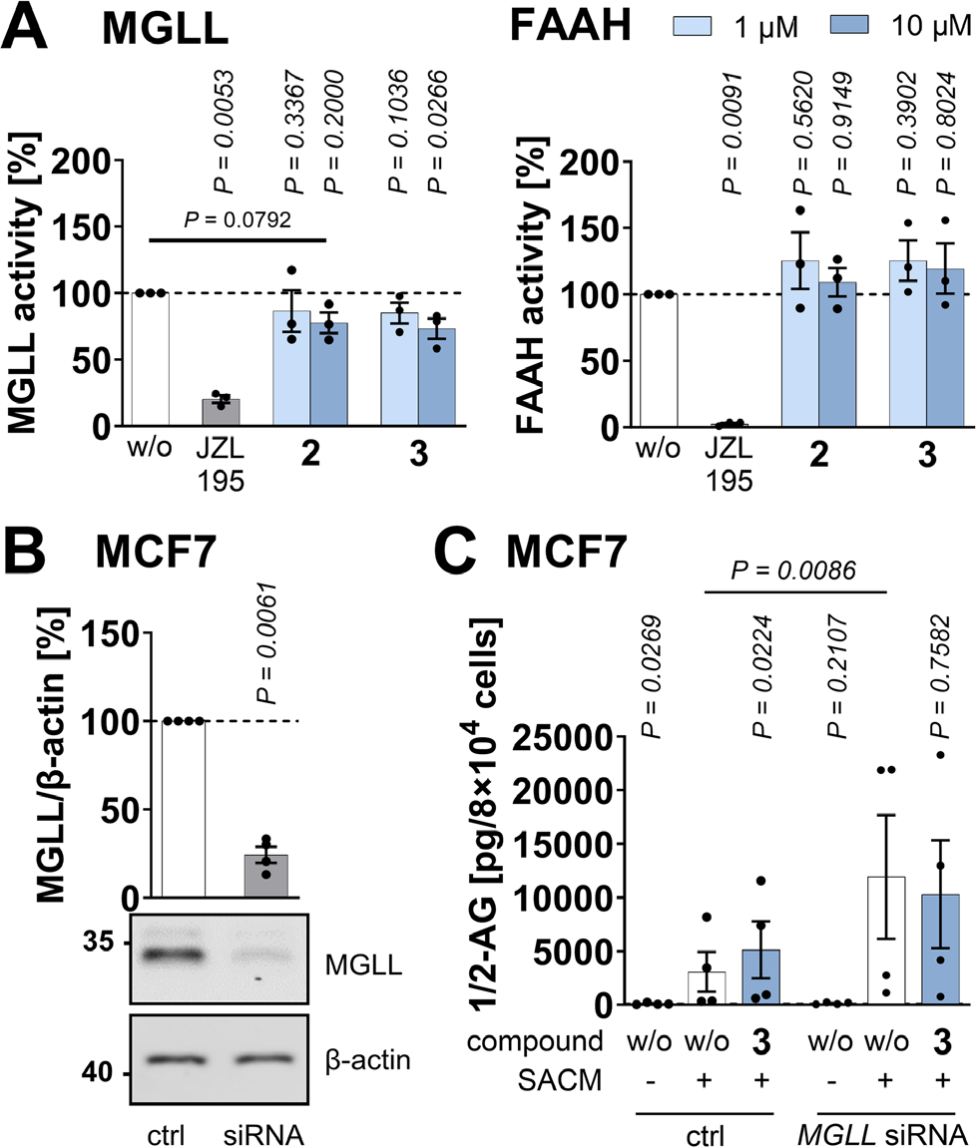
Meroterpenoid 3 increases endocannabinoid levels by inhibiting MGLL. (A) Human recombinant FAAH and MGLL were pretreated (FAAH: 5 min; MGLL: 15 min) with vehicle (DMSO, 0.1%), JZL195 (FAAH: 1 µM; MGLL: 4.4 µM), or test compounds before enzymatic activity was assessed via substrate hydrolysis yielding fluorescent or chromogenic products. (B, C) MCF‐7 cells transfected with control siRNA or *MGLL* siRNA. (B) MGLL protein expression; Western blots are representative of n = 4 independent experiments. Uncropped blots are shown in Figure S23. (C) Levels of 1‐ and 2‐AG after pre‐treatment with vehicle (DMSO, 1%) or compound **3** (10 µM), with or without SACM stimulation [per initially seeded 8 × 10^4^ cells]. Mean + SEM and single data from n = 3 (A), n = 4 (B,C) independent experiments. *P* values given vs. vehicle control (A), control‐transfected cells (B), or vehicle‐ and SACM‐treated control‐transfected or *MGLL* siRNA‐transfected cells, respectively (C); repeated measures one‐way ANOVA of log data + Dunnett *post hoc* tests (A, C) and two‐tailed paired Student *t* test of log data for pairwise comparisons as indicated by bars (A, C).

Overall, the limited inhibition of endocannabinoid‐degrading enzymes suggests that additional mechanisms underlie the strong increase in endocannabinoid levels, potentially involving altered lipid dynamics that provide phospholipid or diacylglycerol (DG) substrates for endocannabinoid biosynthesis.

### 2.11 The Meroterpenoids 1, 3, 4, and 7 Alter the Lipidome of Innate Immune Cells

The differential effects of meroterpenoids on the lipid mediator profile suggested the existence of another layer of regulation linked to cellular lipid composition. To investigate this, PBMCs were treated with meroterpenoids (1 and 10 µM) for 48 h and their lipid composition was analyzed, focusing on glycerophospholipids, sphingolipids, and neutral lipids, encompassing a total of 202 species (Data Table S11). Principal component analysis revealed three distinct clusters based on absolute differences in lipid composition (Figure 10A): Cluster B, comprising **1**, **3**, and **4**, together with compound **7** (cluster C), is clearly separated from Cluster A, which included vehicle control and meroterpenoids (**2**, **5**, **6**, **8**, **9**) with weaker effects on the overall lipidome (Figure 10B). Notably, compounds in cluster B induced a shift from neutral lipids, triacylglycerols (TGs) and/or cholesteryl esters (CE), toward phospholipids, specifically (lyso)phosphatidylcholine ((L)PC), phosphatidylserine (PS), cardiolipin (CL), and dihydrosphingomyelin (dhSM) (Figure 10B and Figures S24 and S25; Data Table S11). The reduction in neutral lipids, despite their role as core components of lipid droplets [133], does not seem to reflect impaired lipid droplet biogenesis, as treatment with **3** (10 µM) did not decrease the immunofluorescence signal of the lipid droplet‐coating protein perilipin‐2 in A23187‐activated PBMCs (Figure 12B,C and Figure S32). Absolute amounts of lipid subclasses are provided in Figures S26 and S27.

**FIGURE 10:**
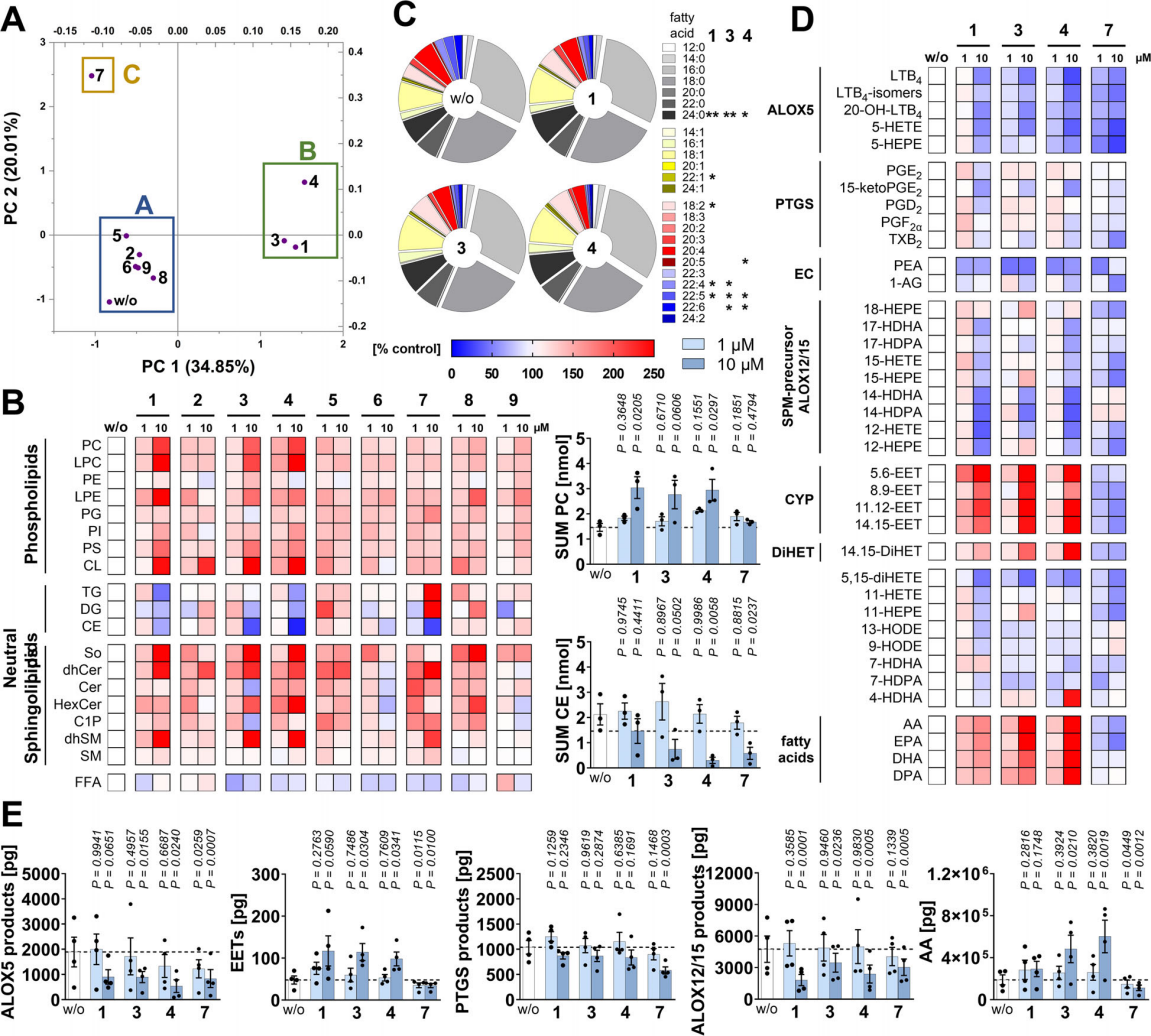
Prolonged exposure of PBMCs to meroterpenoids rearranges the cellular lipidome and establishes an anti‐inflammatory lipid mediator profile. (A–C) PBMCs were incubated with vehicle (DMSO, 0.1%) or test compounds (10 µM unless otherwise indicated) for 48 h to assess lipidomic changes. A) Principal component analysis of mean percentage changes in the amount of lipid species relative to vehicle control. (B) The heatmap depicts percentage changes in the abundance of individual lipid classes and bar charts showing total PC and CE levels [per 1 × 10^6^ PBMCs]. (C) Pie charts illustrating changes in the fatty acid composition across all 202 quantified lipid species. Detailed results for individual lipid species are provided in Figures S24 and S25. Lipid classes analyzed: phosphatidylcholine (PC), lysophosphatidylcholine (LPC), phosphatidylethanolamine (PE), lysophosphatidylethanolamine (LPE), phosphatidylglycerol (PG), phosphatidylinositol (PI), phosphatidylserine (PS), cardiolipin (CL), triacylglycerol (TG), diacylglycerol (DG), cholesteryl ester (CE), sphingosine (So), (dihydro)ceramide ((dh)Cer), hexosylceramide (HexCer), ceramide‐1‐phosphate (C1P), (dihydro)sphingomyelin ((dh)SM), and free fatty acids (FFA). D‐E) For lipid mediator analysis, PBMCs were pretreated (48 h) with vehicle (DMSO, 0.1%) or test compounds, washed, and activated with A23187 (10 min). (D) The heatmap depicts percentage changes in the lipid mediator levels relative to vehicle control. (E) Amounts of ALOX5 products, EETs, PTGS products, ALOX12/15 products, and AA/20:4 [per 1 × 10^6^ cells]. Mean (A–D) or mean + SEM and single data (B,E) from n = 3 (A–C), n = 3–4 (D), n = 4 (E) independent experiments. *P* values given vs. vehicle control (B, C, E) with *p < 0.05, **p < 0.01 (C); two‐tailed paired Student *t* test (C) or repeated measures one‐way ANOVA of log data (B, E) + Dunnett *post hoc* tests.

In contrast to the cluster B compounds, **7** (10 µM) suppressed CE but increased TG levels and exerted only mild effects on glycerophospholipids (Figure 10B; Figures S24–S27). The reduction in CE levels appears to be independent of *de novo* cholesterol biosynthesis, as cholesterol levels remained unchanged (Figure S26). Beyond altering total lipid subclass abundancies, **1**, **3**, **4**, and **7** (10 µM) profoundly reshaped the relative composition of esterified fatty acids (Figure 10C; Figures S28 andS29). Notably, significant decreases were observed for docosapentaenoic acid (22:5) and docosahexaenoic acid (22:6) (as well as other very long‐chain fatty acids), which constitute an important reservoir for anti‐inflammatory and pro‐resolving lipid mediator biosynthesis [6] (including Rvs, PDs, and MaRs). Their decline suggests enhanced mobilization or release from esterified pools. Taken together, distinct meroterpenoids have marked effects on both the abundance and fatty acid composition of major lipid classes in innate immune cells, with subtle structural variations, such as the methoxylation of **1** to **2**, critically determining their lipidome‐modulating activity.

### 2.12 Long‐Term Treatment of PBMC Favors the Production of EETs Over LTs

To assess how the lipidomic alterations induced by **1**, **3**, **4**, and **7** affect lipid mediator profiles, we investigated the capacity of reprogrammed PBMCs to generate lipid mediators. PBMCs were treated with the compounds (1 and 10 µM) for 48 h, washed, and subsequently stimulated with the Ca^2+^ ionophore A23187 to induce robust lipid mediator production (Data Table S12). As observed under short‐term conditions, **1**, **3**, and **4** (10> 1 µM), but not **7**, induced a lipid mediator class switch. However, in this long‐term setting, the switch occurred from ALOX5 products toward EETs, without elevating the levels of ALOX12/15 products, PTGS products, or endocannabinoids. In contrast, **7** (10 µM) exclusively suppressed the formation of ALOX5 and PTGS products (Figure 10D,E and Figure S30). SPMs were not detected under these stimulatory conditions. Although a redirection of AA/20:4 toward EET biosynthesis cannot be entirely ruled out for **3** and **4**, compound **1** (up to 10 µM) does not substantially increase free AA/20:4 levels (Figure 10E).

### 2.13 Correlation Between Neutral Lipid Availability and Lipid Mediator Production

To determine whether changes in the phospholipid or neutral lipid composition correlate with the shift from ALOX5 product to EET biosynthesis, PBMCs were incubated with **1**, **3**, **4**, or **7** (1 and 10 µM) for 48 h, followed by lipid composition analysis. Cells were then treated with A23187 in the absence of meroterpenoids to induce lipid mediator formation. The network shown in Figure 11A illustrates the co‐regulation of lipid species (nodes), with lipid classes distinguished by color. Node diameter reflects the relative proportion within each lipid class, and node shape indicates the fatty acid composition. Lipid species that are positively correlated cluster together and are connected by edges, whereas negatively or non‐correlated species are spatially separated.

**FIGURE 11:**
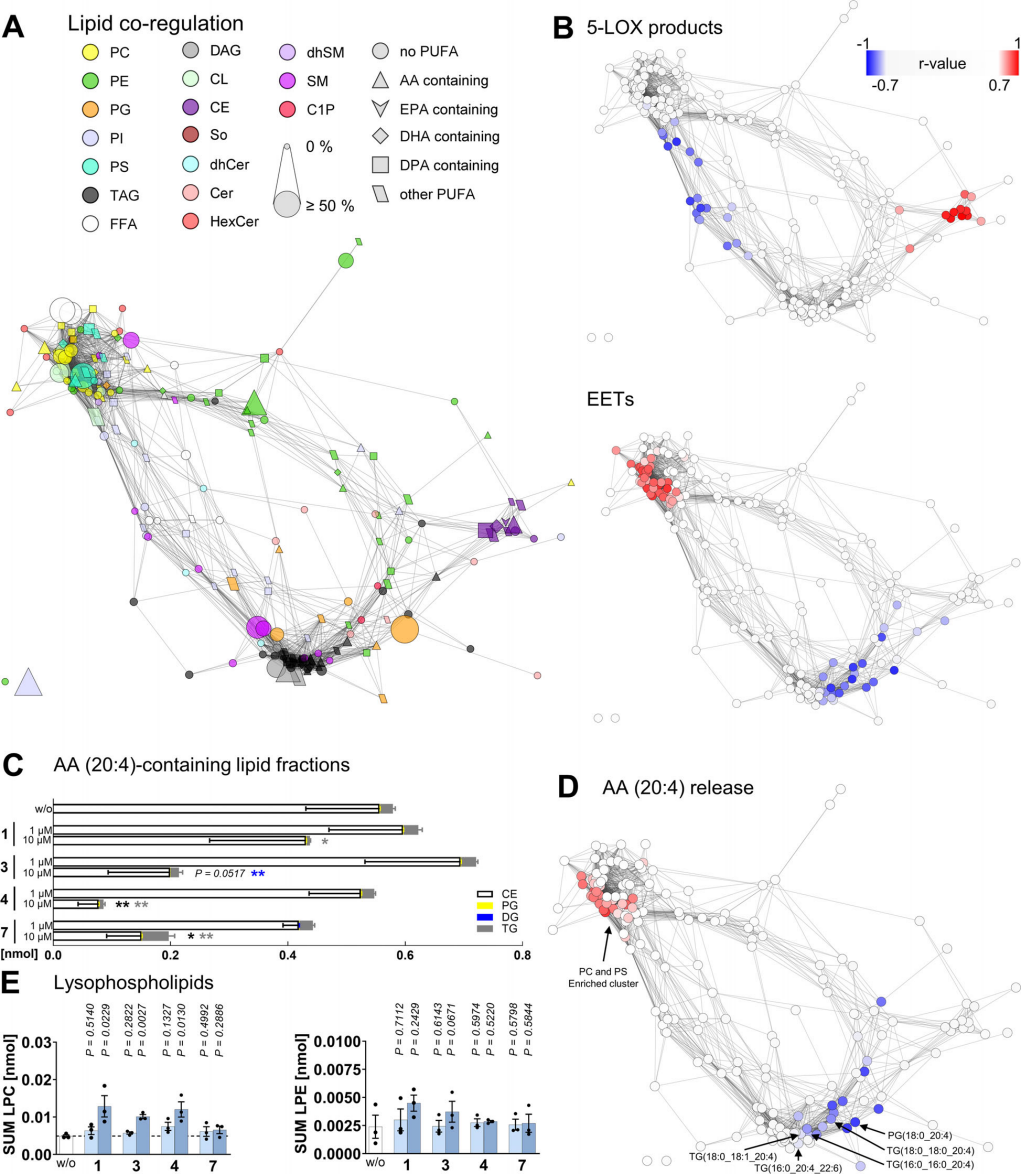
Depletion of neutral lipid fractions in PBMCs is associated with anti‐inflammatory changes in lipid mediator production. (A) Co‐regulated lipid network based on Pearson correlations depicting positive lipid–lipid correlations (r ≥ 0.7). Each node represents a specific lipid (for details see Figures S24 and S25) and indicates lipid class and fatty acid composition by color and shape, respectively. The network integrates data from PBMCs incubated with **1**, **3**, **4**, or **7** (1 and 10 µM each) for 48 h and was calculated from mean percentage changes in lipid amounts relative to vehicle from n = 3 independent experiments. (B) The network shown in A was overlaid with a color map indicating positive (r ≥ 0.7; red) and negative (r ≤ ‐0.7; blue) Pearson correlations of individual lipid species with lipid mediator subclasses produced by PBMCs preincubated with **1**, **3**, **4**, or **7** (1 and 10 µM each) for 48 h. followed by washing and treatment with calcium‐ionophore (A23187). The lipid mediators assigned to each subclasses are shown in Figure 10D. Correlations were calculated from mean percentage changes in lipid and lipid mediator quantities relative to vehicle control of n = 3 (lipids) or n = 4 (lipid mediators) independent experiments. (C) Amounts of AA/20:4‐containing TG, DG, CE, and PG species [per 1 × 10^6^ PBMCs]. (D) The network shown in Figure 11A was overlaid with a color map indicating positive (r ≥ 0.7) and negative (r ≤ ‐0.7) Pearson correlations of individual lipid species with AA/20:4 levels. The experimental procedure is identical to B. (E) Amounts of lysophospholipids in PBMCs incubated with vehicle (DMSO, 0.1%), **1**, **3**, **4**, or **7** for 48 h [per 1 × 10^6^ cells]. Mean (A, B, D) or mean + SEM (C) and single data (E) from n = 3 (A, C, E), n = 3–4 (B, D) independent experiments. *P* values given vs. vehicle control (C, E) with *p < 0.05, **p < 0.01 (C); repeated measures one‐way ANOVA of log data (C, E) + Dunnett *post hoc* tests.

In Figure 11B, colors indicate correlations between cellular lipid species and the amounts of ALOX5 products or EETs generated (positive: 0.7 ≤ *r* ≤ 1; negative: ‐1 ≤ *r* ≤ ‐0.7). Decreased ALOX5 product levels (Figure 10E) correlate positively with CE (including CE 20:4) and partially with TG species (e.g., TG(18:0_18:0_20:4)), whose levels were reduced by treatment with **1**, **3**, or **4** (Figure S25). EETs (increased levels; Figure 10E) exhibit the strongest negative correlations with TGs, as well as with phospholipids and Cer associated with the TG and CE clusters. Given that TGs and CE contain substantial amounts of AA/20:4 (Figure 11C), it is tempting to speculate that the shift from ALOX5 products to EETs upon treatment with **1**, **3**, or **4** (Figure 10D,E) is at least partially attributable to AA/20:4 supplied by these lipid subclasses. Additional positive and negative correlations are evident between the formation of ALOX5 products or EETs and the cellular lipid composition (Figure 11B). For instance, ALOX5 product formation negatively correlates with several PI and sphingolipid species, whereas EETs correlate positively with a compact phospholipid cluster comprising PC, PE, PS, and CL species.

The ALOX5 substrate AA is generally thought to be supplied mainly by phospholipases A_2_, which cleave phospholipids into free fatty acids (FFA) and lysophospholipids [26], although TG lipolysis also represents a significant source [134]. Our data indicate that the levels of AA released from activated PBMCs correlate positively with the levels of AA/20:4‐containing PC and PS (Figure 11D) and negatively with the levels of AA/20:4‐containing neutral lipids (TG and CE). Specifically, **3** and **4**, and to a lesser extent **1** (10 µM), substantially increased the levels of free AA in challenged cells (Figure 10E), which in turn, exhibited higher abundance of AA/20:4‐containing PC and PS and lower abundance of AA/20:4‐containing TG and CE after exposure to these meroterpenoids (Figure 10B and Figures S24–S27). These findings suggest that PBMCs reorganize AA/20:4 pools from neutral lipids toward phospholipids in response to meroterpenoids. As expected from such phospholipid turnover, **1**, **3** and **4** (10 µM) resulted in elevated levels of intracellular lysophospholipids (Figure 11E). We speculate that this rearrangement increases the phospholipase‐dependent release of AA/20:4 while shifting AA/20:4 sub‐pools in a manner that favors EET over ALOX5 product biosynthesis. In conclusion, long‐term incubation with **1**, **3**, and **4** induces remodeling of neutral lipids and phospholipids that increases the availability of free AA/20:4 and reprograms PBMCs from LT to EET biosynthesis.

### 2.14 Depletion of Neutral Lipids Impairs the Capacity for ALOX5 Product Formation

We investigated whether the decrease in TG and CE levels observed after long‐term treatment with **1**, **3**, and **4** drives the associated lipid mediator class switch from ALOX5 products to EETs in A23187‐activated PBMCs. To this end, we suppressed CE biosynthesis with the selective sterol‐*O*‐acyltransferase (SOAT) inhibitor TMP‐153 [135] or interfered with TG biosynthesis by inhibiting the diacylglycerol acyltransferase (DGAT)1 and 2 isoenzymes with A‐922500 [136] and PF‐06424439 [137], respectively. Membrane integrity and the number of viable cells were not compromised under these experimental conditions (Figure S31A). As expected, SOAT inhibition reduced cellular CE levels and DGAT‐1/2 inhibition lowered the TG content within 48 h; combined inhibition reduced both TG and CE amounts (Figure 12A; Data Table S13) as well as the number, but not size, of lipid droplets, as shown by perilipin‐2 staining (Figure 12B,C; Figures S31B and S32).

**FIGURE 12:**
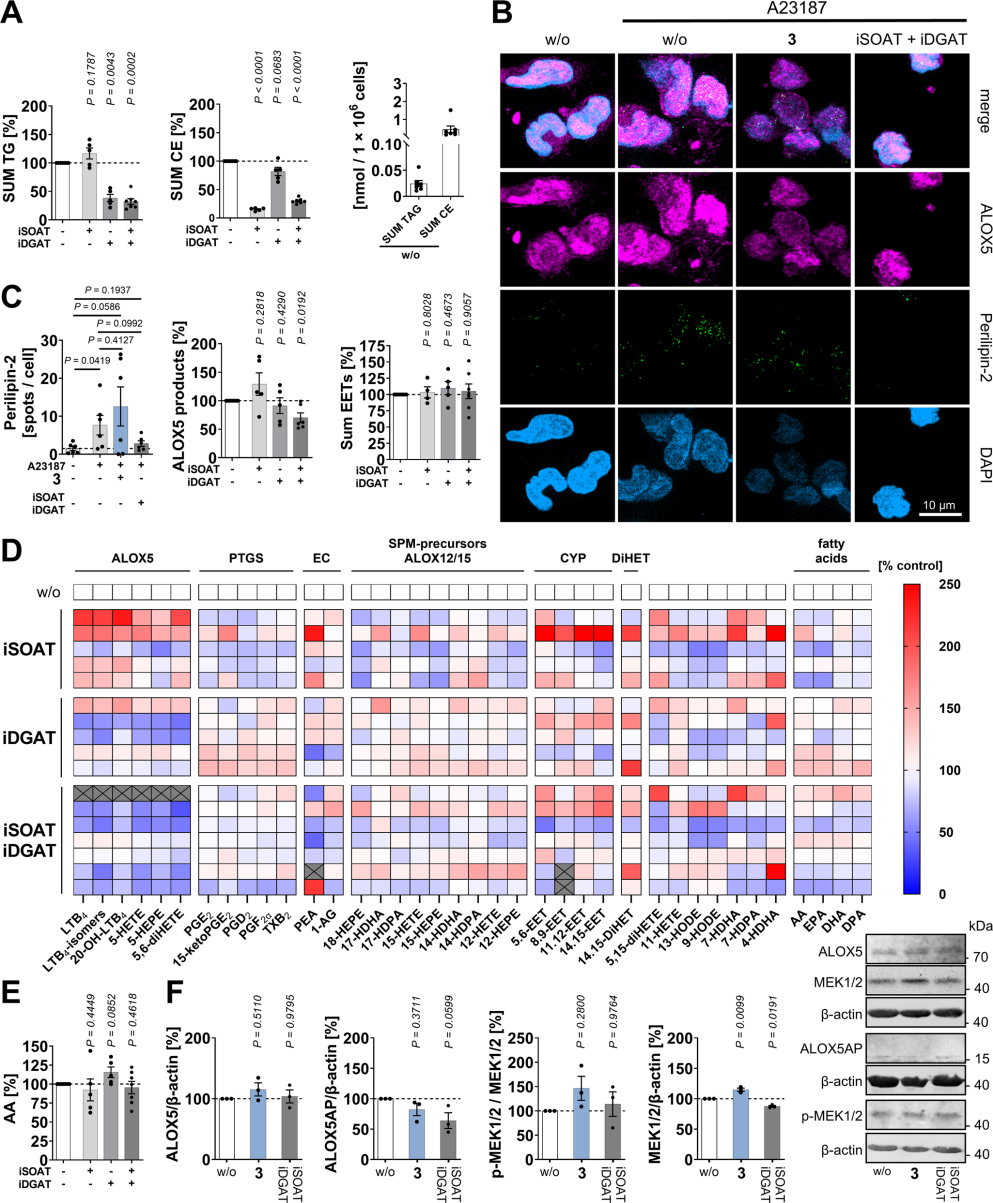
Combined SOAT and DGAT1/2 inhibition decreases neutral lipid levels and lipid droplet number and suppresses ALOX5 product formation in PBMCs. Cells were treated with vehicle (DMSO, 0.1%), **3** (10 µM), TMP‐153 (iSOAT; 500 nM), A‐922500 (10 nM) + PF‐06424439 (20 nM) (iDGAT), or TMP153 (100 nM) + A‐922500 (10 nM) + PF‐06424439 (20 nM) (iSOAT + iDGAT) for 48 h. (A) Changes in TG and CE contents [per 1×10^6^ PBMCs] and comparison of TG and CE concentrations. (B, C) For immunostaining, PBMCs were preincubated with vehicle (DMSO, 0.1%), **3** (10 µM), or iSOAT + iDGAT for 48 h, the medium was exchanged, and cells were activated with calcium ionophore (A23187, 5 min). Immunofluorescence images show perilipin‐2 (green, exposure time 35 ms), ALOX5 (violet, exposure time 200 ms), and nuclei (DAPI, light blue, exposure time 10 ms). Images are representative of ≥ 70 single cells analyzed in n = 3 independent experiments. (C) Number of perilipin‐2‐stained spots normalized to cell number. Zoomed images used for quantitation are shown in Figure S32. (C–E) For lipid mediator analysis, PBMCs were pretreated with vehicle (DMSO, 0.1%), iSOAT, iDGAT, or iSOAT + iDGAT for 48 h, washed, and activated with calcium ionophore (A23187, 10 min). (D) The heatmap depicts percentage changes in lipid mediator levels relative to vehicle control for each independent experiment; crossed sections indicate significant outliers. (E) Free AA/20:4 levels. F) Effect of **3** and iSOAT + iDGAT on the expression of ALOX5, ALOX5AP, and MEK1/2 and on MEK1/2 phosphorylation. Western blots are representative of n = 3 independent experiments. Uncropped blots are shown in Figure S34B. Mean (D) or mean + SEM and single data (A, C, E, F) from n = 3 (B,C perilipin‐2,F), n = 4–7 (A,C,D,E) independent experiments. *P* values given vs. vehicle control (A,C,E,F) or as indicated by bars (C); repeated measures one‐way ANOVA + Dunnett *post hoc* tests (F), two‐tailed paired Student *t* test of log data (A,C,E) or two‐tailed unpaired Student *t* test (C perilipin‐2).

Interestingly, only the combined treatment with SOAT and DGAT1/2 inhibitors markedly attenuated ALOX5 product formation in A23187‐activated PBMCs (Figure 12C,D) without obviously altering the subcellular localization of ALOX5 (Figure S33), whereas single treatments had no effect and EET levels were only marginally increased (Figure 12C). Inhibition of both SOAT and DGAT1/2 did not markedly change the levels of free AA/20:4 (Figure 12E). As observed for **3** (10 µM), dual inhibition did not substantially affect ALOX5 expression (Figure 12F) or its localization (Figure S33), nor did it alter mitogen‐activated protein kinase kinase (MEK)1/2 phosphorylation (Figure 12F and Figure S34A), which precedes ALOX5 nuclear translocation [14, 138].

However, SOAT together with DGAT1/2 inhibition tended to reduce the overall very low levels of ALOX5AP expression, which may contribute to the decrease in ALOX5 product formation (Figure 12F). Additionally, MEK1/2 expression was slightly reduced by dual SOAT and DGAT1/2 inhibition, whereas **3** (10 µM) increased MEK1/2 expression, resulting in elevated cellular p‐MEK1/2 levels (Figure 12F and Figure S34A), possibly reflecting a compensatory response to the strong inhibition of ALOX5.

Together, our data indicate that limiting the availability of neutral lipids (TGs and CE), as achieved by dual SOAT and DGAT1/2 inhibition and also observed for **3** and related meroterpenoids, suppresses ALOX5 product formation in innate immune cells. However, additional mechanisms likely contribute to the reduced capacity for ALOX5 product formation, given the superior potency of **3** (Figure 10E) compared to SOAT and DGAT1/2 inhibitors (Figure 12C), even when the meroterpenoid was removed prior to cell activation. Impaired ALOX5AP expression can be excluded as a major driver (Figure 12F), since its reduction was more pronounced under combined SOAT and DGAT1/2 inhibition than upon treatment with **3**.

## 3 Discussion

The epilipidome comprises a structurally and functionally diverse set of lipids, including oxidized species, whose physiological functions at a systems level are only beginning to be understood [139]. Lipid mediators such as prostanoids, LTs, and EETs were early recognized as key regulators of inflammatory processes, but also exert important functions beyond inflammation [3, 12, 15, 16]. Accordingly, major efforts have been devoted to the development of anti‐inflammatory drug candidates targeting central enzymes involved in lipid mediator biosynthesis or degradation, including PTGS, ALOX5, ALOX5AP, microsomal prostaglandin E_2_ synthase, and soluble epoxide hydrolase [3, 48, 58, 140, 141], and polypharmacological ligands addressing multiple nodes of these pathways have been successfully designed [16, 56, 57, 77]. However, with the steadily expanding number of bioactive lipid species and the emerging functional links between lipid mediators, cellular metabolism, and lipid pools that were long considered merely structural, lipid‐centered drug development faces increasing complexity. Next‐generation therapeutic strategies will therefore require the integration of OMICs technologies with network‐based approaches to define disease‐associated lipid signatures and to discover small molecules that enable a targeted manipulation of local and systemic lipid profiles. This is particularly relevant given that lipid networks are highly dynamic and kinetically regulated, responding to both intrinsic and extrinsic cues, including the metabolic status of cells and organisms [142, 143, 144]. In line with this, it is now widely accepted that dysregulated cellular metabolism is a major driver of inflammation, and that a deeper understanding of this underexplored interplay may open new avenues toward more effective and safer anti‐inflammatory therapies [145, 146, 147].

Based on these considerations, we initiated a drug discovery program aimed at identifying novel biogenic scaffolds capable of favorably manipulating defined lipid signatures in human innate immune cells. Our initial focus was on small molecules that suppress the biosynthesis of pro‐inflammatory lipid mediators while simultaneously increasing the levels of anti‐inflammatory or pro‐resolving lipid mediators. This concept was inspired by the current understanding of inflammation as a biphasic process comprising an initiation phase and an active resolution phase [1], the latter being largely governed by lipid mediators with pro‐resolving functions [5]. Although there is ongoing debate about whether SPMs, particularly tri‐hydroxylated species, are produced at concentrations sufficient to elicit physiological responses, and uncertainties remain regarding their involved receptors and biosynthetic pathways [40, 41, 42, 43, 44], there is strong evidence from animal studies that (supra)physiological doses of SPMs (e.g., upon injection) are highly effective in alleviating inflammation [5, 38]. Moreover, initial clinical trials on SPM analogs [148] or SPM precursors [149, 150] support their anti‐inflammatory and analgesic potential. Thus, the major challenge for small molecule inflammation‐resolving therapeutics is not only to elevate SPM levels, but to do so sufficiently to achieve robust and sustained biological effects.

Another intensively discussed aspect of SPM research concerns the technical requirements of high‐quality SPM analytics, as earlier methodological approaches have been criticized for not adhering to established bioanalytical conventions [41]. These controversies have prompted joint community efforts to develop technical recommendations for lipid mediator analysis by LC‐MS/MS [151]. Nevertheless, some ambiguity remains regarding the stereochemical resolution of SPMs analyzed on conventional reversed phase columns, which can only be addressed by chiral chromatographic separation [44]. Accordingly, while our analytical workflow is highly specific, discriminating lipid mediators based on molecular mass, retention time (defined by external standards), and diagnostic fragment ions, signals for certain SPMs may still include unresolved isomers. Additional resolving power provided by instrument‐integrated differential mobility separation could not be applied due to the low abundance of SPMs. This represents an inherent limitation of current state‐of‐the‐art SPM analytics, which also applies to the present study.

Following our established routine, we identified the meroterpenoid **1** as a candidate that potently suppresses ALOX5 product formation while increasing SPM/iso biosynthesis. Building on this initial hit, we first investigated whether structural modifications introduced by total synthesis [78] could further enhance this lipid mediator class switch. This approach successfully identified structural features that enabled derivatives to outperform the parental compound and to increase SPM/iso production in resting (unchallenged) cells, exceeding the effects of previously described (non‐toxic) small molecules known to upregulate SPM/iso biosynthesis [59, 60, 61, 62, 63, 64, 66, 67, 68, 69, 70, 71, 72, 73, 74]. Here, we deliberately compare fold‐increases rather than absolute SPM/iso concentrations across studies, as metabololipidomics still lacks sufficient inter‐laboratory standardization [152], leading to absolute values that can differ by more than an order of magnitude, even under comparable experimental conditions [42]. Second, we expanded our analysis of the lipid mediator network to additional anti‐inflammatory and immunomodulatory lipid classes, such as EETs, endocannabinoids, and bioactive sphingolipids. These lipid classes were likewise substantially modulated, predominantly showing increased levels, although the direction and extent of these changes differed between experimental systems and were, in part, opposite. For example, compound **3** markedly increased EET levels in SACM‐challenged human macrophages, whereas EET levels were decreased in peritoneal exudates from zymosan‐injected mice.

Meroterpenoids exhibit overall anti‐inflammatory activity, as demonstrated in a mouse model of self‐resolving inflammation, however it is difficult to disentangle the contribution of individual lipid changes, and certain adjustments may even be detrimental under specific disease conditions. While it is generally accepted that LTs promote [3, 14, 16, 29], EETs suppress [153], and SPMs resolve inflammation [38], the directionality of the effects is less clear for PGE_2_ [16, 27, 28], endocannabinoids [11], and S1P [49]. Although all three play central roles in immune regulation and have well‐documented anti‐inflammatory functions, they can also promote the inflammatory responses in a context‐dependent manner [9, 11, 16, 27, 28, 49, 154, 155]. This complexity of lipid mediator regulation reflects the multifaceted nature of inflammation and underscores the need to distinguish between inflammatory diseases when devising targeted therapeutic strategies.

It is remarkable that small structural modifications in meroterpenoids enable fine‐tuning of the lipid profiles generated by innate immune cells. This is particularly relevant in the context of SPMs, for which the (patho)physiological functions and signaling pathways of individual subclasses remain incompletely understood [2, 38, 40]. In this regard, such compounds may serve as valuable tools for the directional manipulation of endogenous SPM production. Specifically, we found that meroterpenoids shift SPM/iso production in activated M2‐like macrophages toward either PD/iso (**5**), PD/iso and MaR/iso (**2**), or all major SPM/iso subclasses (**3**). These preferences in shaping lipid mediator profiles do not readily correlate with discernible differences in molecular mechanism, at least based on our current data, and may instead reflect compound‐specific subcellular accumulation or differences in intracellular kinetics. Notably, SPM biosynthesis is a tightly coordinated and still incompletely understood process involving ALOX isoenzymes and other enzymes that reside in distinct subcellular compartments. For efficient SPM biosynthesis, the spatial reorganization and membrane association of some of these enzymes seems to be required, at least under physiological conditions [14, 33].

Unraveling the molecular mechanisms underlying such extensive lipid alterations is hampered by the multitude of possible targets and the still incomplete understanding of lipid mediator networks [3, 12, 13, 14, 33, 40, 153]. At this point, we can explain the suppression of leukotriene biosynthesis, interestingly achieved either by direct ALOX5 inhibition or by interference with ALOX5AP, and gained a basic understanding of the molecular requirements for the induction of SPM/iso biosynthesis. The latter seems to involve a moderate release of PUFAs from various sources, which promotes SPM/iso biosynthesis but is otherwise dispensable, and a facilitated translocation of ALOX15 to particulate cytoplasmic compartments. This phenomenon has been described previously and linked to efficient SPM/iso biosynthesis [33, 36, 86], but the exact nature of the involved subcellular structures and the functional significance of the mechanism remain undefined, as does the role of the ALOX15B isoenzyme, which is less abundantly expressed in M2‐like macrophages. Based on the meroterpenoid‐induced changes in the lipid mediator profile, specifically the increased formation of 14‐ and 17‐HDHA as well as HDPA, we consider ALOX15, rather than ALOX15B, as the regulated enzyme. Human ALOX15 preferentially catalyzes 15‐ over 12‐lipoxygenation of AA/20:4 and EPA/20:5 and exhibits comparable 14‐ and 17‐lipoxygenation activity for DHA, whereas ALOX15B is restricted to 15‐ or 17‐lipoxygenation [46, 156]. In line with this, the meroterpenoid‐induced increase in SPM/iso formation also extends to MaR2/iso—an SPM whose biosynthesis does not depend on 17‐lipoxygenation [157]. Whether other ALOX isoenzymes, such as serine 663‐phosphorylated ALOX5 [32], contribute to 15‐lipoxygenation in meroterpenoid‐treated innate immune cells remains unclear. However, we consider this mechanism unlikely, given the ALOX5‐inhibitory activity of some meroterpenoids and the inability of **3** to induce a shift in regiospecificity of non‐phosphorylated ALOX5. Together with other studies [68], our data support an essential role of ALOX15 in SPM/iso formation in activated M2‐like macrophages, although more detailed mechanistic studies on how meroterpenoids act on human ALOX15 are warranted. Such investigations are, however, limited by the requirement of human primary cells to achieve robust ALOX15 expression and activity, as these cells are largely incompatible with routine gene manipulation strategies. Moreover, murine model systems are poor substitutes, as mouse ALOX15 and ALOX15B orthologs primarily exhibit 12‐ and less 15‐dioxygenating activity [158].

Here, we show that meroterpenoids mobilize PUFAs from different lipid subclasses and, over longer time scales, favorably redirect AA/20:4 from neutral lipids to specific phospholipid classes. Less well understood are the subcellular compartments from which these PUFAs are derived and whether their mobilization from distinct lipid classes is functionally coupled to the biosynthesis of specific lipid mediator subclasses. Initial hints into this relationship emerged from our network correlation analyses based on PBMCs subjected to long‐term meroterpenoid treatment, focusing on derivatives that exert pronounced effects on the cellular lipid composition. Correlating this co‐regulated lipid network with the capacity of PBMCs to produce lipid mediators—measured after removal of the meroterpenoids to exclude direct effects on lipid biosynthetic enzymes—revealed unexpected associations between the levels of LT or EET and the abundance of major neutral and membrane lipid classes. These correlations were unexpected because they were not restricted to PUFA‐containing species but also involved TGs, CEs, and phospholipids carrying saturated or monounsaturated fatty acids.

We confirmed the functional relevance of neutral lipid availability for ALOX5 product formation by selectively inhibiting SOAT to suppress CE biosynthesis and DGAT1 and DGAT2 to suppress TG biosynthesis. Notably, only the combined inhibition of all three acyltransferases reduced the long‐term capacity of PBMCs to generate ALOX5 products, indicating that depletion of a single neutral lipid class is insufficient to impair LT biosynthesis. These findings may be of clinical relevance, as SOAT and DGAT inhibitors have yielded mixed outcomes in phase II and III clinical trials, and SOAT inhibitors are approved in Japan and other countries for the treatment of hypercholesterolemia and atherosclerosis [159, 160, 161], diseases with an inflammatory component [162, 163, 164] previously linked to LT biosynthesis [162].

Our data are consistent with earlier studies reporting reduced LT production upon manipulation of cellular TG and CE levels, either through DGAT1 inhibition [165, 166] or by limiting PUFA mobilization from lipid droplets via targeting adipose triglyceride lipase (ATGL) [167, 168] or lysosomal acid lipase (LAL) [169]. These enzymes, together with hormone‐sensitive lipase, are expressed in human PBMCs [170, 171, 172] and contribute to PUFA release from neutral lipids and phospholipids stored in lipid droplets [134], which have been identified as sites of LT formation [173, 174]. Lipid droplets are highly dynamic organelles that engage in extensive membrane contact with other cellular compartments and play a central role in lipid and energy homeostasis [133]. Importantly, lipid droplets host several enzymes in lipid mediator biosynthesis [134, 174], including ALOX5 [134], and are depleted upon combined SOAT and DGAT inhibition under our experimental conditions.

Taken together, these observations support the notion that depletion of neutral lipids within lipid droplets may either limit the availability of substrates for ALOX5 (and potentially other isoenzymes in lipid mediator biosynthesis) or perturb the subcellular distribution required for efficient functional coupling between ALOX5, ALOX5AP, and/or other lipoxygenases, thereby attenuating ALOX5 product formation.

## 4 Conclusion

Low‐grade inflammation is a central driver of chronic inflammatory and metabolic diseases and represents an important target for the development of efficient and safe therapeutic strategies. OMICs technologies combined with network science are opening new opportunities to identify small molecules capable of resetting aberrant metabolism on a systems level. By applying functional metabololipidomics and focusing on lipid mediator class switches in innate immune cells—shifts that are supposed to suppress or resolve inflammation and contribute to immunoregulation—we identified biogenic meroterpenoid scaffolds that favorably manipulate lipid mediator profiles through multiple mechanisms, both in innate immune cells in vitro and models of self‐resolving and two‐stage inflammation in vivo. The modes of action range from direct interference with overall pro‐inflammatory factors in lipid mediator metabolism (ALOX5, ALOX5AP, MGLL, p‐IκBα), to control of subcellular enzyme localization (ALOX15), regulation of PUFA availability, and long‐term reprogramming of the cellular lipid composition that governs the capacity for lipid mediator biosynthesis. Together, our findings highlight the potential of lipidomics‐guided drug discovery for identifying anti‐inflammatory small molecule leads and advance our understanding of the emerging interplay between lipid diversity, cellular metabolism, and low‐grade inflammation.

## 5 Experimental Section

### 5.1 Test Compounds, Lipids and Standards

Compounds **1** to **9** were synthesized as described, with their purity being determined by ^1^H‐NMR to be ≥ 95% [78]. Detailed characterization data and the corresponding spectra are available in the Supporting Information of Wildermuth et al., [78] and Haut et al., [175]. Test compounds and small molecule inhibitors were dissolved in DMSO and stored in the dark at ‐20°C (test compound working solutions and inhibitors) or ‐80°C (test compound stock solutions) under argon, minimizing freeze‐thaw cycles. Lipid mediators, fatty acids, and analytical standards were obtained from Cayman Chemical (Ann Arbor, MI) and stored at ‐80°C upon receipt. Lipids were diluted in methanol/water (1:1) (for fatty acids, oxylipins, and endocannabinoids) or methanol (for phospholipids and neutral lipids), and aliquots were stored in the dark at ‐80°C under argon.

### 5.2 Analysis of Cell Numbers, Viability, and Mitochondrial Dehydrogenase Activity

Membrane integrity as well as total and viable cell numbers were determined using a Vi‐CELL Series Cell Counter (Beckman Coulter, Krefeld, Germany; software: Vi‐Cell XR Cell Viability Analyzer, version 2.06.3) after trypan blue staining [25].

Cellular dehydrogenase activity was assessed based on the conversion of 3‐(4,5‐dimethylthiazol‐2‐yl)‐2,5‐diphenyltetrazolium bromide (MTT) (Sigma–Aldrich, St. Louis, MO) as described [64, 176]. Briefly, human PBMCs (2 × 10^5^/well in 96‐well plates) were treated with vehicle (DMSO, 0.5%), test compounds, or staurosporine (1 µM, pan‐kinase inhibitor, Sigma–Aldrich) and incubated for 24 or 48 h at 37°C and 5% CO_2_ in RPMI 1640 medium (100 µL) supplemented with 5% FCS (Thermo Fisher Scientific), 2 mmol/L *L*‐glutamine, 100 U/mL penicillin (Sigma‐Aldrich), and 100 µg/mL streptomycin (Sigma‐Aldrich). Subsequently, MTT (5 mg/mL in PBS pH 7.4; 20 µL) was added and cells were incubated for an additional 3 h (37°C; 5% CO_2_) until visible formation of blue formazan crystals. Formazan was solubilized by addition of SDS lysis buffer (10% SDS in 20 mM HCl; 100 µL) and incubation for > 16 h under gentle shacking. Absorbance was measured at 570 nm using a SpectraMax iD3 microplate reader (Molecular Devices, San José, CA).

### 5.3 Isolation of PBMCs From Human Blood Products

The Central Institute for Blood Transfusion and Immunology of the University Hospital Innsbruck, Tirol Kliniken GmbH (Austria), provided leukoreduction system chambers (LRSCs) derived from platelet donors (18–65 years). Leukocyte concentrates (buffy coats) from female and male blood donors (18–65 years), used in Figure 4C,E, and Figures S8, S9, S10, and S12, were obtained from the Clinical Department of Blood Group Serology and Transfusion Medicine, Medical University of Graz (Austria). Informed consent allowing the use of residual blood for scientific purposes was obtained from all donors. Platelet and blood donors were accepted based on a standardized health questionnaire and routine blood testing. All platelet and blood donors met the criteria of the Austrian Blood Donation Regulation (BGBI. II Nr. 217/2022).

Human PBMCs were freshly isolated from LRSCs and buffy coats as previously described [63, 91, 177]. Briefly, PBMCs were obtained by isopycnic density gradient centrifugation using Histopaque‐1077 (Sigma‐Aldrich, St. Louis, MO, USA) (400 × g, 20 min, room temperature (RT)), followed by hypotonic lysis of erythrocytes (water) and two consecutive washing steps with PBS pH 7.4 (270 × g, 5 min, RT).

### 5.4 Monocyte Differentiation and Polarization Into M1‐ and M2‐Like Macrophages

Freshly isolated PBMCs, consisting predominantly of CD14^+^/CD16^+^ and CD14^+^/CD16^−^ monocytes (LSRC: 46.4 ± 4.9%, buffy coats: 38.8 ± 3.6%, as determined by flow cytometric analysis), were differentiated into monocyte‐derived macrophages (M_0_) by stimulation with 20 ng/mL GM‐CSF (HiSS Diagnostics GmbH, Freiburg, Germany) or M‐CSF (HiSS Diagnostics GmbH or PeproTech, Hamburg, Germany) for 6 days in macrophage medium (RPMI 1640 supplemented with 10% FCS, 2 mmol/L *L*‐glutamine, 100 U/mL penicillin, and 100 µg/mL streptomycin).

For polarization, M_0_ macrophages were incubated for an additional 48 h in macrophage medium supplemented with 100 ng/mL lipopolysaccharide (LPS; *Escherichia coli* O127:B8, Sigma Aldrich) and 20 ng/mL interferon‐γ (PeproTech) to generate M1‐like macrophages, or with 20 ng/mL interleukin‐4 (PeproTech) to generate M2‐like macrophages [63, 64].

### 5.5 Treatment of Human Innate Immune Cells for Lipid Mediator Analysis

Human PBMCs (1 × 10^6^, 5 × 10^6^, or 1 × 10^7^ cells/mL in 1 mL PBS pH 7.4, supplemented with 1 mM CaCl_2_) were pretreated with vehicle (DMSO, 0.1%) or test compounds for 10 min at 37°C. Cells were then stimulated with the Ca^2+^ ionophore A23187 (Cayman Chemical; 2.5 µM, 10 min), either alone or in combination with AA/20:4 (Cayman Chemical; 20 µM) or SACM (1%, 180 min). Alternatively, PBMCs (1 × 10^7^ cells/mL) were pretreated in RPMI 1640 medium with vehicle (DMSO, 0.1%) or test compounds for 10 min at 37°C before and subsequently stimulated with LPS (0.1 µg/mL) or TNF‐α (PeproTech, 20 ng/mL) for 24 h. Monocyte‐derived M1‐ and M2‐like macrophages (2 mL PBS pH 7.4 supplemented with 1 mM CaCl_2_) were preincubated with vehicle (DMSO, 0.1%), BLX3887 (Cayman, 0.3 – 1 µM), and/or test compounds at 37°C and 5% CO_2_ for 15 min (or 15–45 min for BLX3887 (co‐)treatment) before cells were challenged with SACM (1.0%, 3 h). Alternatively, compounds were added during macrophage polarization for 48 h, after which cells were washed and stimulated with SACM as described above for PBMCs and macrophages.

SACM was prepared by culturing *Staphylococcus aureus* (LS1 strain) in Brain Heart Infusion (BHI) medium for 18 h, followed by centrifugation (3400 × g, 10 min, RT) and sterile filtration of the supernatant through a Rotilabo syringe filter (PVDF, 0.22 µm, Roth, Karlsruhe, Germany) [177, 178]. Alternatively, M2‐like macrophages were incubated with vehicle (DMSO, 0.1%) or test compounds for 195 min without additional stimulation to assess lipid mediator profiles under resting conditions.

Human PMNLs (5 × 10^6^ cells/mL in 1 mL PBS pH 7.4, supplemented with 1 mg/mL glucose and 1 mM CaCl_2_) were preincubated with vehicle (0.1% DMSO), zileuton (3 µM), or test compounds for 10 min and subsequently activated with A23187 (2.5 µM) and supplemented with AA/20:4, 20 µM) for 10 min. Alternatively, cells were primed with LPS (1 µg/mL) and adenosine deaminase (0.3 units; Sigma–Aldrich) for 10 min at 37°C, pretreated with vehicle (DMSO, 0.1%) or test compounds for 30 min, and subsequently stimulated with *N*‐formylmethionyl‐leucyl‐phenylalanine (fMLP, 1 µM) for 5 min.

To investigate long‐term effects on lipid mediator profiles, freshly isolated human PBMCs (1 × 10^7^ cells/mL in 4 mL RPMI 1640 medium supplemented with 5% FCS, 2 mM *L*‐glutamine, 100 U/mL penicillin, and 100 µg/mL streptomycin) were incubated for 48 h with vehicle (DMSO, 0.1%), test compounds, the SOAT inhibitor TMP‐153 (500 nM), the DGAT1 inhibitor A‐922500 (10 nM) plus the DGAT2 inhibitor PF‐06424439 (20 nM), or TMP153 (100 nM) in combination with A‐922500 (10 nM) and PF‐06424439 (20 nM). Cells were harvested by sequential washing with ice‐cold PBS pH 7.4 containing 5 mM EDTA (1 mL), followed by ice‐cold PBS pH 7.4 (1 mL). Viable cell counts were determined using a Vi‐CELL Series Cell Counter, as described above. Cells were pelleted (270 × g, 7 min, 4°C), washed with ice‐cold PBS pH 7.4 (1 mL), resuspended in ice‐cold PBS pH 7.4 containing 1 mM CaCl_2_ (1 mL), and stimulated with the Ca^2+^ ionophore A23187 (Cayman Chemical; 2.5 µM, 10 min) at 37°C.

Lipid mediator formation was stopped by adding ice‐cold methanol (2 mL for PBMCs; 3.5 mL for macrophages) containing deuterium‐labeled internal standards (Cayman Chemical, Ann Arbor, MI). The standard mixture included 200 pg each of 5*S*‐hydroxy‐6*E*,8*Z*,11*Z*,14*Z*‐eicosatetraenoic‐5,6,8,9,11,12,14,15‐d8 acid (d8‐5S‐HETE), 5*S*,12*R*‐dihydroxy‐6*Z*,8*E*,10*E*,14*Z*‐eicosatetraenoic‐6,7,14,15‐d4 acid (d4‐LTB_4_), 9‐oxo‐11α,15*S*‐dihydroxy‐prosta‐5*Z*,13*E*‐dien‐1‐oic‐3,3,4,4‐d4 acid (d4‐PGE_2_), 5*S*,6*R*,15*S*‐trihydroxyicosa‐7*E*,9*E*,11*Z*,13*E*‐tetraenoic‐19,19,20,20,20‐d5 acid (d5‐LXA_4_), and 7*S*,16*R*,17*S*‐trihydroxy‐4*Z*,8*E*,10*Z*,12*E*,14*E*,19*Z*‐21,21',22,22,22‐d5 docosahexaenoic acid (d5‐RvD_2_), as well as 2000 pg 5*Z*,8*Z*,11*Z*,14*Z*‐eicosatetraenoic‐5,6,8,9,11,12,14,15‐d8 acid (d8‐AA). For Figures 7B–C,E,H,I, 10D,E, 11B,D, and 12C–E; Figures S18B–F, S19, and S30, the mixture was supplemented with 200 pg (±)8(9)‐epoxy‐5*Z*,8*Z*,14*Z*‐eicosatrienoic‐16,16,17,17,18,18,19,19,20,20,20‐d11 acid (d11‐8,9‐EET). For Figures 4C,E, 5E, 8B–D, 9C, and 12C–E and Figures S8–S10, S12, S14B, and S22, the mixture was additionally supplemented with 2000 pg *N*‐(2‐hydroxyethyl)‐5*Z*,8*Z*,11*Z*,14*Z*‐eicosatetraenamide‐5,6,8,9,11,12,14,15‐d8 (d8‐AEA).

### 5.6 Determination of Cell‐Free ALOX15 Activity

M2‐like macrophages (2×10^6^ cells) were resuspended in PBS pH 7.4 containing 1 mM EDTA and homogenized using a Q125 Sonicator (QSonica, Newtown, CT; 3 × 15 s; 35% power of 125 W). Complete homogenization was visually confirmed by light microscopy. Following a 15 min preincubation on ice with vehicle (DMSO, 0.1%) or test compounds, the reaction was initiated by adding 2 mM CaCl_2_ and 20 µM AA/20:4. After 15 min at 37°C, the reaction was terminated by addition of 1 mL ice‐cold methanol containing 200 pg 5*S*‐hydroxy‐6E,8Z,11Z,14Z‐eicosatetraenoic‐5,6,8,9,11,12,14,15‐d8 acid as internal standard.

### 5.7 LTB_4_ Formation in Activated HEK‐293 Cells Overexpressing ALOX5 and ALOX5AP

Human female embryonic kidney (HEK)‐293 cells (#CRL‐1573, ATCC) were grown in high‐glucose DMEM (4.5 g/L; Thermo Fisher Scientific) supplemented with 10% FCS, 100 U/mL penicillin, and 100 µg/mL streptomycin at 37°C and 5% CO_2_. Cells were subcultured before reaching confluence using trypsin/EDTA (GE Healthcare, Munich, Germany). HEK‐293 cells were authenticated by Multiplexion (Friedrichshafen, Germany; December, 2020) using Single Nucleotide Polymorphism (SNP) profiling (Multiplex Cell Line Authentication, https://www.multiplexion.de/en/cell‐line‐testing‐service/multiplex‐human‐cell‐line‐authentication), tested for mycoplasma (latest in 2025), and routinely monitored for characteristic morphology.

For transient overexpression of ALOX5 or ALOX5 plus ALOX5AP, HEK‐293 cells (4 × 10^5^/well) were seeded in 6‐well plates and cultured for 24 h. Cells were transfected with either an empty control vector (pRP[EXP]‐EGFP‐CAG>hypBase) or the expression vectors pPB[EXP]‐CMV>ALOX5[NM_000698.5] and/or pPB[EXP]‐CMV>hALOX5AP[NM_001204406.2] using TurboFectin 8.0 (OriGene, Rockville, MD) following the manufacturer's protocol. Briefly, 1 µg vector DNA was diluted in 250 µL Opti‐MEM (Thermo Fisher Scientific) and mixed with 3 µL TurboFectin 8.0. After 15 min incubation at RT, the mixture was added dropwise to cells in 1 mL culture medium followed by incubation for 48 h. Expression of ALOX5 and ALOX5AP was confirmed by Western blotting [98]. Cells were then washed and preincubated for 15 min in 1.5 mL PBS pH 7.4 containing 1 mM CaCl_2_ with vehicle (DMSO, 0.1%) or test compounds. LTB_4_ formation was induced by adding A23187 (2.5 µM) and AA/20:4, 2 µM). After 15 min at 37°C, reactions were stopped with 2.5 mL ice‐cold methanol containing 200 pg each of 5*S*,12*R*‐dihydroxy‐6*Z*,8*E*,10*E*,14*Z*‐eicosatetraenoic‐6,7,14,15‐d4 acid and 5*S*‐hydroxy‐6*E*,8*Z*,11*Z*,14*Z*‐eicosatetraenoic‐5,6,8,9,11,12,14,15‐d8 acid as internal standards.

### 5.8 Solid‐Phase Extraction and UPLC‐MS/MS‐Based Profiling of Lipid Mediators

To allow for protein precipitation, samples were stored at −20°C for at least 1 h and then centrifuged (750 × g, 10 min, 4°C). Supernatants (PBMCs: 3 mL; HEK‐293 cells: 3.5 mL; M1/M2 macrophages: 5.5 mL; exudate: 2.8 mL; plasma—peritonitis: 2.4 mL; lung tissue homogenates: 270–350 µL; plasma—fibrosis: 260–400 µL) were mixed with acidified water (pH 3.5; PBMCs, exudate, and plasma—peritonitis: 8 mL; M1/M2 macrophages: 9 mL; lung tissue: 0.7–0.9 mL; plasma –fibrosis: 0.7–1.1 mL) and applied to conditioned (6 mL methanol) and equilibrated (2 mL water) solid‐phase extraction cartridges (Sep‐Pak Vac 6cc 500 mg/6 mL C‐18; Waters, Milford, MA). Cartridges were washed with water (6 mL) followed by *n*‐hexane (6 mL, 4°C), and lipid mediators were eluted with methyl formate (6 mL). After evaporation of the organic solvent (TurboVap LV; Biotage, Uppsala, Sweden), eluates were redissolved in methanol/water (1:1), centrifuged (750 × g, 10 min, 4°C, followed by twice 21 100 × g, 4°C, 5 min), and analyzed by UPLC‐MS/MS [63, 177].

Chromatographic separation of lipid mediators was carried out on an Acquity UPLC BEH C18 column (130Å, 1.7 µm, 2.1 × 100 mm, Waters) at 55°C using an ExionLC AD UHPLC system (Sciex, Framingham, MA). Mobile phase A consisted of methanol containing 0.01% acetic acid, and mobile phase B of water/methanol (90/10) with 0.01% acetic acid. The flow rate was 0.35 mL/min. For oxylipin analysis, the gradient was ramped from 35.6% to 84.4% A over 12.5 min, followed by 5 min of isocratic elution at 97.8% A (Figures 2 and 3; Figure S1E–H and Figures S2–S6) [63]. Alternatively, oxylipins and endocannabinoids were separated using a gradient increasing linearly from 35.6% to 84.4% A within 12.5 min, then to 87.0% A over 2.5 min, followed by 3 min of isocratic elution at 97.8% A [177].

Oxylipins and free PUFAs were analyzed in negative and endocannabinoids in positive ion mode, using scheduled multiple reaction monitoring (MRM) with polarity switching on a QTRAP 6500^+^ mass spectrometer (Sciex) equipped with an IonDrive Turbo V ion source and a TurboIonSpray probe for electrospray ionization (Sciex) [36, 62, 63, 179, 180]. The curtain, sheath, and auxiliary gas pressures were set to 40 psi; the collision gas was set to “medium”. The heated capillary temperature was 500°C, and the ion spray voltage was −4000 V and 4000 V in negative and positive mode, respectively. The MRM transitions used for quantitation and their corresponding parameters—retention time (rt), declustering potential (DP), entrance potential (EP), collision energy (CE), collision cell exit potential (CXP), and MRM detection window—are listed in Table 1.

**TABLE 1:** Quantitative MRM transitions of lipid mediators and endocannabinoids (QTRAP 6500).

Q1 [m/z]	Q3 [m/z]	rt [min]	ID	MRM window [s]	DP [V]	EP [V]	CE [eV]	CXP [V]
351.3	195.1	5.2	20‐OH‐LTB_4_		−80	−10	−24	−15
349.2	235.1	6.0	15‐ketoPGE_2_		−115	−6	−19	−11
369.3	169.1	6.3	TXB_2_	130	−80	−10	−22	−15
355.3	193.2	6.6	d4‐PGE_2_ (IS)	120	−80	−10	−25	−16
351.2	271.0	6.7	PGE_2_		−120	−10	−20	−13
351.3	189.1	6.8	PGD_2_		−120	−10	−20	−13
380.3	141.2	6.9	d5‐RvD_2_ (IS)	120	−80	−10	−23	−14
353.3	193.1	7.0	PGF_2α_	60	−80	−10	−34	−11
375.2	175.1	7.0	RvD2		−80	−10	−30	−13
351.2	235.1	7.3	LXA4		−80	−10	−20	−13
375.2	215.1	7.4	RvD1		−80	−10	−26	−13
356.3	115.2	7.4	d5‐LXA_4_ (IS)	120	−80	−10	−19	−14
333.2	200.1	7.9	PGJ_2_		−30	−10	−20	−20
333.5	271.0	7.9	PGA_2_		−30	−10	−20	−5
333.3	115.1	8.4	RvE4		−80	−10	−22	−13
359.2	153.1	8.7	AT‐PD1	120	−80	−10	−21	−9
335.2	195.1	9.2	LTB_4_‐isomers	120	−80	−10	−22	−13
359.2	153.1	9.3	PDX	120	−80	−10	−21	−9
335.2	201.0	9.3	5,15‐diHETE	60	−50	−10	−30	−13
359.2	199.1	9.4	RvD5	60	−80	−10	−21	−13
359.2	153.1	9.5	PD1	120	−80	−10	−21	−9
335.2	195.1	9.7	LTB_4_	120	−80	−10	−22	−13
361.5	143.1	9.7	RvD5_n−3DPA_		−60	−10	−20	−10
339.3	197.2	9.7	d4‐LTB_4_ (IS)	120	−80	−10	−22	−13
359.2	221.0	10.2	MaR2		−80	−10	−20	−12
315.2	200.1	10.4	15‐deoxy‐PGJ_2_		−50	−10	−20	−20
337.2	207.1	10.5	14.15‐DiHET	60	−60	−5	−20	−10
337.2	167.1	10.7	11.12‐DiHET	60	−30	−5	−30	−15
317.2	259.1	10.9	18‐HEPE		−80	−10	−16	−23
337.2	127.1	11.0	8.9‐DiHET	60	−60	−5	−30	−15
335.2	115.1	11.1	5S,6R‐diHETE	80	−80	−10	−20	−13
317.2	219.1	11.1	15‐HEPE	60	−80	−10	−18	−12
317.2	167.1	11.2	11‐HEPE	60	−80	−10	−19	−12
317.2	179.1	11.3	12‐HEPE	60	−80	−10	−19	−12
337.2	145.0	11.5	5.6‐DiHET	60	−70	−5	−20	−10
317.2	200.1	11.5	17.18‐EpETE	60	−40	−5	−15	−10
317.2	115.1	11.6	5‐HEPE	60	−80	−10	−18	−12
295.2	195.0	11.6	13‐HODE		−60	−10	−25	−13
295.2	171.0	11.6	9‐HODE		−60	−10	−19	−13
317.2	200.1	11.7	14.15‐EpETE	60	−40	−10	−20	−13
343.2	245.1	11.8	17‐HDHA	60	−80	−10	−17	−14
319.2	219.1	11.8	15‐HETE	60	−80	−10	−19	−12
317.2	200.1	11.9	8.9‐EpETE	60	−10	−10	−15	−15
319.2	167.1	12.0	11‐HETE	60	−80	−10	−21	−12
379.2	287.3	12.0	1‐AG		30	10	20	15
343.2	205.1	12.1	14‐HDHA	60	−80	−10	−17	−14
319.2	179.1	12.1	12‐HETE	60	−80	−10	−21	−12
343.2	141.1	12.3	7‐HDHA	60	−80	−10	−18	−15
345.2	247.1	12.4	17‐HDPA		−80	−10	−17	−14
345.2	207.1	12.4	14‐HDPA		−80	−10	−17	−14
319.2	219.2	12.4	14.15‐EET	60	−60	−10	−20	−10
343.2	241.1	12.4	19.20‐EpDPA	70	−40	−10	−20	−13
327.3	116.1	12.4	d8‐5S‐HETE (IS)	120	−80	−10	−17	−10
319.2	115.1	12.5	5‐HETE	60	−80	−10	−21	−12
345.2	143.1	12.5	7‐HDPA		−80	−10	−18	−15
319.2	167.2	12.6	11.12‐EET	60	−40	−10	−20	−10
343.2	193.0	12.6	13.14‐EpDPA	70	−40	−10	−20	−13
343.2	233.2	12.6	16.17‐EpDPA	70	−20	−5	−15	−20
343.2	153.2	12.7	10.11‐EpDPA	70	−40	−10	−20	−13
343.2	101.1	12.7	4‐HDHA	60	−80	−10	−17	−15
330.2	155.2	12.7	d11‐8.9‐EET (IS)	60	−30	−5	−20	−10
319.2	167.0	12.8	8.9‐EET	60	−40	−5	−20	−10
343.2	189.0	12.8	7,8‐EpDPA	70	−20	−5	−15	−10
319.2	191.1	13.0	5.6‐EET	60	−50	−5	−20	−15
348.2	287.4	13.6	AEA		20	10	20	15
356.3	63.2	13.6	d8‐AEA (IS)		30	10	20	10
379.1	287.4	13.8	2‐AG		40	10	20	15
387.2	294.2	13.8	d8‐2‐AG (IS)		50	10	20	20
301.3	257.1	14.0	EPA		−100	−10	−16	−18
300.3	62.0	14.1	PEA		30	10	20	10
326.5	62.1	14.5	OEA		30	10	20	40
327.3	283.1	14.7	DHA		−100	−10	−16	−18
311.3	267.1	14.8	d8‐AA (IS)		−100	−10	−16	−18
303.3	259.1	14.9	AA/20:4		−100	−10	−16	−18
329.3	285.1	15.3	DPA		−100	−10	−16	−18

AA/20:4, arachidonic acid; AG, arachidonoyl glycerol; AEA, arachidonoylethanolamide; AT, aspirin‐triggered; DHA, docosahexaenoic acid; DiHET, dihydroxyeicosatrienoic acid; DPA, docosapentaenoic acid; EET, epoxyeicosatrienoic acid; EPA, eicosapentaenoic acid; EpETE, epoxyeicosatetraenoic acid; EpDPA, epoxydocosapentaenoic acid; HDHA, hydroxydocosahexaenoic acid; HDPA, hydroxydocosapentaenoic acid; HEPE, hydroxyeicosapentaenoic acid; (di)HETE, (di)hydroxyeicosatetraenoic acid; HODE, hydroxyoctadecadienoic acid; IS, internal standard; LT, leukotriene; LX, lipoxin; MaR, maresin; OEA, oleoylethanolamide; PD, protectin; PEA, palmitoylethanolamide; PG, prostaglandin; Rv, resolvin; TX, thromboxane.

For Figures 4E, and 9C, Figure S12, and three datasets of Figure S14B), PUFAs, oxylipins, and endocannabinoids were analyzed by scheduled MRM^HR^ on a ZenoTOF 7600 mass spectrometer (Sciex) equipped with an OptiFlow Turbo V electrospray source (Sciex). Data acquisition was performed using Sciex OS 4.0 software. The instrument was operated in negative ion mode for the detection of PUFAs and oxylipins and in positive ion mode for the detection of endocannabinoids. Ion source gas 1 and 2 pressures were set to 60 and 75 psi, respectively, and curtain gas pressure was 55 psi. The heated capillary temperature was 550°C in positive ion mode and 575°C in negative ion mode. Ion spray voltages were set to 5200 and −4400 V in positive and negative ion mode, respectively. TOF‐MS experiments were acquired over a mass range of *m/z* 60–650 with an accumulation time of 0.1 s, using a declustering potential (DP) of 100 (positive mode) or ‐100 V (negative mode), and a collision energy (CE) of 10 eV (positive mode) or −10 eV (negative mode). Scheduled TOF‐MS/MS experiments (± 30 s window) were performed over a mass range of *m/z* 60 to 400 with an accumulation time of 0.01 s, and the Zeno threshold was set to 80 000 counts per second. The MRM^HR^ precursor and fragment *m/z* values used for quantitation, together with their corresponding parameters, are listed in Table 2. The instrument was automatically calibrated after every five injections using ESI Positive (#5049910) and Negative (#5042913) Calibration Solution for the SCIEX X500 System (Sciex).

**TABLE 2:** Quantitative MRM^HR^ transitions of lipid mediators and endocannabinoids (ZenoTOF 7600).

*m/z* precursor	*m/z* fragment	rt [min]	ID	DP	CE
351.2177	195.1027	4.67	20‐OH‐LTB_4_	−100	−25
349.2021	113.0975	5.57	15‐keto‐PGE_2_	−80	−29
369.2283	169.0878	5.8	TXB_2_	−60	−23
351.2177	271.2075	6.09	PGE_2_	−35	−23
355.2428	275.2326	6.1	d4‐PGE_2_ (IS)	−65	−23
351.2177	233.1189	6.2	PGD_2_	−35	−17
380.2491	175.075	6.4	d5‐RvD_2_ (IS)	−65	−31
353.2334	317.212	6.43	PGE_1_	−35	−19
353.2334	183.1214	6.43	PGF_2a_	−95	−35
356.2491	115.0402	6.81	d5‐LXA_4_ (IS)	−75	−21
335.2228	173.1341	8.79	5,15‐diHETE	−60	−20
335.2228	195.1033	9.15	LTB_4_	−50	−21
339.2479	197.2479	9.17	d4‐LTB_4_ (IS)	−70	−21
337.2384	207.139	9.91	14,15‐DiHET	−40	−23
337.2384	167.1075	10.18	11,12‐DiHET	−70	−26
337.2384	127.0758	10.44	8,9‐DiHET	−90	−31
335.2228	115.0402	10.54	5,6‐diHETE	−55	−22
317.2122	219.1398	10.56	15‐HEPE	−65	−18
317.2122	167.1083	10.61	11‐HEPE	−45	−21
317.2122	179.1085	10.75	12‐HEPE	−75	−19
337.2384	145.0502	10.96	5,6‐diHET	−70	−25
295.2279	195.1392	11.04	13‐HODE	−35	−25
317.2122	115.0402	11.06	5‐HEPE	−40	−19
295.2279	171.1026	11.09	9‐HODE	−75	−25
319.2279	175.1501	11.27	15‐HETE	−85	−21
319.2279	167.108	11.43	11‐HETE	−40	−21
343.2279	205.1234	11.54	14‐HDHA	−90	−15
319.2279	179.1081	11.55	12‐HETE	−75	−19
319.2279	219.1388	11.83	14.15‐EET	−70	−15
327.2781	116.0459	11.96	d8‐5‐HETE (IS)	−65	−21
319.2279	115.0402	11.97	5‐HETE	−35	−21
319.2279	167.1081	12.13	11.12‐EET	−55	−19
343.2279	153.0928	12.18	10,11‐EpDPA	−35	−18
330.2969	155.0709	12.23	d11‐8.9‐EET (IS)	−35	−17
319.2279	167.0712	12.26	8.9‐EET	−75	−17
319.2279	191.1806	12.44	5.6‐EET	−80	−17
348.2897	287.2364	13.1	AEA	60	21
356.3399	63.0656	13.1	d8‐AEA	40	26
301.2173	257.2269	13.48	EPA	−95	−17
300.2897	62.0599	13.55	PEA	55	21
326.3053	62.0599	13.9	OEA	40	23
327.2781	283.2428	14.14	DHA	−100	−17
303.223	259.2424	14.19	AA	‐ 50	19
311.2832	267.2923	14.25	AA‐d8 (IS)	‐ 90	‐ 17
329.2486	285.2577	14.63	DPA	‐ 55	‐ 21

AA/20:4, arachidonic acid; AEA, arachidonoylethanolamide; DHA, docosahexaenoic acid; DiHET, dihydroxyeicosatrienoic acid; DPA, docosapentaenoic acid; EET, epoxyeicosatrienoic acid; EPA, eicosapentaenoic acid; EpDPA, epoxydocosapentaenoic acid; HDHA, hydroxydocosahexaenoic acid; HEPE, hydroxyeicosapentaenoic acid; (di)HETE, (di)hydroxyeicosatetraenoic acid; HODE, hydroxyoctadecadienoic acid; IS, internal standard; LT, leukotriene; LX, lipoxin; OEA, oleoylethanolamide; PEA, palmitoylethanolamide; PG, prostaglandin; Rv, resolvin; TX, thromboxane.

External standards (Cayman Chemical): 5*S*,12*R*‐dihydroxy‐6*Z*,8*E*,10*E*,14Z‐eicosatetraenoic acid; (±)5‐hydroxy‐6*E*,8*Z*,11*Z*,14*Z*‐eicosatetraenoic acid, (±)‐5‐hydroxy‐6*E*,8*Z*,11*Z*,14*Z*,17*Z*‐eicosapentaenoic acid; 5*S*,12*R*,20‐trihydroxy‐6*Z*,8*E*,10*E*,14*Z*‐eicosatetraenoic acid; 5*S*,6*R*‐dihydroxy‐7*E*,9*E*,11*Z*,14*Z*‐eicosatetraenoic acid; 9α,15*S*‐dihydroxy‐11‐oxo‐prosta‐5*Z*,13*E*‐dien‐1‐oic acid; 9‐oxo‐11α,15*S*‐dihydroxy‐prosta‐5*Z*,13*E*‐dien‐1‐oic acid; 9,15‐dioxo‐11α‐hydroxy‐prosta‐5*Z*,13*E*‐dien‐1‐oic acid; 9α,11α,15*S*‐trihydroxy‐prosta‐5*Z*,13*E*‐dien‐1‐oic acid; 9‐oxo‐15*S*‐hydroxy‐prosta‐5*Z*,10,13*E*‐trien‐1‐oic acid; 9α,11,15*S*‐trihydroxythromba‐5*Z*,13*E*‐dien‐1‐oic acid; 10*R*,17*S*‐dihydroxy‐4*Z*,7*Z*,11*E*,13*E*,15*Z*,19*Z*‐docosahexaenoic acid; 10*R*,17*R*‐dihydroxy‐4*Z*,7*Z*,11*E*,13*E*,15*Z*,19*Z*‐docosahexaenoic acid; 10(*S*),17(*S*)‐dihydroxy‐4*Z*,7*Z*,11*E*,13*Z*,15*E*,19*Z*‐docosahexaenoic acid; 7*S*,8*R*,17*S*‐trihydroxy‐4*Z*,9*E*,11*E*,13*Z*,15*E*,19*Z*‐docosahexaenoic acid; 7*S*,16*R*,17*S*‐trihydroxy‐4*Z*,8*E*,10*Z*,12*E*,14*E*,19*Z*‐docosahexaenoic acid; 7*S*,17*S*‐dihydroxy‐4*Z*,8*E*,10*Z*,13*Z*,15*E*,19*Z*‐docosahexaenoic acid; 7,17‐dihydroxy‐8*E*,10*Z*,13*Z*,15*E*,19*Z*‐docosapentaenoic acid; 5*S*,15*S*‐dihydroxy‐6*E*,8*Z*,11*Z*,13*E*,17*Z*‐eicosapentaenoic acid; 13*R*,14*S*‐dihydroxy‐4*Z*,7*Z*,9*E*,11*E*,16*Z*,19*Z*‐docosahexaenoic acid; 5*S*,6*R*,15*S*‐trihydroxy‐7*E*,9*E*,11*Z*,13*E*‐eicosatetraenoic acid; (±)17‐hydroxy‐4*Z*,7*Z*,10*Z*,13*Z*,15*E*,19*Z*‐docosahexaenoic acid; 14*S*‐hydroxy‐4*Z*,7*Z*,10*Z*,12*E*,16*Z*,19*Z*‐docosahexaenoic acid; (±)7‐hydroxy‐4*Z*,8*E*,10*Z*,13*Z*,16*Z*,19*Z*‐docosahexaenoic acid; (±)4‐hydroxy‐5*E*,7*Z*,10*Z*,13*Z*,16*Z*,19*Z*‐docosahexaenoic acid; (±)‐18‐hydroxy‐5*Z*,8*Z*,11*Z*,14*Z*,16*E*‐eicosapentaenoic acid; 15*S*‐hydroxy‐5*Z*,8*Z*,11*Z*,13*E*,17*Z*‐eicosapentaenoic acid; 12*S*‐hydroxy‐5*Z*,8*Z*,10*E*,14*Z*,17*Z*‐eicosapentaenoic acid; (±)‐11‐hydroxy‐5*Z*,8*Z*,12*E*,14*Z*,17*Z*‐eicosapentaenoic acid; 15*S*‐hydroxy‐5*Z*,8*Z*,11*Z*,13*E*‐eicosatetraenoic acid; 12*S*‐hydroxy‐5*Z*,8*Z*,10*E*,14*Z*‐eicosatetraenoic acid; 11*S*‐hydroxy‐5*Z*,8*Z*,12*E*,14*Z*‐eicosatetraenoic acid; 5*S*,15*S*‐dihydroxy‐6*E*,8*Z*,10*Z*,13*E*‐eicosatetraenoic acid; (±)‐9‐hydroxy‐10*E*,12*Z*‐octadecadienoic acid; (±)‐13‐hydroxy‐9*Z*,11*E*‐octadecadienoic acid; (±)5,6‐epoxy‐8*Z*,11*Z*,14*Z*‐eicosatrienoic acid; (±)8,9‐epoxy‐5*Z*,11*Z*,14*Z*‐eicosatrienoic acid; (±)11,(12)‐epoxy‐5*Z*,8*Z*,14*Z*‐eicosatrienoic acid; (±)14(15)‐epoxy‐5*Z*,8*Z*,11*Z*‐eicosatrienoic acid; (±)‐(4*Z*)‐6‐[3‐(2*Z*,5*Z*,8*Z*,11*Z*)‐2,5,8,11‐tetradecatetraen‐1‐yl‐2‐oxiranyl]‐4‐hexenoic acid; (±)‐(4*Z*,7*Z*)‐9‐[3‐(2*Z*,5*Z*,8*Z*)‐2,5,8‐undecatrien‐1‐yl‐2‐oxiranyl]‐4,7‐nonadienoic acid; (±)‐(4*Z*,7*Z*,10*Z*)‐12‐[3‐(2Z,5Z)‐2,5‐octadien‐1‐yl‐2‐oxiranyl]‐4,7,10‐dodecatrienoic acid; (±)16,17‐epoxy‐4*Z*,7*Z*,10*Z*,13*Z*,19*Z*‐docosapentaenoic acid; (±)19,20‐epoxy‐4*Z*,7*Z*,10*Z*,13*Z*,16*Z*‐docosapentaenoic acid; (±)5,6‐dihydroxy‐8*Z*,11*Z*,14*Z*‐eicosatrienoic acid; (±)8,9‐dihydroxy‐5*Z*,11*Z*,14*Z*‐eicosatrienoic acid; (±)11,12‐dihydroxy‐5*Z*,8*Z*,14*Z*‐eicosatrienoic acid; (±)14,15‐dihydroxy‐5*Z*,8*Z*,11*Z*‐eicosatrienoic acid; 5*Z*,8*Z*,11*Z*,14*Z*‐eicosatetraenoic acid; 5*Z*,8*Z*,11*Z*,14*Z*,17*Z*‐eicosapentaenoic acid, 4*Z*,7*Z*,10*Z*,13*Z*,16*Z*,19*Z*‐docosahexaenoic acid; 7*Z*,10*Z*,13*Z*,16*Z*,19*Z*‐docosapentaenoic acid; 5*Z*,8*Z*,11*Z*,14*Z*‐eicosatetraenoic acid,1‐glyceryl ester; 5*Z*,8*Z*,11*Z*,14*Z*‐eicosatetraenoic acid, 2‐glyceryl ester; *N*‐(2‐hydroxyethyl)‐5*Z*,8*Z*,11*Z*,14*Z*‐eicosatetraenamide; *N*‐(2‐hydroxyethyl)‐hexadecanamide; *N*‐(2‐hydroxyethyl)‐9*Z*‐octadecenamide; 9‐oxo‐15*S*‐hydroxy‐prosta‐8(12),13*E*‐dien‐1‐oic acid; (±)‐17(18)‐epoxy‐5*Z*,8*Z*,11*Z*,14*Z*‐eicosatetraenoic acid; (±)‐8(9)‐epoxy‐5*Z*,11*Z*,14*Z*,17*Z*‐eicosatetraenoic acid.

### 5.9 Determination of Human Recombinant ALOX5 Activity, Including Studies on Reversibility, Nuisance Inhibition, and Enzyme Kinetics

Human recombinant ALOX5 (10‐25 U; #60402, Cayman Chemical) was pretreated with vehicle (DMSO, 0.1%) or test compounds for 10 min on ice in 1 mL PBS pH 7.4 containing 1 mM EDTA and 1 mM ATP. The enzymatic reaction was initiated by adding AA/20:4 (20 µM or 0, 3, 10, 30, 100, and 300 µM for kinetic studies; Cayman Chemical) and CaCl_2_ (2 mM) [63, 64]. Triton X‐100 (0.01%, Thermo Fisher Scientific) was included in the reaction buffer to expose nuisance inhibitors. To discriminate between reversible and irreversible ALOX5 inhibitors, the reaction mixture (100 µL) was diluted 10‐fold in PBS pH 7.4 containing 1 mM EDTA, 1 mM ATP, and either vehicle (DMSO, 0.1%) or test compounds prior to addition of AA/20:4 (20 µM) and CaCl_2_ (2 mM) [91]. After 10 min at 37°C, the reaction was terminated by addition of 1 mL ice‐cold methanol, and prostaglandin B_1_ (PGB_1_; 200 ng; Cayman Chemical) was added as internal standard.

Lipid mediators were extracted using Clean‐Up C‐18 Endcapped SPE cartridges (100 mg, 10 mL, UCT, Bristol, PA). Cartridges were conditioned twice with 1 mL methanol and equilibrated with 1 mL water. Acidified samples (530 µL PBS plus 60 mM HCl) were centrifuged (750 × g, 10 min, 4°C), and the supernatants were applied to the cartridges. The cartridges were then washed with 1 mL water followed by 1 mL methanol/water (75/25), and lipid mediators were eluted with 300 µL methanol. Eluates were diluted with 120 µL water, centrifuged (21,100 × g, 10 min, 4°C), and subjected to LC‐PDA analysis [63, 64].

Chromatographic separation of ALOX5‐derived lipid mediators (all‐*trans* isomers of LTB_4_ and 5‐HETE, 12‐HETE, and 15‐HETE) was performed on a Kinetex C‐18 LC column (100 Å, 1.3 µm, 2.1 × 50 mm, Phenomenex, Torrance, CA) using a Nexera X2 UHPLC system (Shimadzu, Kyoto, Japan) operated at 40°C and a flow rate of 0.45 mL/min. Solvent A consisted of 50% methanol/50% water/0.05% trifluoroacetic acid, and solvent B was 100% methanol/0.05% trifluoroacetic acid. The initial mobile phase composition was 86% A and 14% B. After 2 min of isocratic elution, the proportion of mobile phase B increased stepwise to 46% over 2 min, and then to 90% over the subsequent 2 min. LTB_4_ isomers, 5‐HETE, 12‐HETE, 15‐HETE, and PGB_1_ were quantified using a photodiode array detector (SPD‐M20A, Shimadzu) at 280 nm (LTB_4_ isomers and PGB_1_) or 235 nm (HETEs). Chromatograms were acquired and processed using LabSolutions (version 5.97, Shimadzu), and lipid quantities were calculated by one‐point internal calibration using PGB_1_ [63, 64].

For the enzyme kinetic studies shown in Figure 5C and Figure S13, ALOX5 products were separated on a TFS Vanquish Core HPLC‐System (Thermo Fisher Scientific), using the same column, column temperature and solvents as described above for UPLC‐MS/MS‐based lipid mediator profiling, but with a flow rate of 0.33 mL/min and an extended re‐equilibration period at the end of the gradient (4.8 instead of 3 min). ALOX5‐derived LTB_4_ isomers and 5‐HETE were detected and quantified by scheduled selected ion monitoring (SIM) as the respective precursor ion (for all‐*trans* LTB_4_ isomers: *m/z* = 335.1; rt: 11.5 min and 11.7 min; dwell time: 0.02 s; SIM width: 0.1 amu; CID voltage: 20 V; for 5‐HETE: *m/z* = 319.2; rt: 15.1 min; dwell time: 0,02 s; SIM width 0.1 amu; CID voltage: 0 V), using an ISQ EM single quadrupole mass spectrometer (Thermo Fisher Scientific) operated in negative HESI mode with a source voltage of ‐3500 V. PGB_1_ (*m/z* = 335.1; rt: 10.7 min; dwell time: 0.02 s; SIM width: 0.1 amu; CID voltage: 10 V) was used as internal standard. Sheath gas, auxiliary gas, and sweep gas were set to 25, 6.2 and 1.5 psi, respectively. Vaporizer temperature was 375°C, and ion transfer tube temperature was 325°C. The instrument was operated and data were processed using Chromeleon 7.3 software (Thermo Fisher Scientific). Product concentrations were calculated from a 7‐ or 8‐point standard curve and normalized to PGB_1_.

### 5.10 Quantitative Analysis of Cytokine Expression

Freshly isolated human PBMCs (1.4 × 10^6^ cells/mL in 1 mL RPMI 1640 medium supplemented with 5% FCS, 2 mmol/L *L*‐glutamine, 100 U/mL penicillin and 100 µg/mL streptomycin) were pretreated for 30 min with vehicle (DMSO, 0.1%), test compounds, or dexamethasone (10 µM) and subsequently challenged with LPS (*Escherichia coli* O127:B8; Sigma–Aldrich) at 10 ng/mL for 4 h (TNF‐α, IL‐8) or 18 h (IL‐1β, IL‐1ra, IL‐6, MCP‐1, IL‐10, IL‐12 (p70), IL‐23) [181]. Supernatants were collected by centrifugation (21 000 × g, 5 min, 4°C), and cytokine/chemokine levels were quantified. TNF‐α, IL‐1β, IL‐6, IL‐8, MCP‐1, and IL‐10 were measured using in‐house ELISA systems based on DuoSet ELISA Development kits (Bio‐Techne, Minneapolis, MN) according to the manufacturer's instructions. IL‐1ra, IL‐12 (p70), and IL‐23 were analyzed using a Bio‐Plex 200 System with Bio‐Plex Pro Human Cytokine Singleplex Sets, Bio‐Plex Pro Reagent Kit III with Flat Bottom Plate, and Bio‐Plex Pro Human Cytokine Screening Panel Standards (BIO‐RAD, Hercules, CA). Cytokine and chemokine concentrations were determined using 8‐point standard curves.

Cytokine levels (TNF‐α, IL‐1β and IL‐10) in peritoneal exudates were quantified by ELISA using mouse DuoSet ELISA Development Kits (Bio‐Techne) as described above.

### 5.11 Extraction of Membrane and Neutral Lipids and Meroterpenoids

Phospholipids, neutral lipids, sphingolipids, FFA, and meroterpenoids **2** and **3** were extracted from cell pellets (PBMCs), peritoneal exudates (150 µL), or mouse plasma (50 µL) by sequential addition of PBS pH 7.4, methanol, chloroform, and saline in a final ratio of 14:34:35:17 [25, 182, 183]. The organic phase was evaporated using an Eppendorf Concentrator Plus System (Eppendorf, Hamburg, Germany; high vapor pressure application mode). The resulting lipid film was dissolved in methanol, diluted, and centrifuged (21,100 × g, 4°C, 5 min; twice), and the supernatant was subjected to UPLC‐MS/MS analysis.

Internal standards (Sigma–Aldrich): i. PBMCs (48 h treatment with meroterpenoids): 1,2‐dimyristoyl‐*sn*‐glycero‐3‐phosphatidylcholine; 1,2‐dimyristoyl‐*sn*‐glycero‐3‐phosphatidylethanolamine; 1,2‐dimyristoyl‐*sn*‐glycero‐3‐phosphatidylglycerol; 1,2‐myristoyl‐*sn*‐glycero‐3‐phosphoserine; 1,2‐dioctanoyl‐*sn*‐glycero‐3‐phospho‐(1'‐myo‐inositol), (15,15,16,16,17,17,18,18,18‐d9)oleic acid; cholest‐5‐en‐3ß‐yl heptadecanoate; 1,2,3‐trimyristoyl‐*sn*‐glycerol: 1,2‐dimyristoyl‐*sn*‐glycerol, 1',3'‐bis[1,2‐dimyristoyl‐*sn*‐glycero‐3‐phospho]‐glycerol; *N*‐heptadecanoyl‐*D*‐erythro‐sphingosine; and *N*‐heptadecanoyl‐*D*‐erythro‐sphingosylphosphorylcholine. ii. DGAT and/or SOAT inhibition (48 h): 1,3‐dipentadecanoyl‐2‐oleyol(d7)‐glycerol and 25,26,26,26,27,27,27‐d7‐cholest‐5‐en‐3β‐ol (9Z‐octadecenoate). iii. Mouse peritonitis: *D*‐erythro‐sphingosine‐d7, *N*‐heptadecanoyl‐*D*‐erythro‐sphingosine, *D*‐glucosyl‐β‐1,1'‐*N*‐heptadecanoyl‐*D*‐erythro‐sphingosine, *N*‐lauroyl‐ceramide‐1‐phosphate, *N*‐heptadecanoyl‐*D*‐erythro‐sphingosylphosphorylcholine, and *D*‐erythro‐sphingosine‐d7‐1‐phosphate.

### 5.12 Quantitative Analysis of Neutral and Membrane Lipids and Meroterpenoids by UPLC‐MS/MS

Lipids (except S1P and cholesterol) were separated on an Acquity UPLC BEH C8 column (130 Å, 1.7 µm, 2.1 × 100 mm, Waters, Milford, MA) using an ExionLC AD UHPLC system (Sciex) [25, 182]. The chromatographic separation of PC, PE, PI, PG, TG, DG, CE, So, sphinganine (Sa), (dh)Cer, hexosylceramide (HexCer), C1P, (dh)SM, and FFA was performed at 45°C with a flow rate of 0.75 mL/min. PS was separated at 65°C and 0.85 mL/min, while CL was separated at 55°C and 0.60 mL/min. Glycerophospholipids, FFA, and sphingolipids were eluted using mobile phase A (acetonitrile/water, 95/5, 2 mM ammonium acetate) and mobile phase B (water/acetonitrile, 90/10, 2 mM ammonium acetate). TG, DG, and CE were separated with mobile phase B consisting of 100% isopropanol. CL separation was achieved using methanol with 2 mM ammonium acetate as mobile phase A and water with 2 mM ammonium acetate as mobile phase B.

For the separation of PC, PE, PI, PG, PS, and FFA, the gradient was ramped from 75% to 85% A over 5 min and then to 100% A over an additional 2 min, followed by 2 min of isocratic elution. For sphingolipids, the isocratic step was extended to 13 min. TG, DG, and CE were separated using a mobile phase composition of A/B = 90/10, which was ramped to 70/30 over 6 min, followed by 4 min of isocratic elution [25]. For CL, the proportion of mobile phase A was increased from 85% to 98% over 8 min, followed by 1 min of isocratic elution.

Eluted lipids were detected using a QTRAP 6500^+^ mass spectrometer (Sciex) equipped with an IonDrive Turbo V Ion Source and a TurboIonSpray probe for electrospray ionization. PC ([M+OAc]^−^ to fatty acid anions) [184], other glycerophospholipids ([M‐H]^−^ to fatty acid anions) [184], and CL ([M‐2H]^2−^ to fatty acid anions) were analyzed by MRM in negative ion mode. FFA were quantified by single ion monitoring in the negative ion mode [25]. TG, DG, and CE were analyzed by MRM ([M+NH_4_]^+^ to [M‐fatty acid anion]^+^) in the positive ion mode [25, 185]. Sphingolipids were quantified by scheduled MRM using the following transitions: [M+H]^+^ to [M+H‐H_2_O]^+^ for So, Sa, and dhCer); m/z = 184.1 for [dh]SM; and m/z = 264.4 for Cer, HexCer, and C1P [182]. Source‐ and compound‐specific parameters for both positive and negative ion modes are listed in Table 3.

**TABLE 3:** QTRAP 6500^+^ parameters for targeted lipidomics.

Negative ion mode	PC	PE	PG	PI	PS	CL	FFA
Curtain gas [psi]	40	40	40	40	40	40	40
Collision gas	medium	medium	medium	medium	medium	medium	
Ion‐spray voltage [V]	−4500	4500	4500	4500	4500	4500	4500
Heated capillary temperature [°C]	350	650	550	500	550	650	500
Sheath gas [psi]	55	55	55	55	45	40	60
Auxiliary gas [psi]	75	75	75	75	80	80	80
Declustering potential [V]	−44	−50	−45	−50	−40	−44	−45
Entrance potential [V]	−10	−10	−10	−10	−10	−10	−10
Collision energy [eV]	−46	−38	−52	−62	−56	−40	
Collision cell exit potential [V]	−11	−12	−18	−11	−20	−12	

Positive ion mode	TG	DG	CE	TG+CE	So/Sa	(dh)Cer	HexCer	C1P	(dh)SM
Curtain gas [psi]	40	40	40	40	40	40	40	40	40
Collision gas	low	low	low	low	medium	medium	medium	medium	medium
Ion‐spray voltage [V]	5500	5500	5500	5500	5000	5000	5000	5000	5000
Heated capillary temperature [°C]	400	400	350	375	500	500	500	500	500
Sheath gas [psi]	60	60	55	60	40	40	40	40	40
Auxiliary gas [psi]	70	70	75	70	40	40	40	40	40
Declustering potential [V]	120	120	55	120/55	30	30	40	30	40
Entrance potential [V]	10	10	10	10	10	10	5	10	10
Collision energy [eV]	35	35	22	35/22	20	40	50	40	30
Collision cell exit potential [V]	26	26	22	26/22	25	20	20	5	10

Deviating from the procedures described above, S1P, cholesterol, and meroterpenoids **2** and **3** were separated on an Acquity UPLC CSH C18 column (130Å, 1.7 µm, 2.1 × 50 mm, Waters) at 55°C and a flow rate of 0.55 mL/min. S1P, **2**, and **3** were eluted isocratically using a mixture of water/acetonitrile/isopropanol (32/20/48) containing 0.1% formic acid. For cholesterol, the initial mobile phase composition of 40% A (water/acetonitrile (80/20) with 0.1% formic acid) and 60% mobile phase B (isopropanol/acetonitrile 80/20 with 0.1% formic acid) was held for 3 min, then B was increased linearly to 70% within 2 min and to 100% within 0.4 min, followed by 1.6 min isocratic elution.

Detection of S1P, cholesterol, and compounds **2** and **3** was performed in positive ion mode using a QTRAP 6500^+^ mass spectrometer (Sciex) equipped with an IonDrive Turbo V Ion Source and a TurboIonSpray probe for electrospray ionization. Quantitation was based on the following transitions: m/z 380.3 → 264.2 for S1P ([M+H]^+^ to [M+H‐H_3_PO_4_‐H_2_O]^+^); m/z 359.2 → 169.3 for compound **2**; m/z 345.2 → 153.0 for compound **3**, and m/z 387.4 → 281.0 for cholesterol ([M+H]^+^ to [M‐C_6_H_15_O‐H_2_]^+^), using the instrument parameters listed in Table 4.

**TABLE 4:** QTRAP 6500^+^ parameters for the analysis of S1P, cholesterol, **2** and **3**.

Positive‐ mode	S1P	Cholesterol	2	3
Curtain gas [psi]	40	40	40	40
Collision gas	low	low	low	low
Ion‐spray voltage [V]	5500	5500	4500	5500
Heated capillary temperature [°C]	550	500	550	550
Sheath gas [psi]	60	45	60	60
Auxiliary gas [psi]	30	45	30	30
Declustering potential [V]	40	20	30	40
Entrance potential [V]	10	5	10	10
Collision energy [eV]	20	10	25	30
Collision cell exit potential [V]	20	20	10	15

Absolute and relative amounts of individual PC, PE, PG, PI, PS, CE, FFA, DG, and sphingolipid species were calculated from the mean of the analyzed transitions. For fragments of fatty acids occurring at multiple positions within a lipid species, the signal was divided by the number of positions. TG and CL quantitation was based on the most abundant and specific transition, corrected for repeated fatty acids as described above. Glycerophospholipids, neutral lipids, FFA, and sphingolipids were quantified by one‐point calibration using subclass‐specific internal standards and normalized to cell number or sample volume. For sphingolipids from murine plasma and exudates (except S1P), signals were normalized to a subclass‐specific internal standard and concentrations calculated from hyperbolic 11‐point external calibration curves using subclass‐specific standards. Concentrations of **2** and **3** in mouse plasma and peritoneal exudates were determined from linear 10‐point standard curves, normalized to an internal standard (*D*‐erythro‐sphingosine‐d7‐1‐phosphate), corrected for the recovery of **3** during extraction, and adjusted for sample volume.

Relative lipid intensities (i.e., proportions) were calculated by summing the absolute amounts of PC, PE, PG, PI, PS, CL, TG, DG, CE, FFA, or sphingolipid species separately (i.e., 100% each class) and expressing the amount of each individual lipid species as a percentage of this respective total. The cellular proportion of lipids containing a specific fatty acid (e.g., AA/20:4) was calculated by summing the relative proportions of all lipid species that contain the respective fatty acid (corrected for fatty acids occurring multiple times within the same molecule) and expressing this sum as a percentage of the total lipid subclass (= 100%). Mass spectra were acquired using Analyst 1.7.1 or 1.7.2 and processed using Analyst 1.6.3 (Sciex).

### 5.13 Zymosan‐Induced Peritonitis in Mice

Male CD‐1 mice (33–39 g, eight weeks old, Charles River Laboratories, Calco, Italy) were provided with standard rodent chow and water and allowed to acclimate for four days in a controlled environment with a 12‐h light/12‐h dark cycle at a constant temperature of 21 ± 2°C. All experiments were performed during the light phase, and mice were randomly assigned to experimental groups.

Mice received vehicle (2% DMSO in saline, 0.5 mL), **2** (10 mg/kg), or **3** (10 mg/kg) intraperitoneally (i.p.) 30 min prior to injection of zymosan (Sigma–Aldrich, 2 mg/mL in saline, 0.5 mL, i.p.). Mice were sacrificed by CO_2_ inhalation after 4 or 18 h, and peritoneal exudates and plasma were collected [186]. Cell numbers in the exudates were determined by vital trypan blue staining [186]. Lipid mediators (100 µL plasma, 500 µL exudate), sphingolipids (50 µL plasma, 150 µL exudate), and compounds **2** and **3** (50 µL plasma, 150 µL exudate) were extracted and quantified as described above.

### 5.14 Bleomycin‐Induced Pulmonary Fibrosis in Mice

Male C57BL/6J mice (8 weeks old, 23–25 g; Charles River Laboratories, Calco, Italy) were housed under standard conditions with free access to food and water and allowed to acclimate for four days in a controlled environment (12‐h light/12‐h dark cycle, 21 ± 2 °C). All procedures were performed during the light phase.

Mice were randomly assigned to three groups: vehicle control, bleomycin, or bleomycin plus compound **3**. Bleomycin (2 mg/kg, 0.1 mL; Selleckchem) was administered subcutaneously into the dorsal area three times per week for 2 weeks [131]. Control animals received equivalent volumes of saline (0.1 mL). Compound **3** (10 mg/kg, 0.5 mL, i.p.) or vehicle (1% DMSO in 0.5 mL saline) was injected 1 h prior to each bleomycin administration. After 2 weeks, mice were euthanized within 48 h of the last injection using an overdose of enflurane (5%), and plasma and lung tissues were collected for lipid mediator analysis.

For lipid mediator analysis, plasma (60‐100 µL) was mixed with 200–300 µL ice‐cold methanol, followed by solid‐phase extraction and quantitation as described above. Lung tissue (27‐35 mg) was homogenized in methanol (270‐350 µL) using 2 mL tubes with a 2.8 mm ceramic bead in a Precellys 24 Touch homogenizer (Bertin Technologies, Montigny‐le‐Bretonneux, France) at 4500 rpm for 10 s at RT.

### 5.15 Determination of Endocannabinoid Degrading Enzyme Activity

The activity of human recombinant FAAH (#10005196) and MGLL (#705192) was measured using commercially available inhibitor screening assay kits (Cayman Chemical) according to the manufacturers’ protocols.

### 5.16 Silencing of *MGLL* in MCF‐7 Cells and Induction of Lipid Mediator Formation

Human MCF‐7 breast cancer cells (8 × 10^4^ cells per well in 12‐well plates) were seeded in high‐glucose DMEM (4.5 g/L; Thermo Fisher Scientific) supplemented with 1 mM sodium pyruvate (Thermo Fisher Scientific) and 10 µg/mL human recombinant insulin (#I9278; Sigma–Aldrich) and allowed to adhere for 24 h. Cells were then transfected with *MGLL*‐targeting siRNA (10 nM; Oligo Duplex siRNA, SR307777, OriGene) or a universal scrambled siRNA control (OriGene). The MGLL silencing mix consisted of three distinct 27‐mer duplexes directed against the following sequences: 5’‐GAGCUAAAGGAAUCAGUGUACACUACA‐3’; 5’‐ GCCAAUCCUGAAUCUGCAACAACUUUC‐3’; 5’‐CUCCGUCUUCCAUGAAAUAAACAUGUG‐3’.

After 48 h, cells were rinsed with PBS pH 7.4 containing 1 mM CaCl_2_ and pre‐incubated in the same buffer for 15 min at 37°C with either vehicle (DMSO, 0.1%) or test compounds (10 µM). Subsequently, cells were stimulated with SACM (1%) for 3 h. For lipid mediator quantification, reactions were terminated by adding 2.5 mL ice‐cold methanol supplemented with deuterium‐labelled internal standards (Cayman Chemical), as described above for UPLC–MS/MS‐based lipid mediator profiling. Parallel samples were processed for Western blot analysis to assess MGLL knockdown efficiency.

### 5.17 Immunofluorescence Microscopy

Lipoxygenases and perilipin‐2 were visualized in immune cells following a procedure adapted from Jordan et al., [86]. M2‐like macrophages (8 × 10^5^) were seeded onto glass coverslips (0.16–0.19 mm; Thermo Fisher Scientific) coated with poly‐*D*‐lysine hydrobromide (Sigma–Aldrich; 30 min, 37°C, 5% CO_2_) in RPMI 1640 medium supplemented with 10% FCS, 2 mmol/L *L*‐glutamine, 100 U/mL penicillin, and 100 µg/mL streptomycin. After 30 min, the medium was replaced with PBS pH 7.4 containing 1 mM CaCl_2_, and cells were treated with vehicle (DMSO, 0.1%), SACM (0.5%), **2** (10 µM), or **3** (10 µM) for 90 min at 37°C and 5% CO_2_. Cells were fixed with 4% paraformaldehyde in PBS pH 7.4 (Sigma Aldrich, 20 min, RT), permeabilized with acetone (3 min, 4°C), and incubated with 0.1% Triton X‐100 in PBS pH 7.4 containing 1 mM CaCl_2_ (10 min, RT). After blocking with 10% Normal Goat Serum in PBS pH 7.4 with 0.1% sodium azide (#50197Z; Thermo Fisher Scientific, 30 min, RT), coverslips were incubated overnight at 4°C with a mouse monoclonal anti‐ALOX15 antibody (1:100, #ab119774, Lot: GR3322776‐8; Abcam, Cambridge, UK), washed repeatedly, and stained with Alexa Fluor 555 goat anti‐mouse IgG (1:500, #A21422, Lot: 10143952; Invitrogen, Carlsbad, CA) for 30 min at RT.

PBMCs (3.65×10^6^) were seeded onto poly‐*D*‐lysine‐coated glass coverslips in RPMI 1640 medium supplemented with 5% FCS, 2 mmol/L *L*‐glutamine, 100 U/mL penicillin, and 100 µg/mL streptomycin, incubated for 30 min at 37°C and 5% CO_2_, and treated with vehicle (DMSO, 0.1%), **3**, or the indicated inhibitors for 48 h. The culture medium was then replaced with PBS pH 7.4 containing 1 mM CaCl_2_, and cells were challenged with calcium ionophore A23187 for 5 min at 37°C. Cells were fixed with 4% paraformaldehyde in PBS pH 7.4 (Sigma Aldrich; 20 min, RT) and permeabilized with 0.1% Triton X‐100 in PBS pH 7.4 containing 1 mM CaCl_2_ (Thermo Fisher Scientific; 15 min, RT). After blocking with 10% Normal Goat Serum in PBS pH 7.4 with 0.1% sodium azide (Thermo Fisher Scientific) for 30 min at RT, coverslips were incubated overnight at 4°C with mouse anti‐ALOX5 (1:1000; #610695; Lot: 9067817; BD Biosciences, Franklin Lakes, NJ) and rabbit anti‐perilipin‐2 (1:100; #ab108323, Lot: GR3259785‐40; Abcam) antibodies. ALOX5 and perilipin‐2 were visualized by staining with Alexa Fluor 555 goat anti‐mouse IgG (1:500, #A21422, Lot: 10143952; Invitrogen) and Alexa Fluor 488 goat anti‐rabbit IgG (1:500, #11034, Lot: 10729174; Invitrogen), respectively, for 30 min at RT.

Samples were mounted with ProLong Gold Antifade Mountant with DAPI (Thermo Fisher Scientific) and visualized using a Zeiss AxioObserver Z1 microscope (Carl Zeiss, Jena, Germany) operated with Zen 2.6 (Carl Zeiss). The system was equipped with a Plan‐Apochomat 40x/1.4 Oil DIC (UV) VIS‐IR M27 objective (Carl Zeiss) and an Axiocam 702 (Carl Zeiss). Exposure times were kept constant for all acquired images. Maximum intensity projections of Z stacks (8–10 slices, Z = 1 µm) (Figures 5H, and 12B and Figure S32) or single slices (Z = 1 µm) (Figure S33) were exported with ZEN blue 3.7 (Carl Zeiss). For merged images, the DAPI channel was pseudo‐colored light blue, Alexa 555 was pseudo‐colored yellow (ALOX15) or violet (ALOX5), and Alexa 488 was pseudo‐colored green (perilipin‐2). Quantitative assessment of ALOX15 translocation and lipid body number was performed in Fiji [187] by counting cellular particles stained for ALOX15 (Threshold: 0–4000; Particle size: 0‐infinity; Circularity: 0–1) and perilipin‐2 (Threshold: n1 600–65535, n2 500–600, n3 325–65535; Particle size: 0‐infinity; Circularity: 0–1), respectively.

### 5.18 Determination of Protein Expression and Phosphorylation by SDS‐PAGE and Western Blotting

Freshly isolated PBMCs (3.84 × 10^6^) were serum‐starved overnight in RPMI 1640 medium supplemented with 2 mmol/L *L*‐glutamine, 100 U/mL penicillin, and 100 µg/mL streptomycin. Cells were then exposed to vehicle (DMSO, 0.1%) or test compounds in the presence of 2% FCS at 37°C and 5% CO_2_. After 30 min of pre‐incubation, cells were stimulated with 10 ng/mL LPS (*Escherichia coli* O127:B8; Sigma–Aldrich) for either 15 min or 1 h to assess IκBα, p‐IκBα, and NF‐κB levels. Following stimulation, cells were placed on ice, washed twice with ice‐cold PBS pH 7.4 (1 mL), and lysed in 100 µL lysis buffer (20 mM Tris‐HCl pH 7.4, 150 mM NaCl, 2 mM EDTA, 1% Triton X‐100, 5 mM sodium fluoride, 10 µg/mL leupeptin, 60 µg/mL soybean trypsin inhibitor, 2.7 mM sodium vanadate, 2.5 mM sodium pyrophosphate, and 1 mM phenylmethanesulfonyl fluoride). Lysates were sonicated on ice (2 × 5 s at 35% of 125 W; Q125 Sonicator, QSonica, Newtown, CT).

For the analysis of ALOX5, ALOX5AP, and MEK1/2 expression as well as MEK1/2 phosphorylation, PBMCs (1×10^7^) were resuspended in RPMI 1640 medium supplemented with 5% FCS, 2 mmol/L *L*‐glutamine, 100 U/mL penicillin, and 100 µg/mL streptomycin. Cells were preincubated with vehicle (DMSO, 0.1%) or test compounds at 37°C for 48 h. After incubation, cells were harvested by sequential rinses with ice‐cold PBS pH 7.4 containing 5 mM EDTA (1 mL) followed by ice‐cold PBS pH 7.4 (1 mL), washed twice with ice‐cold PBS pH 7.4, and centrifuged (2000 × g, 7 min, 4°C). Cell pellets were resuspended in 100 µL lysis buffer (20 mM Tris‐HCl pH 7.4, 150 mM NaCl, 2 mM EDTA, 1% Triton X‐100, 5 mM sodium fluoride, 10 µg/mL leupeptin, 60 µg/mL soybean trypsin inhibitor, 1 mM sodium vanadate, 2.5 mM sodium pyrophosphate, and 1 mM phenylmethanesulfonyl fluoride) and sonicated on ice (2 × 5 s at 35% of 125 W; Q125 Sonicator, QSonica).

To assess MGLL knockdown efficiency in control‐ and siRNA‐transfected MCF‐7 cells, the culture medium was removed and cells were lysed in 100 µL lysis buffer (20 mM Tris‐HCl pH 7.4, 150 mM NaCl, 2 mM EDTA, 1% Triton X‐100, 5 mM sodium fluoride, 10 µg/mL leupeptin, 60 µg/mL soybean trypsin inhibitor, 2.7 mM sodium vanadate, 2.5 mM sodium pyrophosphate, and 1 mM phenylmethanesulfonyl fluoride). Cell lysates were collected by scraping.

Cell lysates were centrifuged (1200 × g, 5 min, 4°C), total protein levels were determined using a DC protein assay kit (Bio‐Rad Laboratories GmbH, Munich, Germany), and samples were adjusted to equal protein concentrations. Lysates were mixed with 5× SDS/PAGE sample loading buffer (125 mM Tris‐HCl pH 6.5, 25% sucrose, 5% SDS, 0.25% bromophenol blue, 5% β‐mercaptoethanol) and heated at 95°C for 5 min. Aliquots (6‐30 µg protein, depending on the target) were resolved on 10% or 12% SDS‐PAGE gels, and proteins were transferred to Cytiva Amersham Protran 0.45 µm NC nitrocellulose blotting membranes (GE Healthcare, Munich, Germany) [25, 188].

Membranes were blocked for 1 h at RT in either 5% bovine serum albumin (Roth, Karlsruhe, Germany) or 5% skim milk (Sigma Aldrich) prepared in TBS pH 7.4 containing 1% Tween‐20, and subsequently incubated overnight at 4°C with the following primary antibodies: mouse anti‐IκBα (1:800; #4814S; Cell Signaling, Danvers, MA), rabbit anti‐phospho‐IκBα (1:1000; #2859; Cell Signaling), rabbit anti‐NF‐κB (1:500; #4764; Cell Signaling), mouse anti‐ALOX5 (1:1000; #610695; Lot: 9067817; BD Biosciences), rabbit anti‐MEK1/2 (1:1000; #9122; Cell Signaling), rabbit anti‐phospho‐MEK1/2 (1:1000; #9121; Cell Signaling), goat anti‐ALOX5AP (1:1000; #ab53536; Lot: GR67416‐18; Abcam), rabbit anti‐MGLL (1:1000; #ab307162; Lot: 1032648‐3; Abcam), rabbit anti‐β‐actin (1:1000; #4970; Lot: 19; Cell Signaling), and mouse anti‐β‐actin (1:1000; #3700; Lot: 21; Cell Signaling).

After three washes with TBS pH 7.4 plus 1% Tween‐20, membranes were incubated for 1 h at RT with fluorophore‐conjugated secondary antibodies: DyLight 800 anti‐mouse IgG (1:10000; #SA5‐10176; Lot: VE2995014; Thermo Fisher Scientific), DyLight 800 anti‐rabbit IgG (1:10000; #SA5‐10036; Lot: VC2960806; Thermo Fisher Scientific), DyLight 680 anti‐mouse IgG (1:10000; #35519; Lot: VB298075; Thermo Fisher Scientific), DyLight 680 anti‐rabbit IgG (1:10000; #35569; Lot: VB302113; Thermo Fisher Scientific), and Alexa Fluor 680 anti‐goat IgG (1:8333; #ab175776; Lot: GR3459994‐1; Abcam).

Fluorescence signals were detected using a Fusion FX7 Edge Imaging System (VILBER Lourmat, Collegien, France; spectra light capsules: C680, C780; emission filters: F‐750, F‐850) or a ChemiDoc MP Imaging system (BioRad, Hercules, CA; epi‐far red excitation at 650–675 nm, epi‐near IR excitation at 755–777 nm; emission filters 715/30 and 835/50). Densitometric analysis was performed using the Bio‐1D imaging software (version: 15.08c; VILBER Lourmat) with rolling ball background subtraction, or, alternatively, using ImageLab software (BioRad) with local background correction. Uncropped blots are shown in Figures S17, S23, and S34B. Protein levels were normalized to β‐actin or total kinase signals.

### 5.19 Statistics and Network Analysis

Data are presented as mean, mean ± SEM, or mean ± SEM and individual data points from *n* independent experiments or animals (zymosan‐induced peritonitis and bleomycin‐induced fibrosis). For datasets with comparable variance between groups, normality was assessed using Shapiro‐Wilk tests, and outliers were identified using Grubb's tests, except when n = 3. A *p* value < 0.05 was considered statistically significant. Depending on data distribution, non‐transformed or logarithmically transformed data were used for statistical analyses. Two‐tailed (two‐sided α = 0.05) paired or unpaired Student *t* tests were applied for pairwise comparisons. Comparisons of independent or correlated multiple groups were performed using one‐way ANOVA, two‐way ANOVA, or mixed‐effects models (REML), followed by Dunnett's or Tukey's *post hoc* tests. For volcano plot analyses, negative log_10_(*q* values) were calculated from two‐tailed multiple (un)paired Student *t* tests with correction for multiple comparisons using a two‐stage step‐up procedure by Benjamini, Krieger, and Yekutieli. Data handling was performed using Microsoft Excel Version 2302 (Microsoft 365 Apps for Enterprise, Microsoft, Redmond, WA). Statistical analysis was performed using GraphPad Prism 9.5 or newer (GraphPad Software, San Diego, CA). Principal component analysis was performed using Origin 2022b SR1 (OriginLab, Northampton, MA).

The correlation‐based network was constructed using the MetScape 3.1.3 plugin for Cytoscape 3.9.1 (Cytoscape Consortium) [189, 190, 191]. Pearson correlation coefficients were calculated based on the mean percentage changes in absolute lipid amounts at 1 and 10 µM. Each node represents an individual lipid species, and edges represent correlation coefficients ≥ 0.7 (layout: Edge‐weighted Spring Embedded Layout based on the correlation matrix). Correlations between individual lipid species and lipid mediator subgroups (ALOX5 products, EETs) or AA/20:4 were calculated using Pearson correlation analysis in Microsoft Excel Version 2302 (Microsoft 365 apps for enterprise, Microsoft). Positive correlations (≥ 0.7) are depicted in red, and negative correlations (≤ ‐0.7) are depicted in blue.

### 5.20 Artificial Intelligence

During the preparation of this work, the authors used ChatGPT‐4.0, ChatGTP‐5.0, uniGTP‐5.1, and DeepL Write to improve readability and language. All tool‐generated content was subsequently reviewed and edited by the authors, who take full responsibility for the final version of the manuscript.

## Author Contributions

**Lorenz Waltl**—Methodology, Investigation, Visualization, Formal Analysis, Data Curation, Writing – original draft, Writing – review and Editing. **David Holubek**—Investigation, Visualization, Formal Analysis, Writing – Review and Editing. **Klaus Speck**—Methodology, Investigation, Formal Analysis. **Raphael Wildermuth**—Methodology, Investigation. **Franz‐Lucas Haut**—Methodology, Investigation. **Stephan Permann**—Methodology, Investigation, Analysis. **Immanuel Plangger**—Investigation, Formal Analysis. **Christian Steinborn**—Investigation, Formal Analysis. **Zhigang Rao**—Methodology, Writing – Review and Editing. **Danilo D'Avino**—Methodology, Investigation. **Ida Cerqua**—Methodology, Investigation. **Julia Stadler**—Methodology. **Fiorentina Roviezzo**—Methodology. **Peter Schlenke**—Resources. **Anita Siller**—Resources. **Harald Schennach**—Resources. **Solveigh C. Koeberle**—Methodology, Visualization, Formal Analysis, Writing – Review and Editing, Funding acquisition. **Eva‐Maria Pferschy‐Wenzig**—Methodology, Data Curation, Writing – Review and Editing. **Antonietta Rossi**—Methodology, Investigation, Supervision, Funding acquisition. **Thomas Magauer**—Resources, Writing – Review and Editing, Supervision, Funding acquisition. **Andreas Koeberle**: Project administration, Conceptualization, Data Curation, Writing – Original Draft, Writing – Review and Editing, Supervision, Funding acquisition.

## Funding Statement

Research activities of A.K. related to the subject of this article were funded in part by the Austrian Science Fund (FWF) (10.55776/I4968, 10.55776/P36299, 10.55776/PAT9018824). T.M. acknowledges funding from the European Research Council under the European Union's Horizon 2020 research and innovation program (grant agreements No 714049 and No 101000060) and from the Center for Molecular Biosciences (CMBI). A.R. acknowledges support from the Ministero dell'Università e della Ricerca (MUR), PRIN2022 PNRR (P2022CNPH8). S.C.K. was supported by the Austrian Science Fund (FWF) (DOI: 10.55776/RIC4054324). For the purpose of open access, the authors have applied a CC BY public copyright license to any Author Accepted Manuscript version arising from this submission.

## Ethics Statement

Studies on PBMCs, macrophages, and PMNLs were approved by the Ethical Committees of the Medical University Innsbruck (approval no. 1041/2020, June 19, 2020) and the University of Graz (approval no. 39/149/63 ex 2024/25, May 30, 2025) and were conducted with informed consent of the platelet and blood donors. All experimental procedures related to zymosan‐induced peritonitis and bleomycin‐induced fibrosis in mice were conducted in accordance to the guidelines outlined in the Italian (DL 26/2014) and European (Directive 2010/63/EU) regulations on the ethical use and protection of animals for scientific purposes, complied with the ARRIVE guidelines for the reporting of in vivo experiments, and were approved by the Italian Ministry.

## Conflicts of Interest

The authors declare no conflicts of interest.

## Supporting information




**Supporting File 1**: advs76861‐sup‐0001‐SuppMat.pdf.

**Supporting File 2**: advs76861‐sup‐0002‐TableS1‐S13.zip.

## Data Availability

The mass spectrometric lipidomics data generated in this study have been deposited in the Metabolomics Workbench database (an international repository for metabolomics data and metadata, metabolite standards, protocols, tutorials and training, and analysis tools [192]) under the accession code PR002799 [ST004429, ST004485, ST004486, ST004540, ST004487, ST004488, ST004489, ST004657; https://doi.org/10.21228/M8VN9M] and to MetaboLights [193] repository with the study identifiers MTBLS14992 and MTBLS15025. All other data that support the findings of the present study is either shown in the manuscript and Supporting Information or available from the corresponding author upon reasonable request.
