# Supplementary material for: Reorganization of Innate Immune Cell Lipid Profiles by Bioinspired Meroterpenoids to Limit Inflammation
Source: Adv Sci (Weinh). 2026 Aug 18;13(59):e76861. doi: 10.1002/advs.76861 (PMC13483160; doi:10.1002/advs.76861)
Supplement: Supplementary file 1 [file ADVS-13-e76861-s001.pdf]

Supporting information

Reorganization of innate immune cell lipid profiles by bioinspired meroterpenoids to limit inflammation

Waltl et al.

Supplementary figures

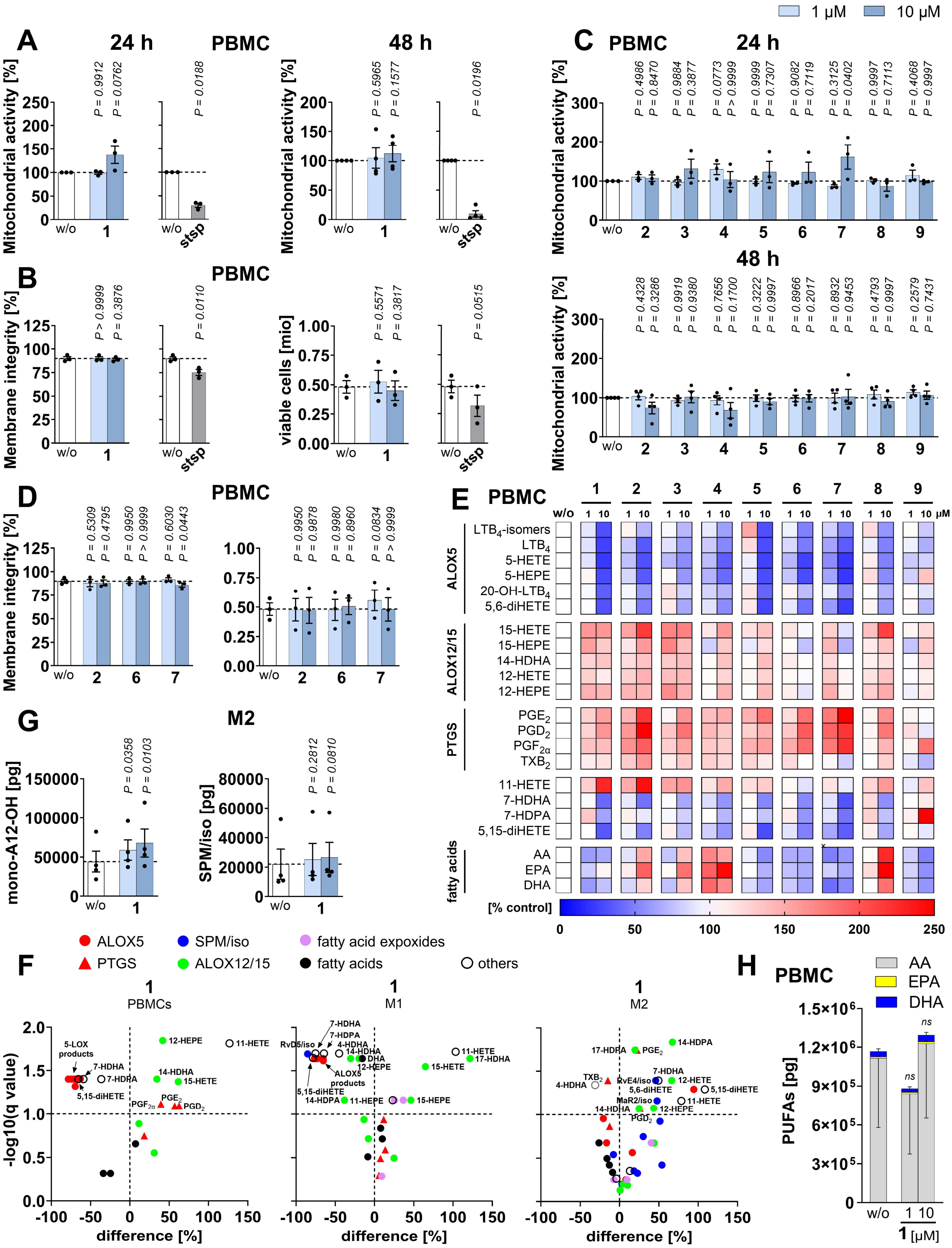

Figure S1. Meroterpenoids alter the lipid mediator profile of activated innate immune cells without inducing cytotoxicity. A,C) Changes in cellular dehydrogenase activity of PBMCs treated with vehicle (DMSO, 0.1%) or meroterpenoids for 24 h or 48 h (MTT assay). The vehicle control shown in C is identical to that shown in A. B,D) Membrane integrity of PBMCs treated with vehicle (DMSO, 0.1%) or meroterpenoids. E,F,H) PBMCs were preincubated (10 min) with vehicle (DMSO, 0.1%) or meroterpenoids and subsequently challenged with A23187 (10 min). E) The heatmap depicts percentage changes in lipid mediator concentrations relative to vehicle control. F,G) M1- or M2-like macrophages were preincubated (15 min) with vehicle (DMSO, 0.1%) or **1** (10 µM, unless indicated otherwise) and then activated with SACM (180 min). F) Volcano plots depict the mean percentage

difference relative to vehicle control and the negative $\log_{10}$(q value) calculated vs. vehicle control; two-tailed multiple paired Student t tests with correction for multiple comparisons (false discovery rate 10%). G) Mono-A12-OH and SPM/iso levels [per 2×10^6 M2-like macrophages]. H) Concentrations of the free PUFAs AA/20:4, eicosapentaenoic acid (EPA/20:5), and docosahexaenoic acid (DHA/22:6) [per 5×10^6 PBMCs]. Mean (E,F) or mean + SEM (H) and single data (A-D,G) from n = 3 (A 24 h,B,C 24 h,D), n = 3-4 (E,F,H), n = 4 (A 48 h,C 48 h,G) independent experiments. P values given vs. vehicle control (A-D, F-H); repeated measures one-way ANOVA of log data (A-D,G) + Dunnett *post hoc* tests or mixed-effects model (REML) of log data (H) + Dunnett *post hoc* tests. Staurosporine (‘stsp’, 1 µM) was used as cytotoxic control, two-tailed paired Student t test.

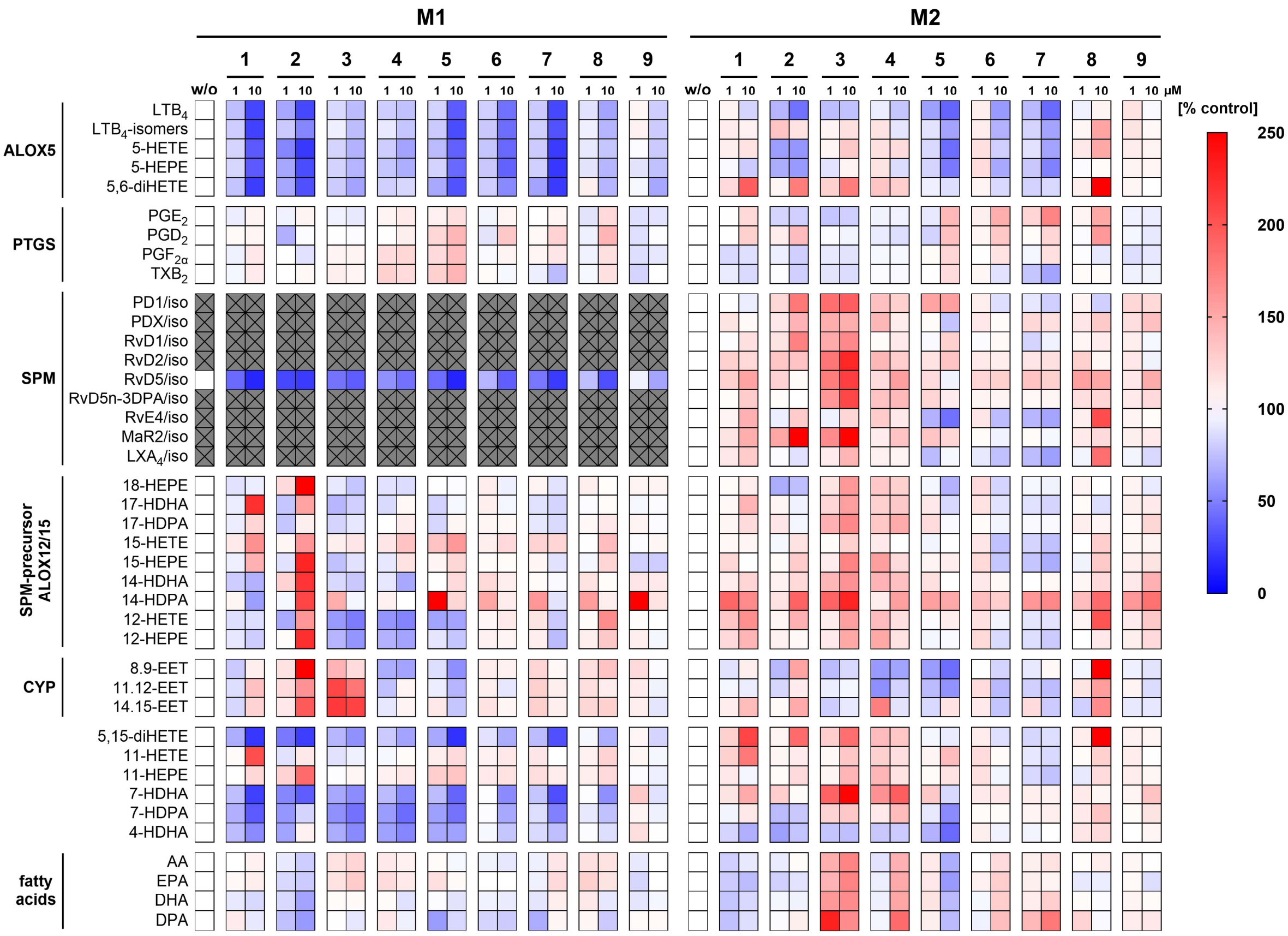

Figure S2. Effect of meroterpenoids on the lipid mediator profile of activated macrophages. M1- or M2-like macrophages were preincubated (15 min) with vehicle (DMSO, 0.1%) or meroterpenoids and subsequently activated with SACM (180 min). The heatmap depicts percentage changes in lipid mediator concentrations relative to vehicle control. Mean from n = 3-4 independent experiments.

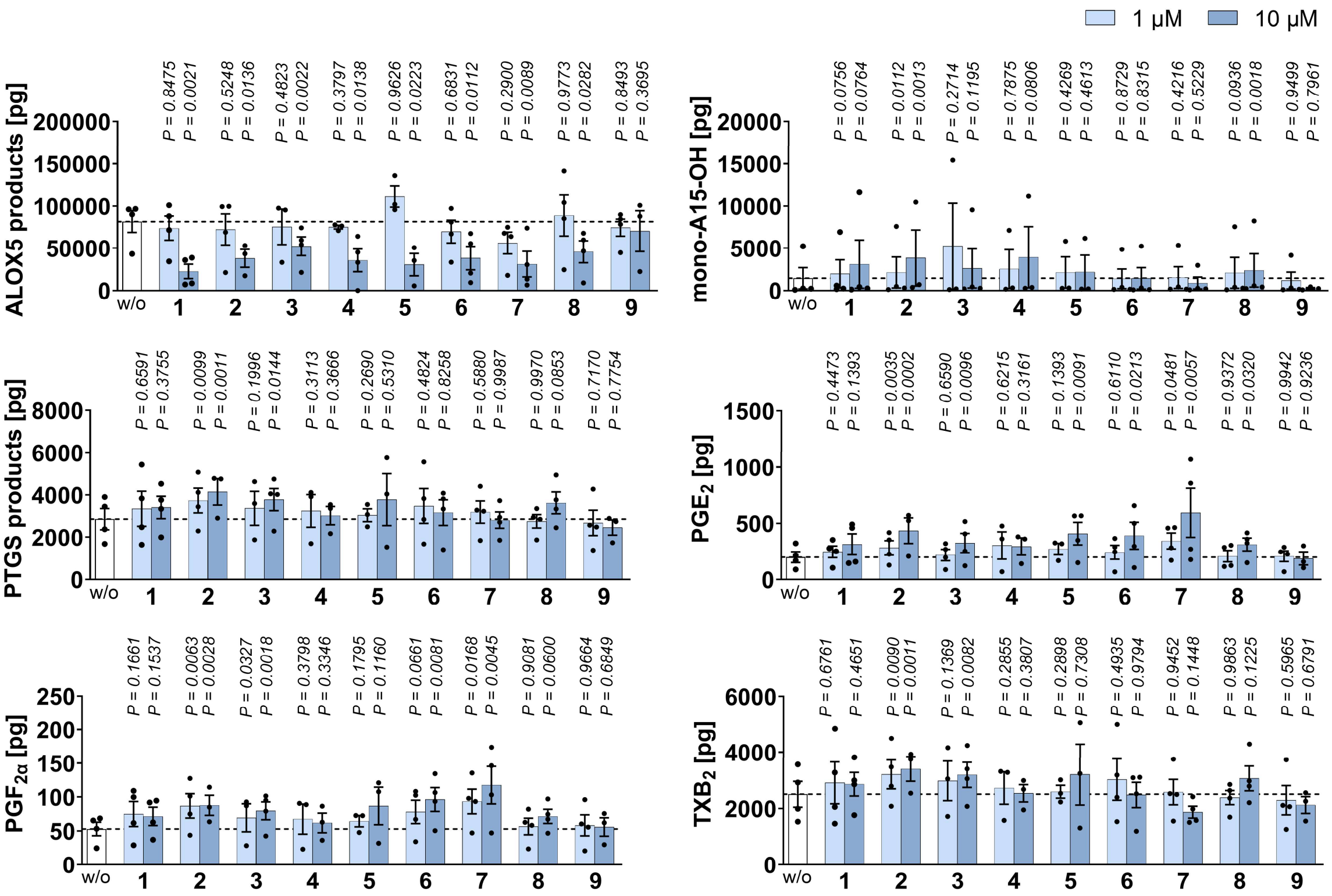

Figure S3. Effect of meroterpenoids on the biosynthesis of lipid mediator classes and selected species in activated innate immune cells. PBMCs were preincubated (10 min) with vehicle (DMSO, 0.1%) or meroterpenoids and subsequently challenged with A23187 (10 min). Shown are ALOX5 products, mono-A15-OH, PTGS products, and selected prostanoids [per 5×10^6 PBMCs]. Data for the vehicle control and **1** (ALOX5 and mono-A15-OH) are identical to those shown in Figure 2A. Mean + SEM and single data from n = 3-4 independent experiments. *P* values given vs. vehicle control; repeated measures one-way ANOVA of log data or mixed-effects model (REML) of log data + Dunnett post hoc tests.

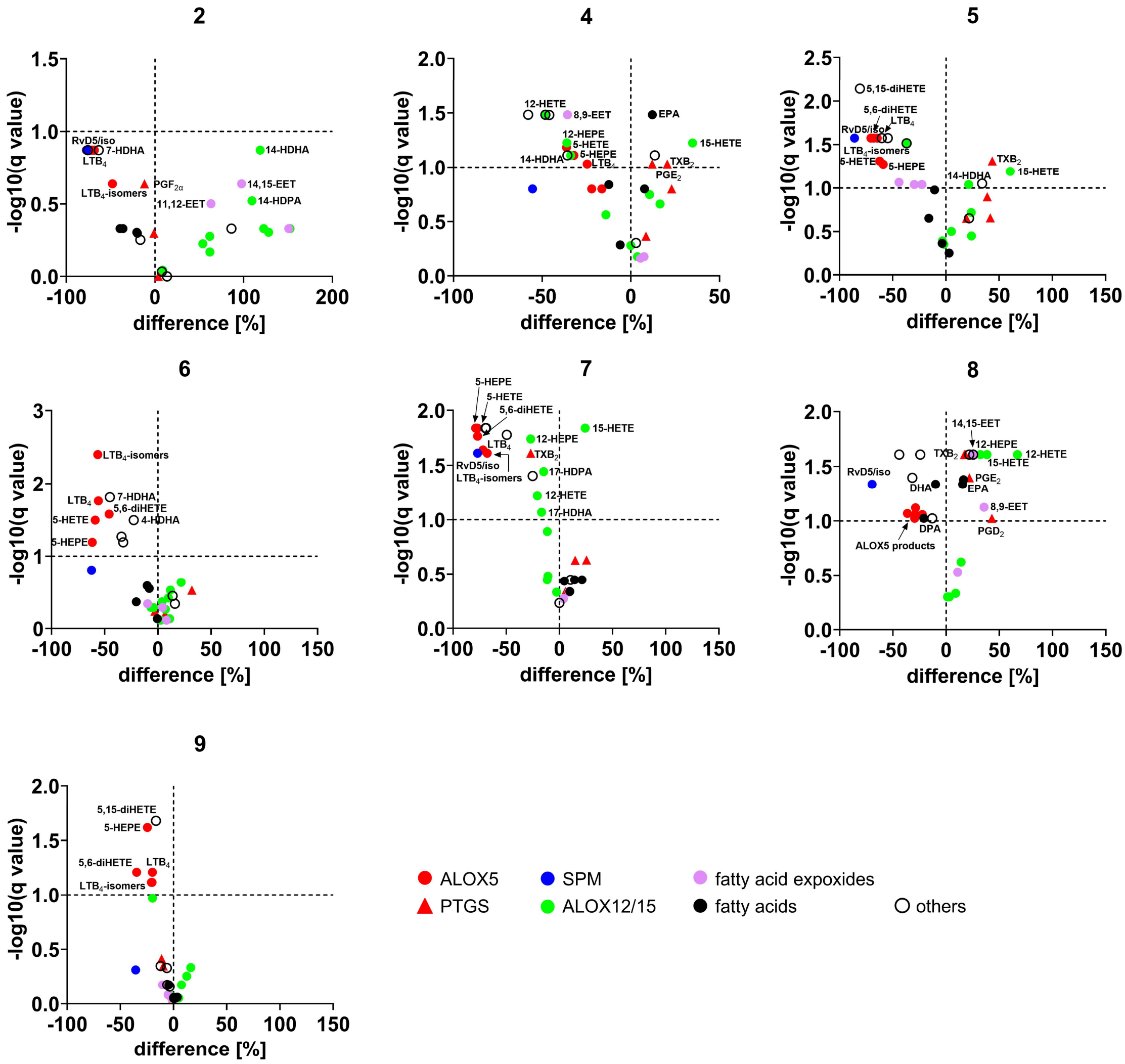

Figure S4. Extent and statistical significance of the effects of compounds **2** and **4-9** on lipid mediator levels in activated M1-like macrophages. Cells were preincubated (15 min) with vehicle (DMSO, 0.1%) or meroterpenoids (10 µM) and subsequently activated with SACM (180 min). Volcano plots depict the mean percentage difference relative to vehicle control and the negative $\log_{10}$(q value), calculated vs. vehicle control from n= 3-4 independent experiments; two-tailed multiple paired Student t tests with correction for multiple comparisons (false discovery rate 10%).

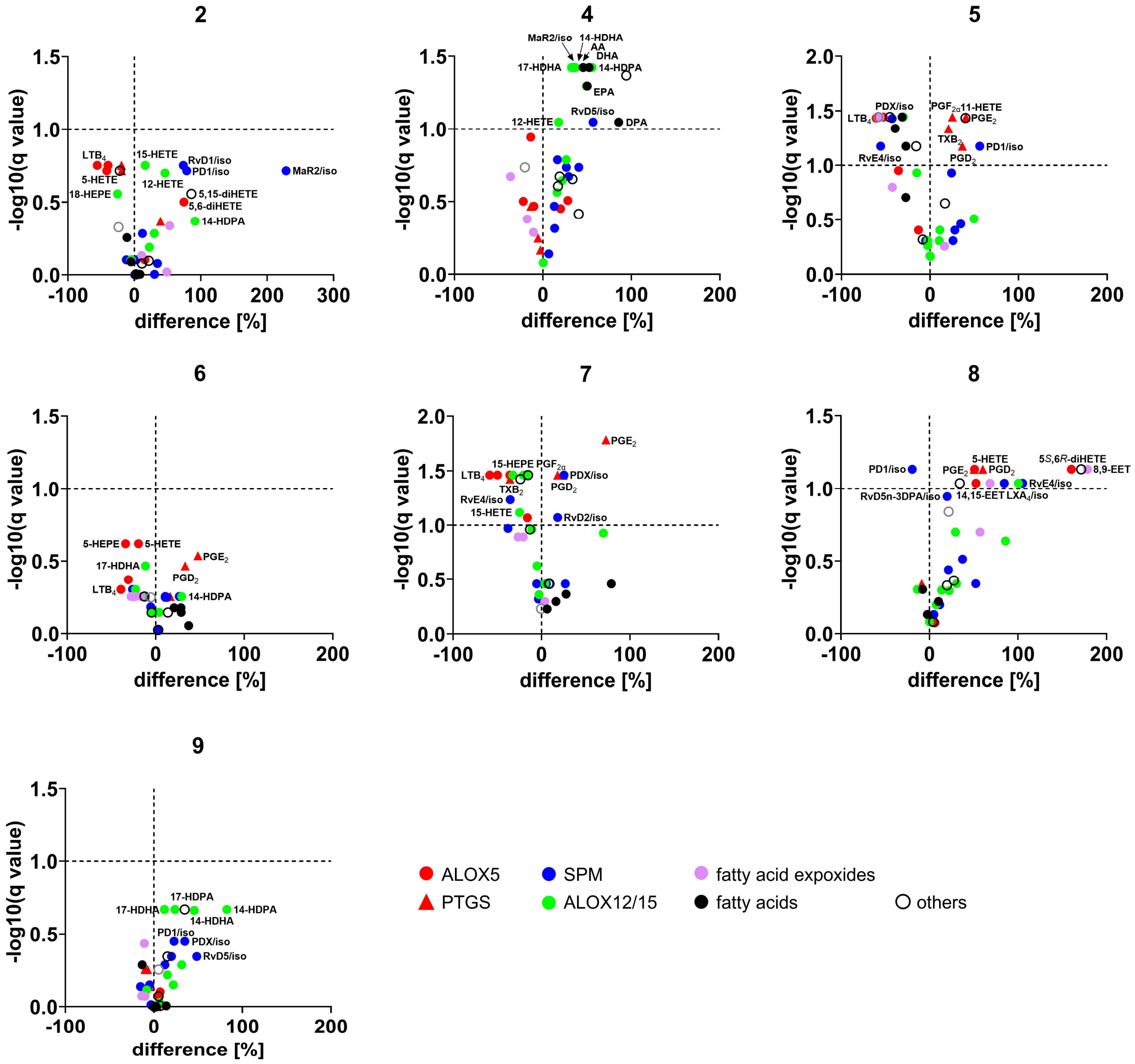

Figure S5. Extent and statistical significance of the effects of compounds **2** and **4-9** on lipid mediator levels in activated M2-like macrophages. Cells were preincubated (15 min) with vehicle (DMSO, 0.1%) or meroterpenoids (10 µM) and subsequently activated with SACM (180 min). Volcano plots depict the mean percentage difference relative to vehicle control and the negative $\log_{10}$(q value), calculated vs. vehicle control from n= 3-4 independent experiments; two-tailed multiple paired Student t tests with correction for multiple comparisons (false discovery rate 10%).

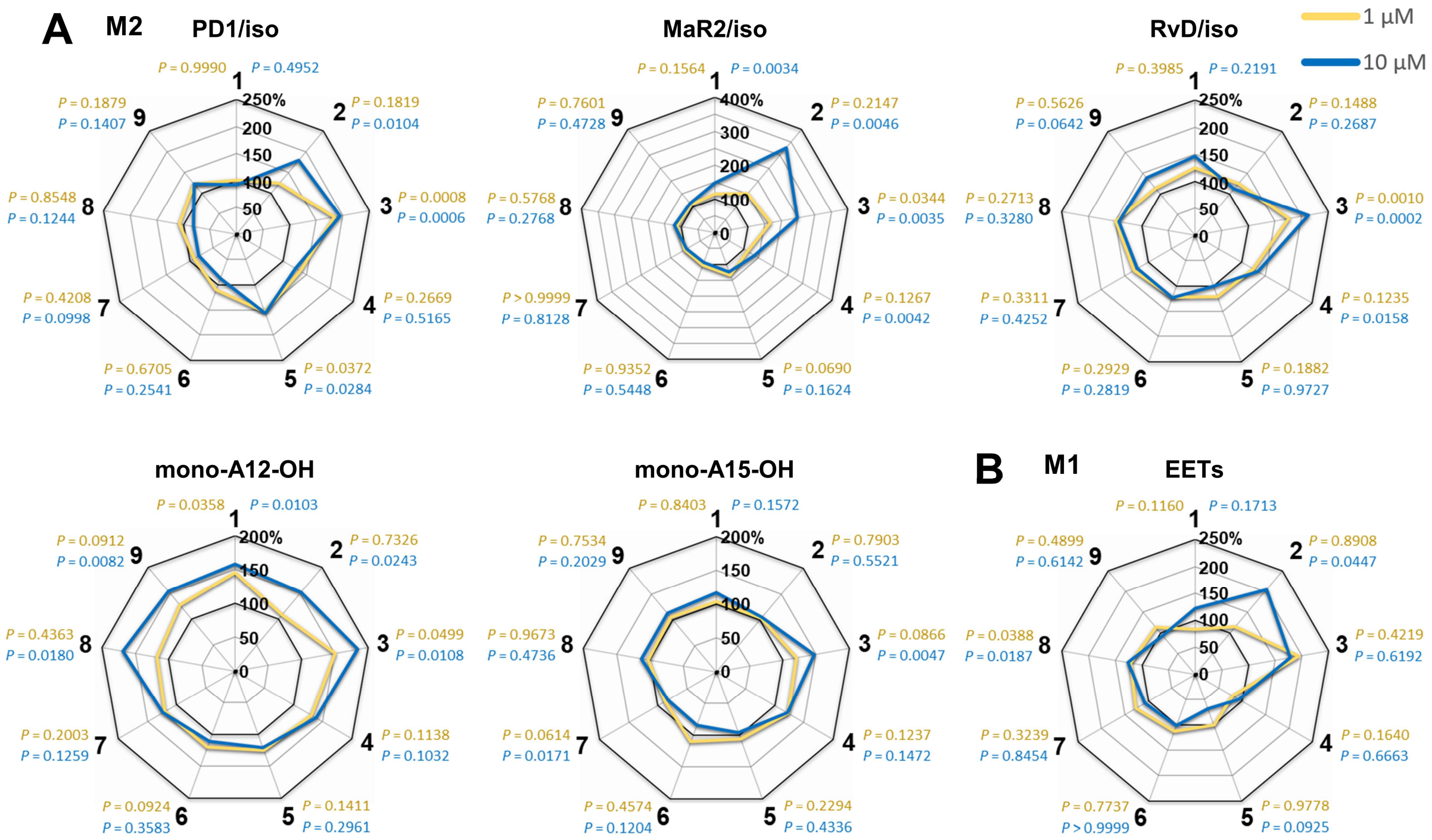

Figure S6. Effect of meroterpenoids on the biosynthesis of lipid mediator classes and selected species in activated macrophages. M1- or M2-like macrophages were preincubated (15 min) with vehicle (DMSO, 0.1%) or meroterpenoids and subsequently activated with SACM (180 min). A) PD1/iso, MaR2/iso, RvD/iso, mono-A12-OH, and mono-A15-OH produced by M2-like macrophages. Data for **1** are identical to Figure 2B-D. B) EETs produced by M1-like macrophages. Data are identical to Figure 3E. Mean from n = 3-4 independent experiments. *P* values given vs. vehicle control; repeated measures one-way ANOVA of log data or mixed-effects model (REML) of log data + Dunnett post hoc tests.

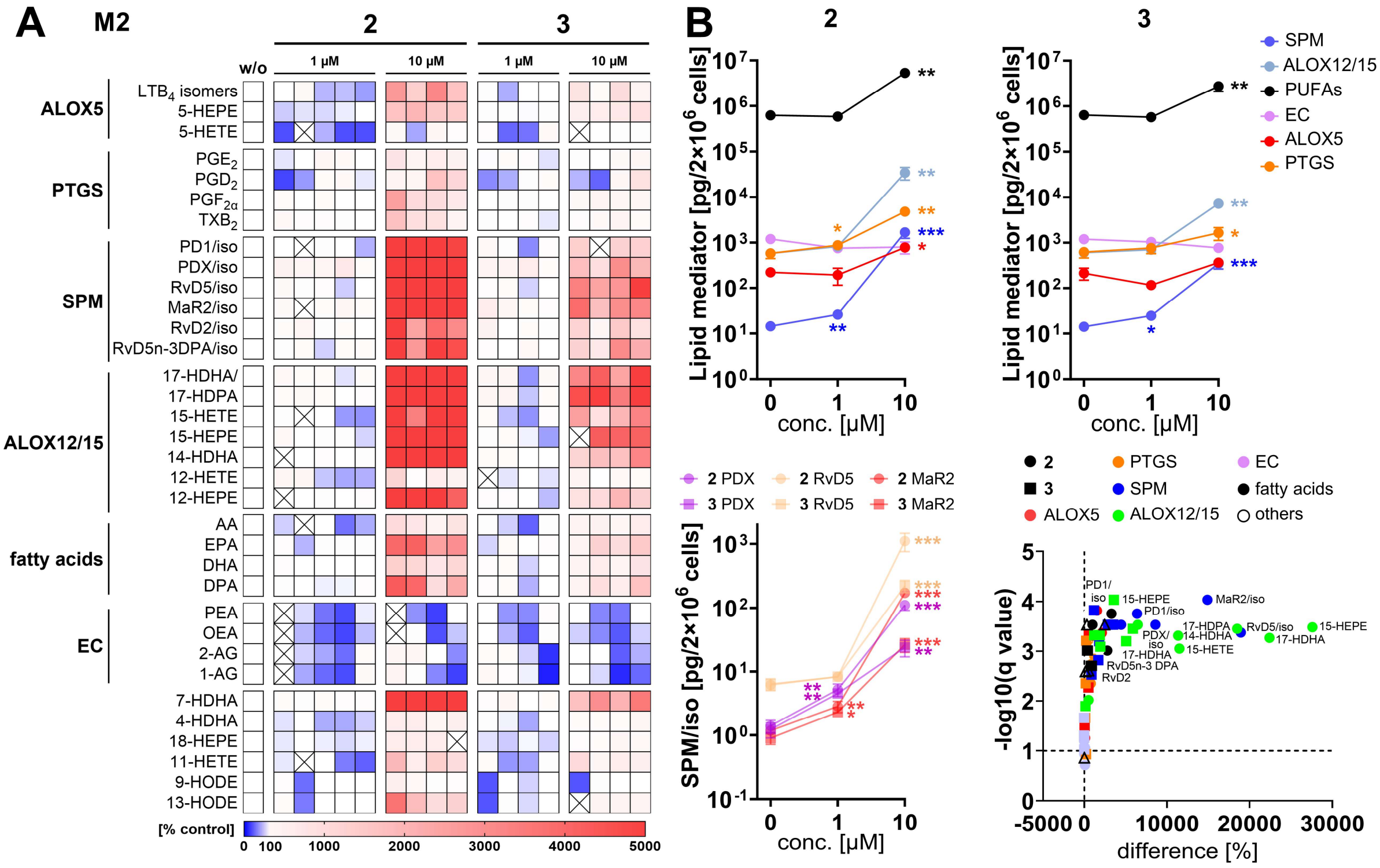

Figure S7. Effect of compounds **2** and **3** on the lipid mediator profile of resting M2-like macrophages. Cells were incubated with vehicle (DMSO, 0.1%), **2**, or **3** (10 µM unless stated otherwise) for 195 min**.** A) The heatmap depicts percentage changes in lipid mediator levels relative to vehicle control for each independent experiment; crossed sections indicate significant outliers. B) Line charts show the levels of lipid mediator subclasses or individual SPM/iso species, and the volcano plot shows the mean percentage difference relative to vehicle control and the negative $\log_{10}$(q value), calculated vs. vehicle control. Mean (A,B) or mean + SEM (B) from n = 3-5 independent experiments. *$P < 0.05$, **$P < 0.01$, ***$P < 0.001$ vs. vehicle control (B); two-tailed paired Student t test of log data (B). For the volcano plot (B), two-tailed multiple paired Student t tests with correction for multiple comparisons (false discovery rate 10%) were applied.

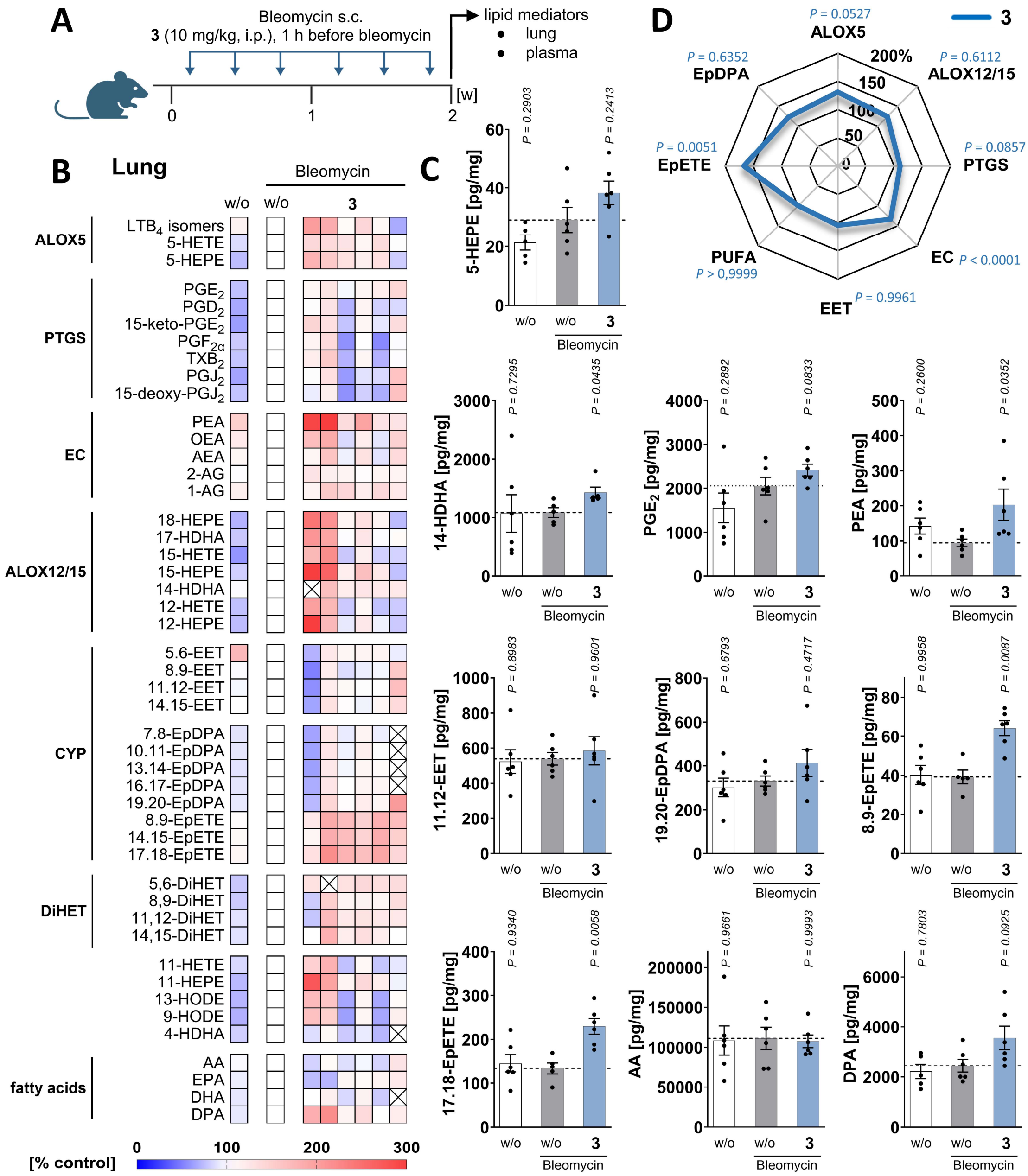

Figure S8. Effect of **2** and **3** on the lipid mediator profile of SACM-stimulated PBMCs. Cells were preincubated (15 min) with vehicle (DMSO, 0.1%), **2**, or **3** (10 µM) and subsequently activated with SACM (180 min)**.** A) The heatmap depicts percentage changes in lipid mediator levels relative to the SACM-treated vehicle control for each independent experiment; crossed sections indicate significant outliers. B) Levels of ALOX5 products, PTGS products, AEA, ALOX12/15 products, EETs, and AA/20:4 [per $1{\times}10^6$ cells]. Mean (A) or mean + SEM and single data (B) from n = 3-4 independent experiments. *P* values given vs. SACM-treated vehicle control; repeated measures one-way ANOVA of log data + Dunnett post hoc tests.

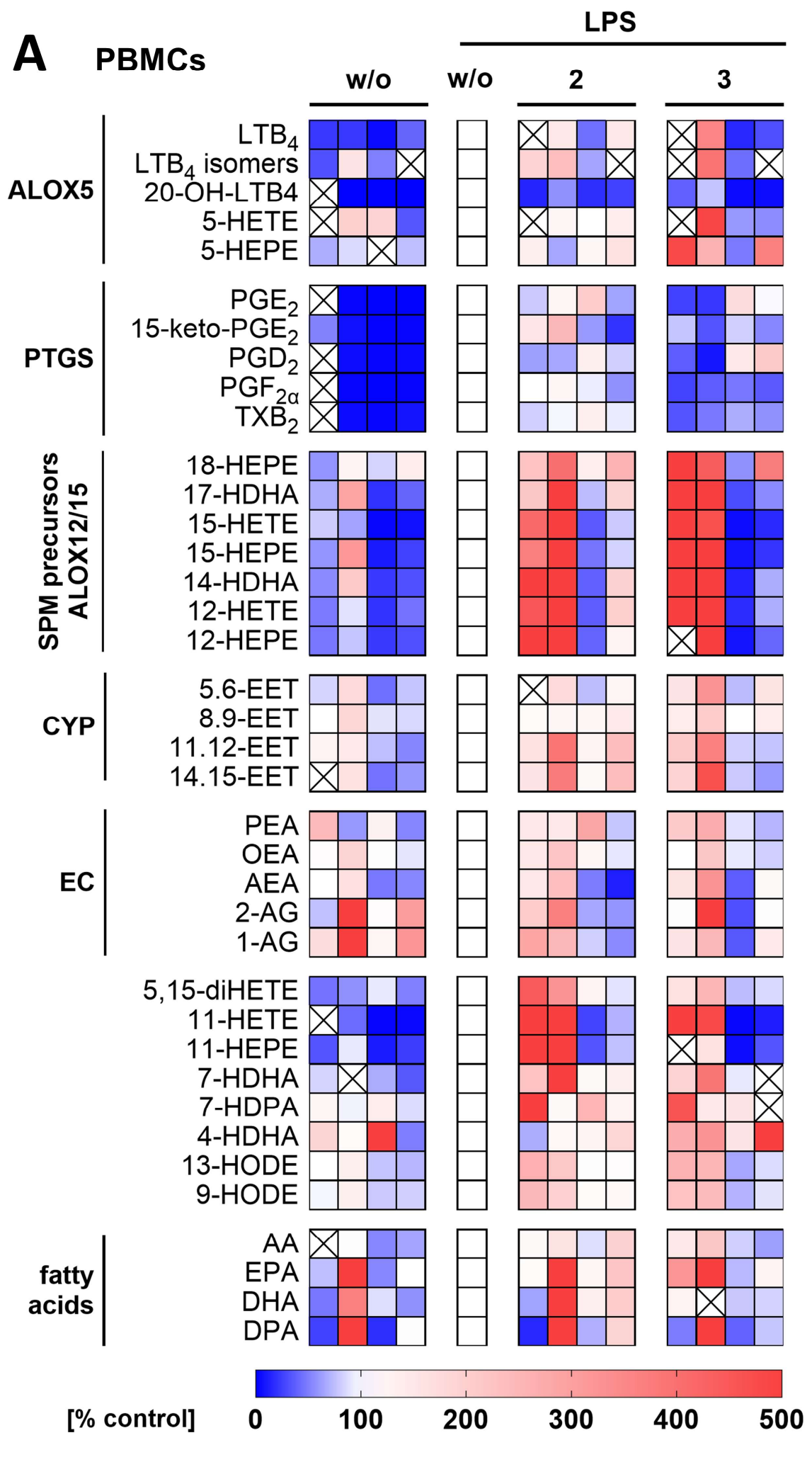

Figure S9. Effect of **2** and **3** on the lipid mediator profile of LPS-stimulated PBMCs. Cells were preincubated (15 min) with vehicle (DMSO, 0.1%), **2**, or **3** (10 µM) and subsequently stimulated with LPS (24 h)**.** A) The heatmap depicts percentage changes in lipid mediator levels relative to LPS-treated vehicle control for each independent experiment; crossed sections indicate significant outliers. B) Levels of 20-OH-LTB_4, PTGS products, $PGF_{2\alpha}$, ALOX12/15 products, EETs, and AA/20:4 [per 1×10^6 cells]. Mean (A) or mean + SEM and single data (B) from n = 3-4 independent experiments. *P* values given vs. LPS-treated vehicle control; repeated measures one-way ANOVA of log data + Dunnett post hoc tests.

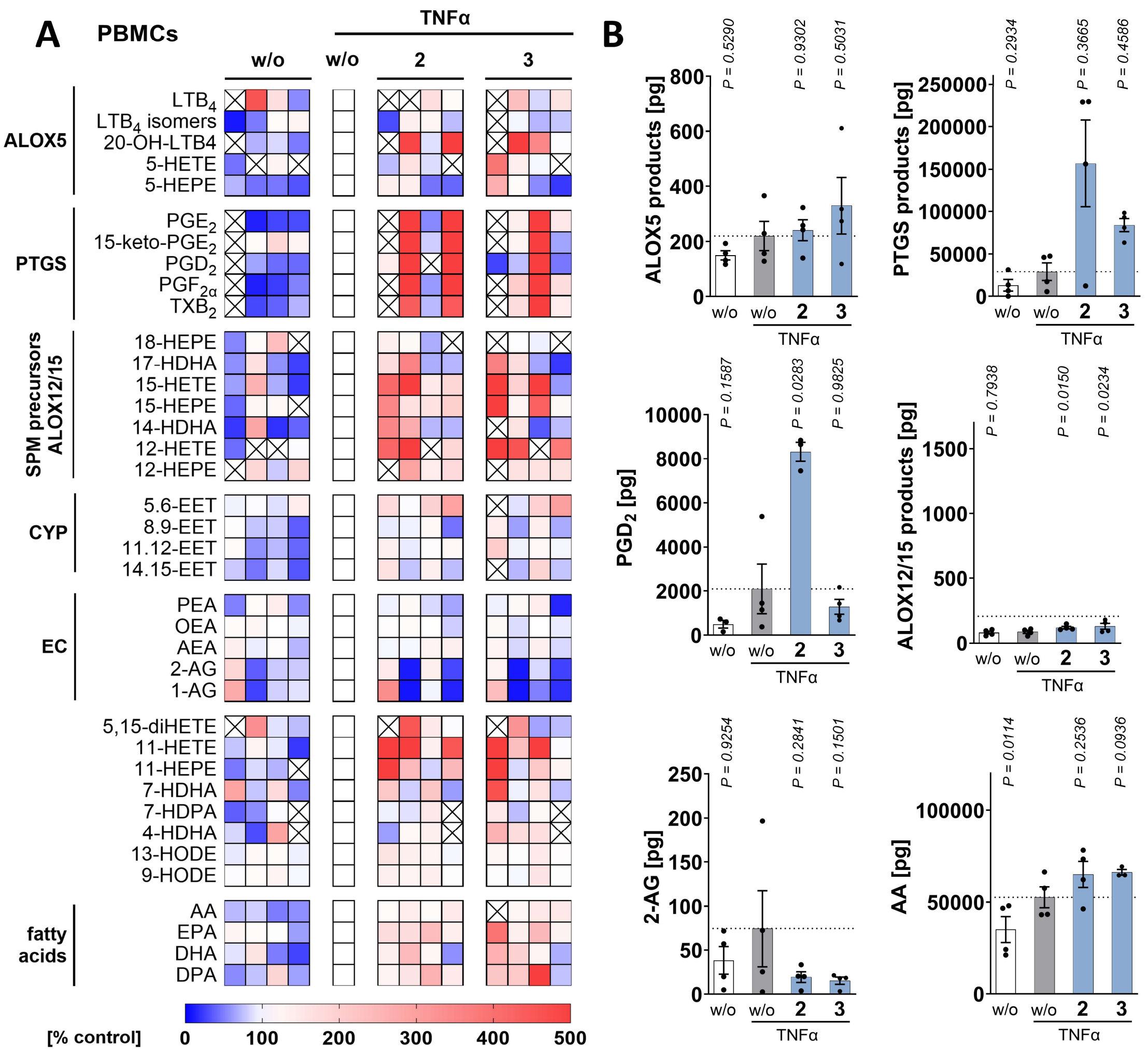

Figure S10. Effect of **2** and **3** on the lipid mediator profile of TNF-α-stimulated PBMCs. Cells were preincubated (15 min) with vehicle (DMSO, 0.1%), **2**, or **3** (10 µM) and subsequently stimulated with TNF-α (24 h)**.** A) The heatmap depicts percentage changes in lipid mediator levels relative to the TNF-α-treated vehicle control for each independent experiment; crossed sections indicate significant outliers. B) Levels of ALOX5 products, PTGS products, PGD_2, ALOX12/15 products, 2-AG, and AA/20:4 [per 1×10^6 cells]. Mean (A) or mean + SEM and single data (B) from n = 2-4 independent experiments. *P* values given vs. TNF-α-treated vehicle control; repeated measures one-way ANOVA of log data + Dunnett post hoc tests.

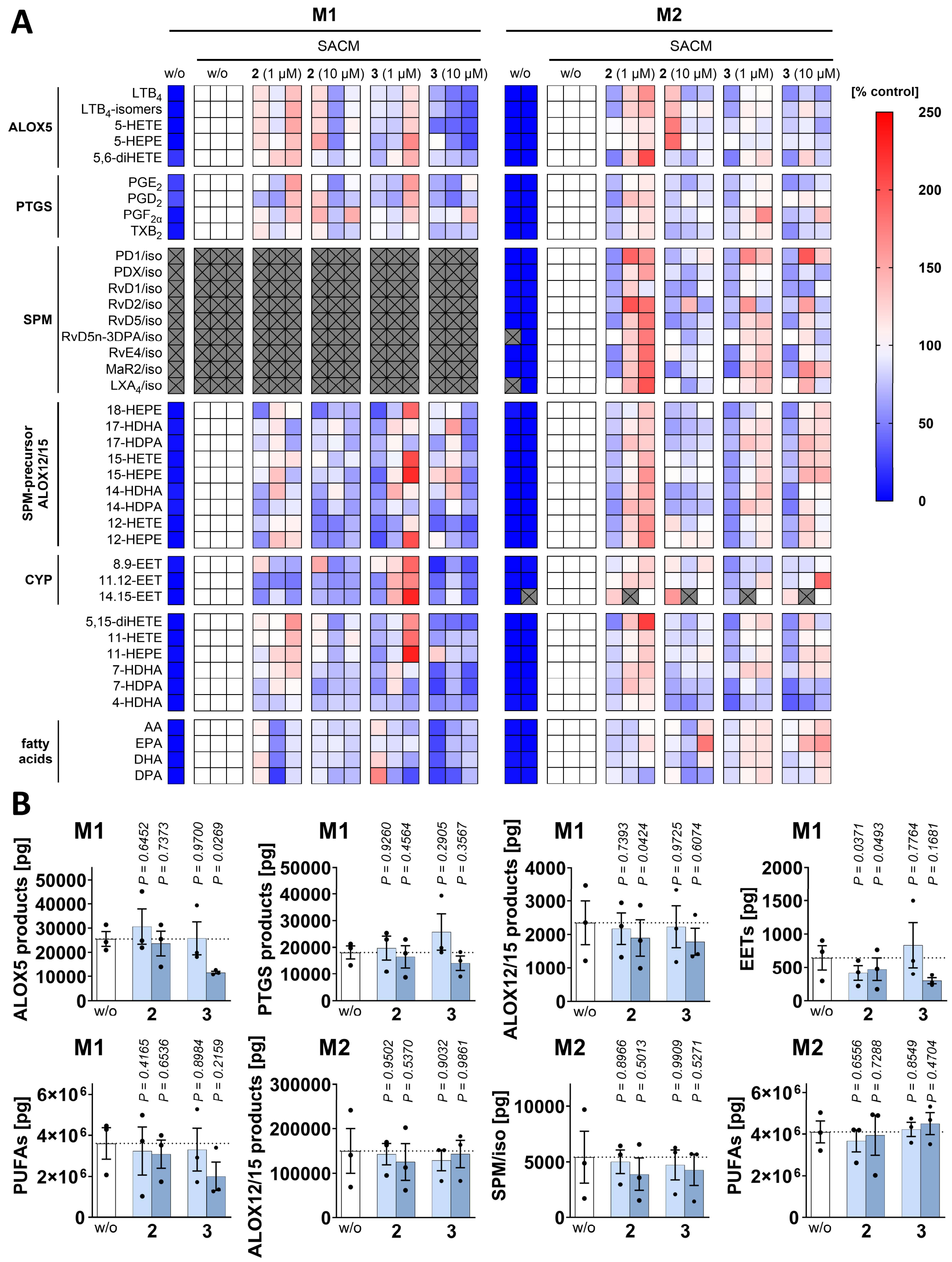

Figure S11. Effect of **2** and **3** during polarization to M1- and M2-like macrophages on the capacity to produce lipid mediators. Monocyte-derived macrophages were treated with vehicle (DMSO, 0.1%), **2**, or **3** (1 or 10 µM) during polarization for 48 h with either LPS and interferon-γ (M1-like macrophages) or interleukin-4 towards (M2-like macrophages). After polarization, the capacity to produce lipid mediators was assessed by stimulating cells with SACM (1%, 180 min). A) The heatmap depicts percentage changes in lipid mediator levels relative to the SACM-treated vehicle control for each independent experiment; crossed sections indicate significant outliers (M2) or lipid mediators not detected (M1). B) Levels of ALOX5 products, PTGS products, ALOX12/15 products, EETs, PUFAs, and SPM/iso in M1- and/or M2-like macrophages [per 1×10^6 cells]. Mean (A) or mean + SEM and single data (B) from n = 1 (A - w/o, M1), n = 2 (A - w/o, M2), or n = 2-3 independent experiments. *P* values given vs. SACM-treated vehicle control; repeated measures one-way ANOVA of log data + Dunnett post hoc tests.

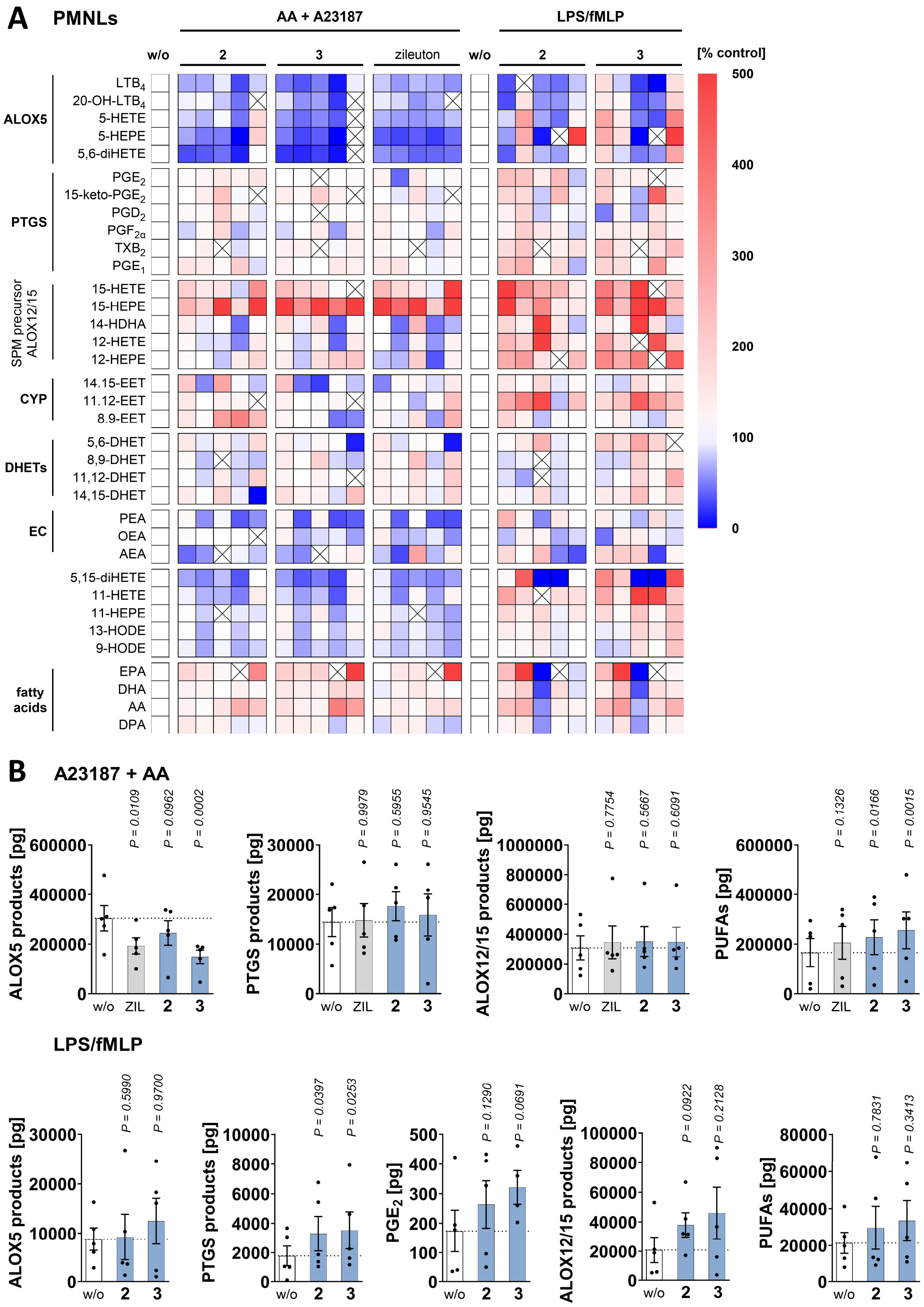

Figure S12. Effect of **2** and **3** on the lipid mediator profile of activated PMNL. Cells were preincubated (10 min) with vehicle (DMSO, 0.1%), **2**, or **3** (10 µM) and subsequently treated with A23187 and 20:4/AA (10 min). Alternatively, cells were first primed with LPS (10 min), treated with vehicle, **2**, or **3**, and stimulated with fMLP (5 min)**.** A) The heatmap depicts percentage changes in lipid mediator levels relative to the A23187- and AA/20:4- or LPS/fMLP-treated vehicle control for each independent experiment; crossed sections indicate significant outliers. B) Levels of ALOX5 products, PTGS products, PGE_2, ALOX12/15 products, and PUFAs [per $5{\times}10^6$ cells]. Mean (A) or mean + SEM and single data (B) from n = 4-5 independent experiments. *P* values given vs. TNF-α-treated vehicle control; repeated measures one-way ANOVA of log data + Dunnett post hoc tests.

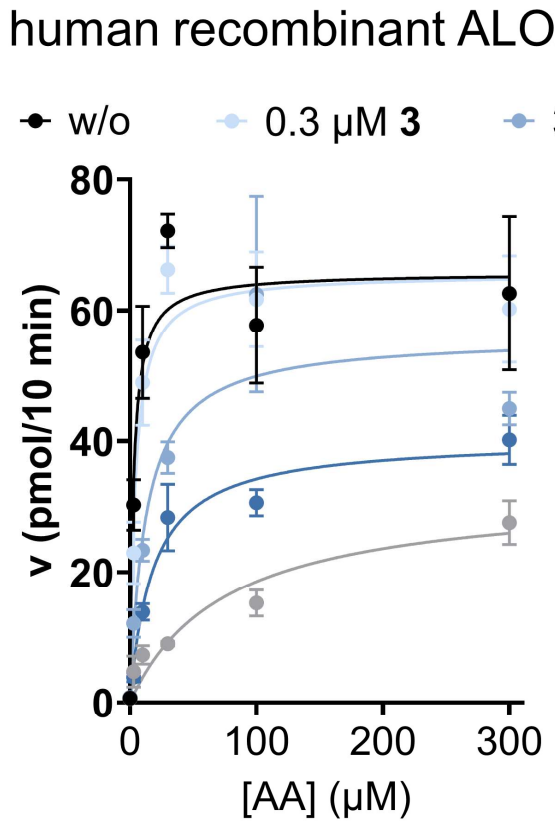

	apparent K_M (µM)	apparent V_{max} (pmol/min)
vehicle	2.8	65.8
**3** (0.3 µM)	4.1	65.7
**3** (3 µM)	12.3	56.1
**3** (30 µM)	18.0	40.4
Zileuton	75.1	32.5

Figure S13. Mixed-type ALOX5 inhibition by meroterpenoid **3**. Human recombinant ALOX5 was preincubated (10 min) with vehicle (DMSO, 1%), **3** at the indicated concentrations, or zileuton (3 µM), and the reaction was initiated with varying concentrations of AA/20:4 (10 min). Mean + SEM from n = 5 independent experiment*s*. Apparent K_M and V_{max} values were determined by nonlinear curve fitting using the Michaelis-Menten equation.

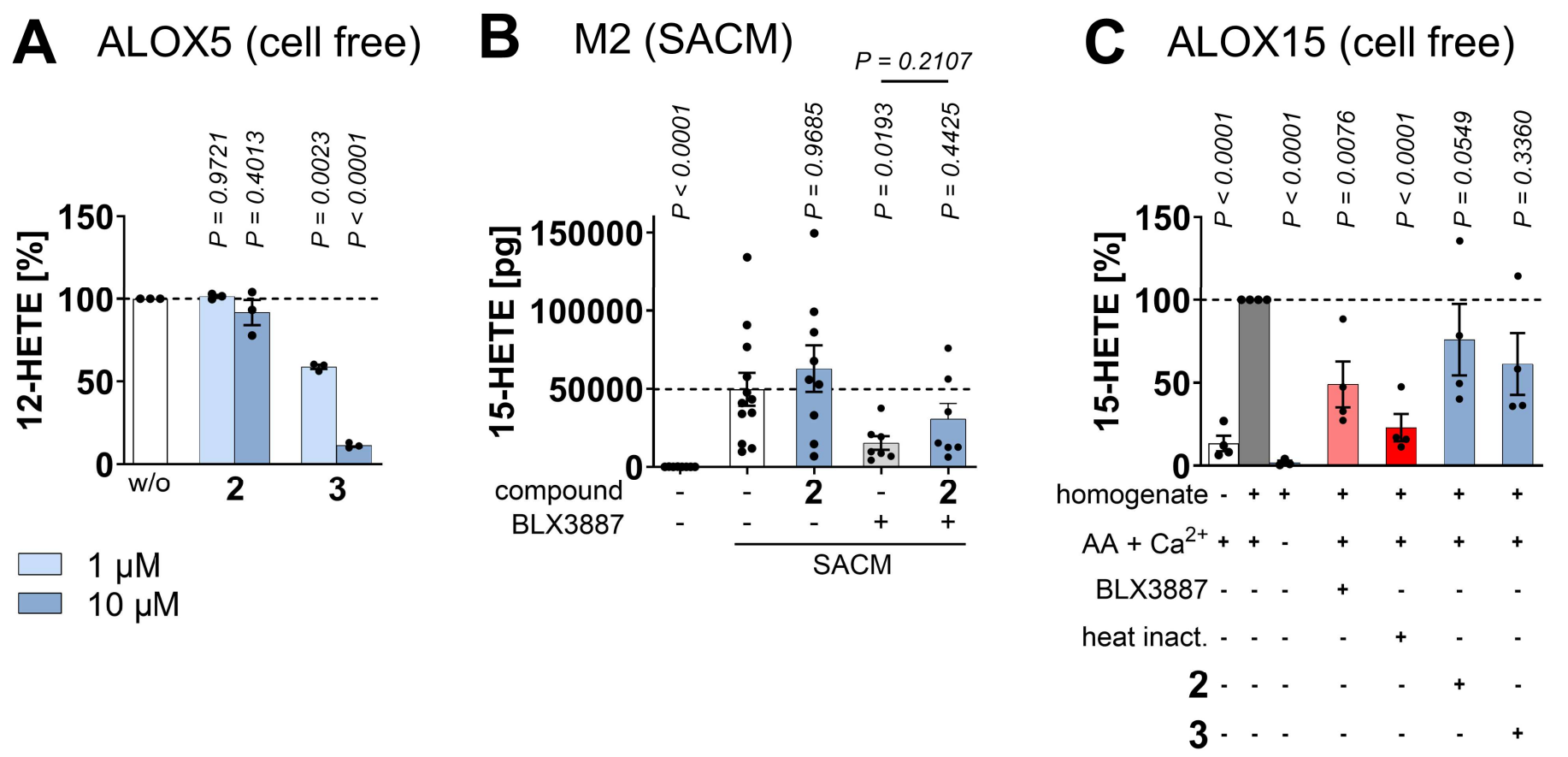

Figure S14. Effect of **2** and **3** on positional side reactions of human ALOX5 and ALOX15 activity. A) Human recombinant ALOX5 was pretreated (10 min) with vehicle (DMSO, 0.1%), **2**, or **3**, and product formation was initiated with AA/20:4 and $CaCl_2$ (10 min). Shown is the conversion of AA/20:4 to 12-HETE by ALOX5. B) 15-HETE formation by M1- or M2-like macrophages preincubated (15 min) with vehicle (DMSO, 0.1%), BLX3887 (0.5-1 µM), or **2** (10 µM) and activated with SACM (180 min) [per 2×10^6 cells]. C) 15-HETE formation (indicative of ALOX15 activity) in M2 homogenates preincubated (15 min) with vehicle (DMSO, 0.1%), **2** (10 µM), **3** (10 µM), or BLX3887 (0.2 µM) and then treated with AA/20:4 and $CaCl_2$ (15 min). Mean + SEM and single data from n = 3 (A), n = 7-12 (B), n = 3-4 (C) independent experiments. *P* values given vs. vehicle control; repeated measures one-way ANOVA of log data (A) or mixed-effects model (REML) of log data (B,C) + Dunnett post hoc tests.

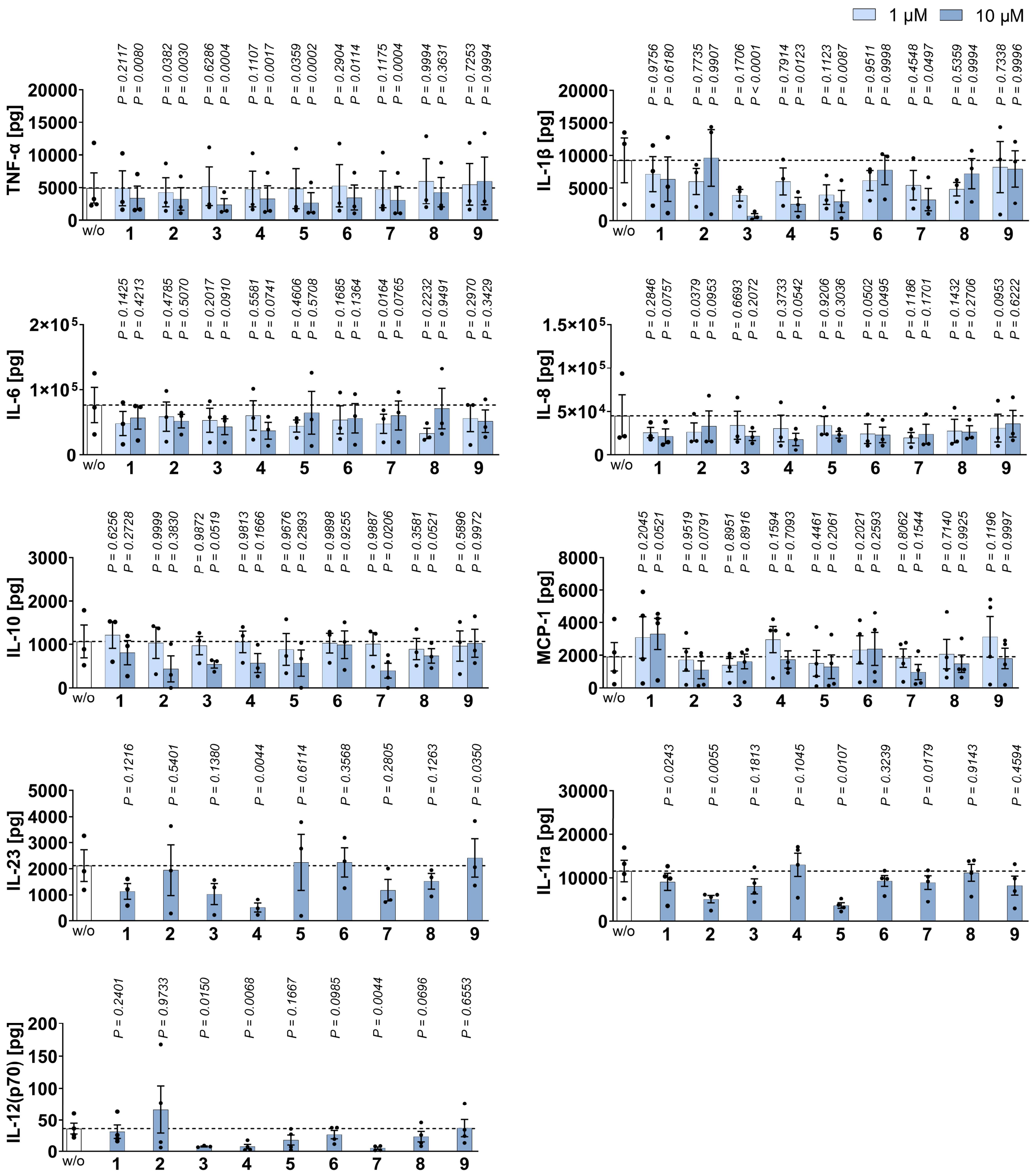

Figure S15. Effect of meroterpenoids on cytokine and chemokine expression in PBMCs. Cells were pretreated (30 min) with vehicle (DMSO, 0.1 %) or meroterpenoids, followed by stimulation with LPS for 4 h (TNF-α, IL-8) or 18 h (IL-1β, IL-6, IL-10, MCP-1, IL-12 (p70), IL-23, IL-1ra). Cytokine and chemokine levels are given as pg per 1.4×10^6 PBMCs. Data for TNF-α and IL-1β are identical to Figure 6C. Mean + SEM and single data from n = 3-4 independent experiments. *P* values given vs. vehicle control; repeated measures one-way ANOVA of log data (IL-1β, IL-6, IL-8, IL-10, MCP-1) or mixed-effects model (REML) of log data (TNF-α) + Dunnett *post hoc* tests or two-tailed paired student *t* tests of log data (IL-23, IL-1ra, IL-12(p70).

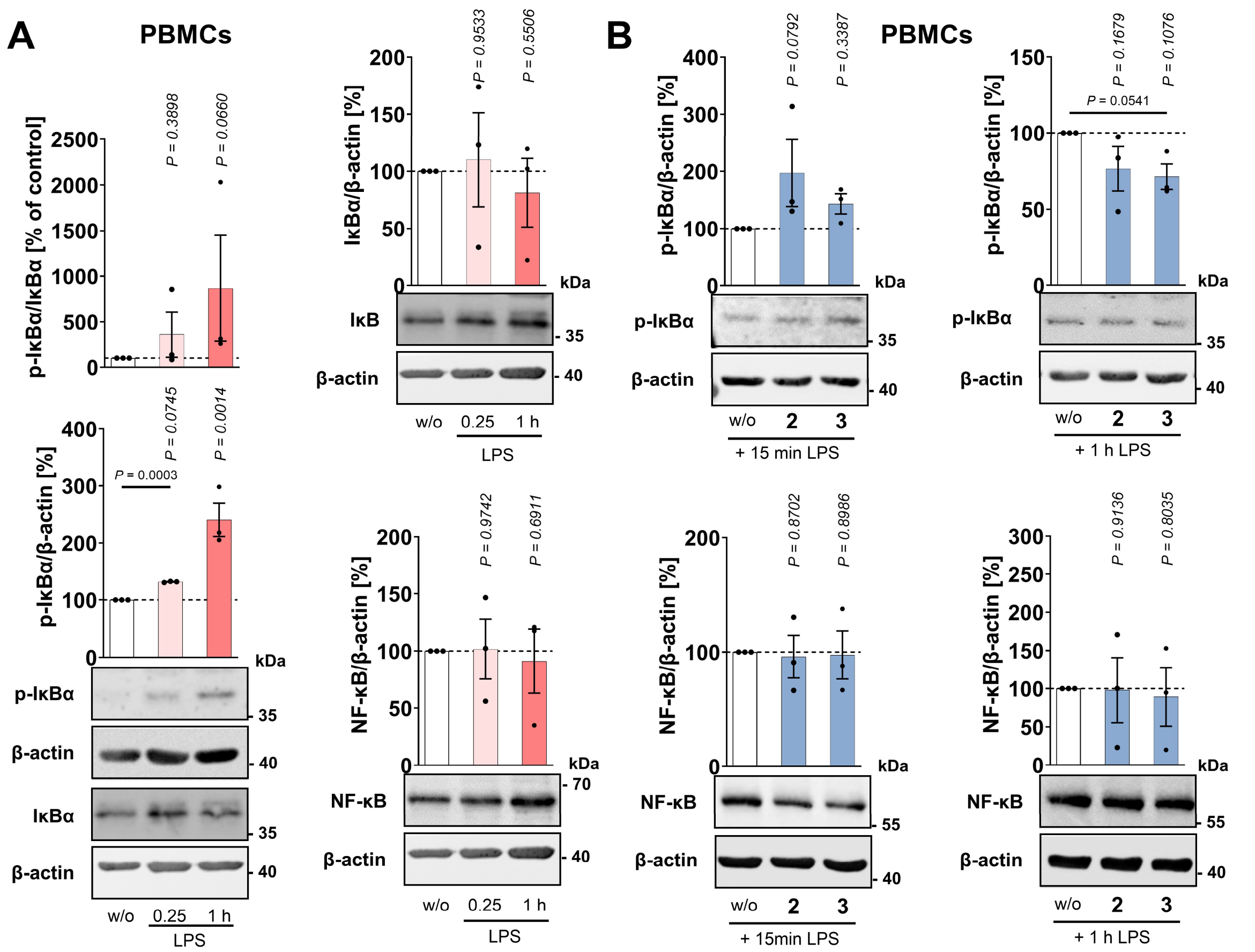

Figure S16. Effect of LPS, **2** and **3** on IκBα phosphorylation and IκBα and NF-κB expression. PBMCs were pretreated (30 min) with vehicle (DMSO, 0.1 %), **2**, or **3** (10 µM) and subsequently stimulated with LPS for 15 min or 1 h. A) Changes in IκBα phosphorylation and IκBα and NF-κB protein levels. Western blots are representative of three independent experiments. Uncropped blots are shown in Figure S17C, Supporting Information. B) Changes in p-IκB and NF-κB protein levels. Blots shown are identical to those shown in Figure 6B, and the vehicle controls are identical to Waltl et al.[1]. Uncropped blots are shown in Figure S17A,B, Supporting Information. Mean + SEM and single data from n = 3 independent experiments. *P* values given vs. vehicle control; repeated measures one-way ANOVA (B) of log data (A) + Dunnett post hoc tests and two-tailed paired student *t* test for pairwise comparisons as indicated by bars (A,B).

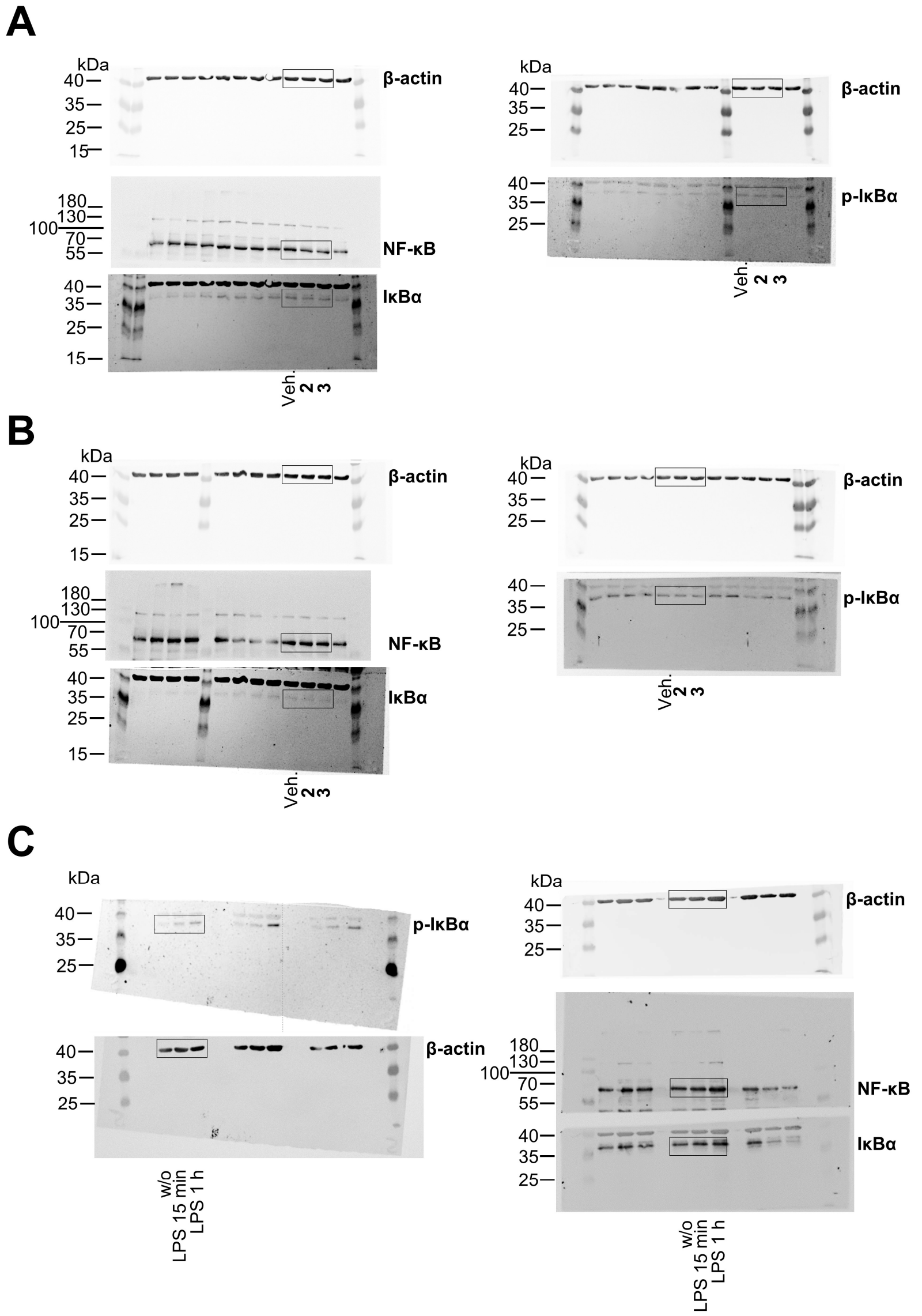

Figure S17. Uncropped Western blots corresponding to Figure 6B and Figure S16, Supporting Information. A,B) Effect of vehicle, **2**, and **3** on NFκB, IκB, p-IκB, and β-actin protein levels in PBMCs stimulated with LPS for 15 min (A) or 1 h (B). The vehicle controls are identical to Waltl et al.[1] C) Time-dependent effect of LPS on the levels of NFκB, IκB, and p-IκB.

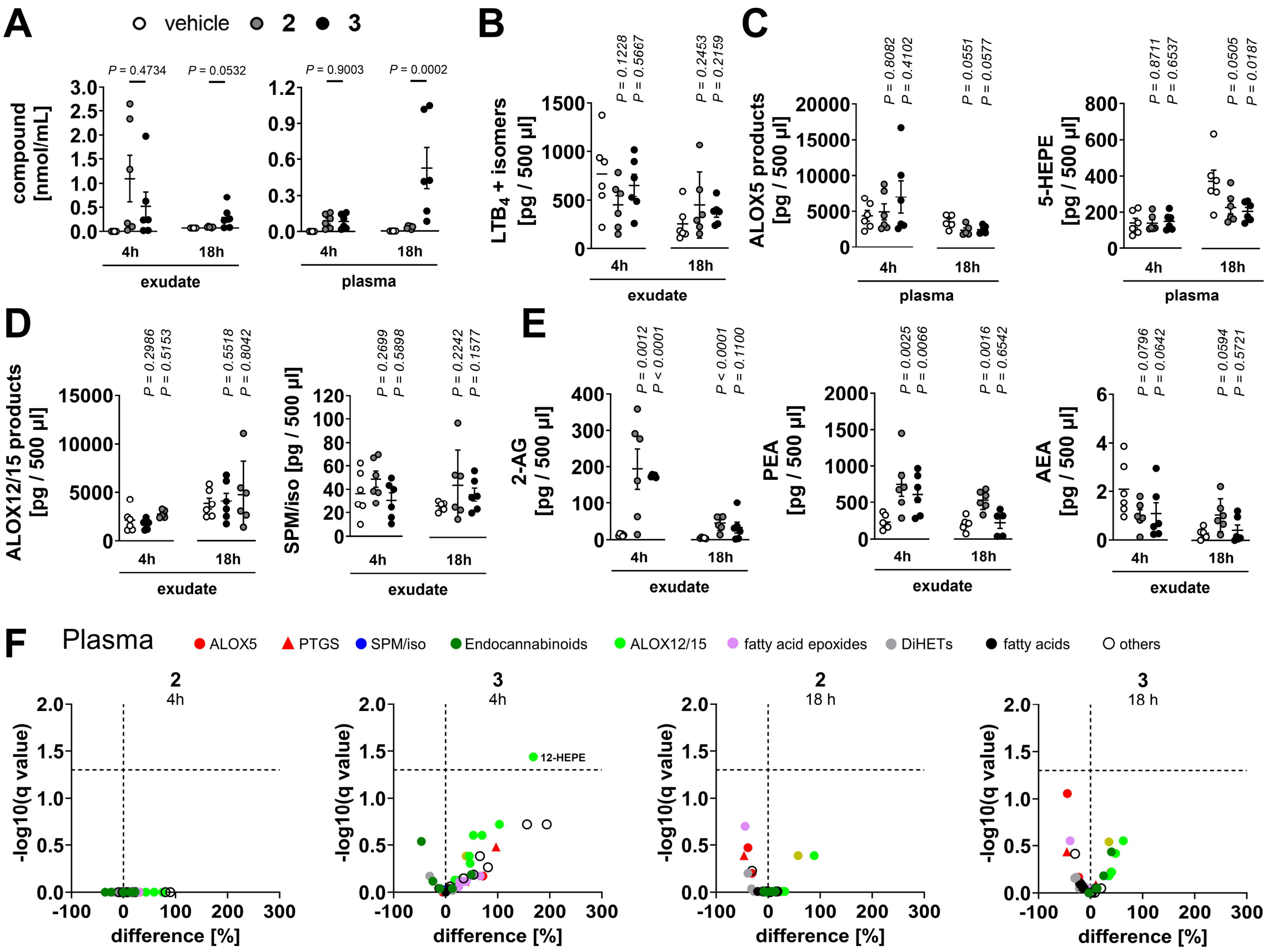

Figure S18. Exudate and plasma concentrations of **2** and **3** in murine peritonitis and effects on systemic and local lipid mediator profiles. Mice received vehicle (DMSO), **2**, or **3** (10 mg/kg, i.p.) 30 min prior to zymosan injection and were sacrificed after 4 h or 18 h for the analysis of the administered compounds and lipid mediators in plasma and peritoneal exudates. A) Concentrations of **2** or **3** in exudates and plasma. B) Total LTB_4 plus LTB_4 isomer levels in exudates. C) ALOX5 product and 5-HETE levels in plasma. D) ALOX12/15 product and SPM/iso levels in exudates. E) Levels of selected endocannabinoids in exudates. F) Volcano plots depict the mean percentage difference relative to vehicle control and the negative $\log_{10}$(q value), calculated vs. vehicle control. Mean (F) or mean + SEM and single data (A-E) from n = 5-6 (C,E,F), n = 6 (A,B,D). P values given vs. vehicle control; two-tailed unpaired student t test (B,C,D) of log data (A,E). For volcano plots (F), two-tailed multiple paired Student t tests with correction for multiple comparisons (false discovery rate 5%) were applied.

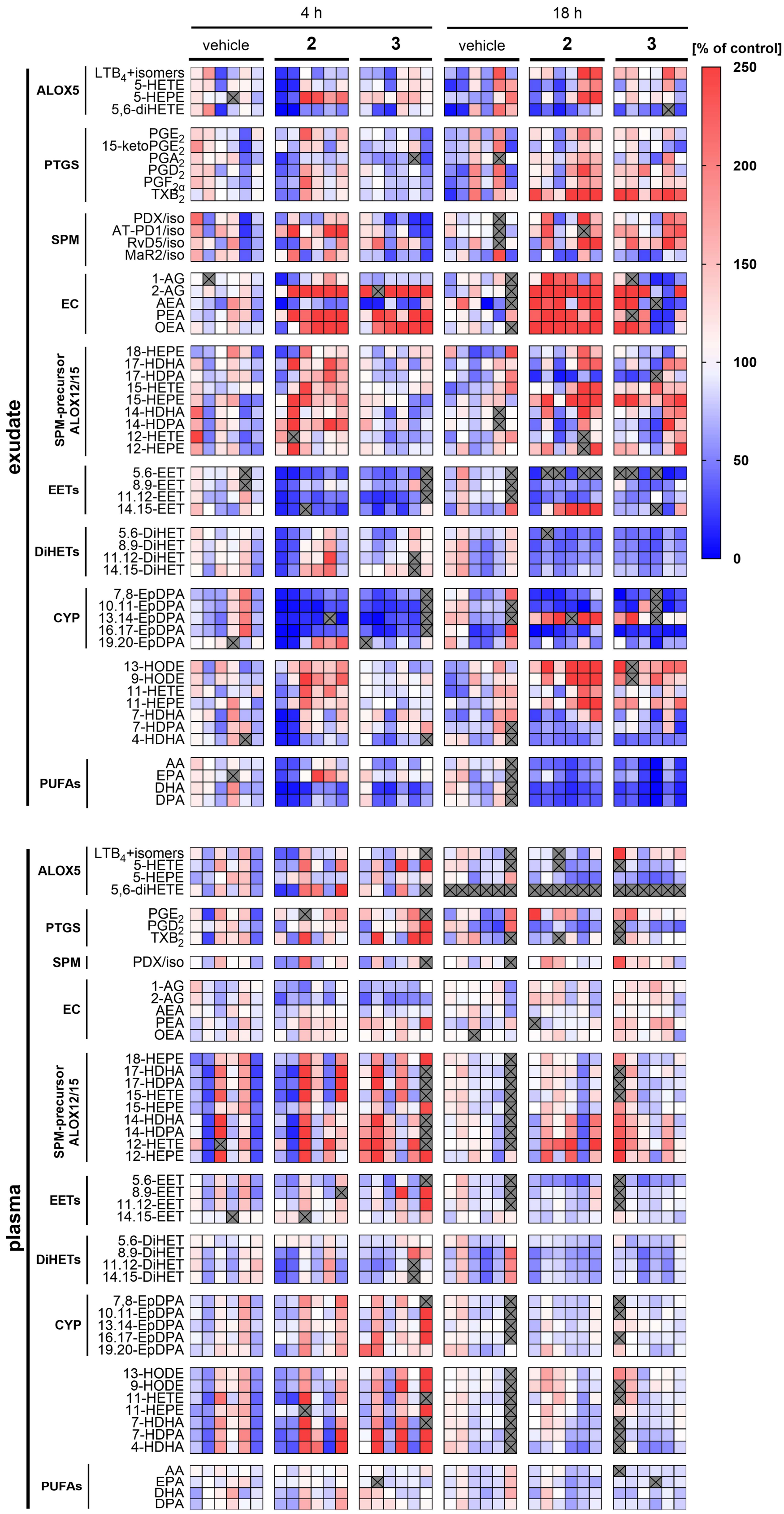

Figure S19. Detailed lipid mediator profile of peritoneal exudates and plasma from zymosan-challenged mice upon administration of **2** or **3**. Mice received vehicle (DMSO), **2**, or **3** (10 mg/kg, i.p.) 30 min prior to zymosan injection and were sacrificed after 4 h or 18 h for the analysis of lipid mediators in plasma and exudate. The heatmap depicts percentage changes in lipid mediator levels relative to vehicle control for each individual animal

(n = 2-6); crossed sections indicate significant outliers or lipid mediators that were not detected for analytical reasons.

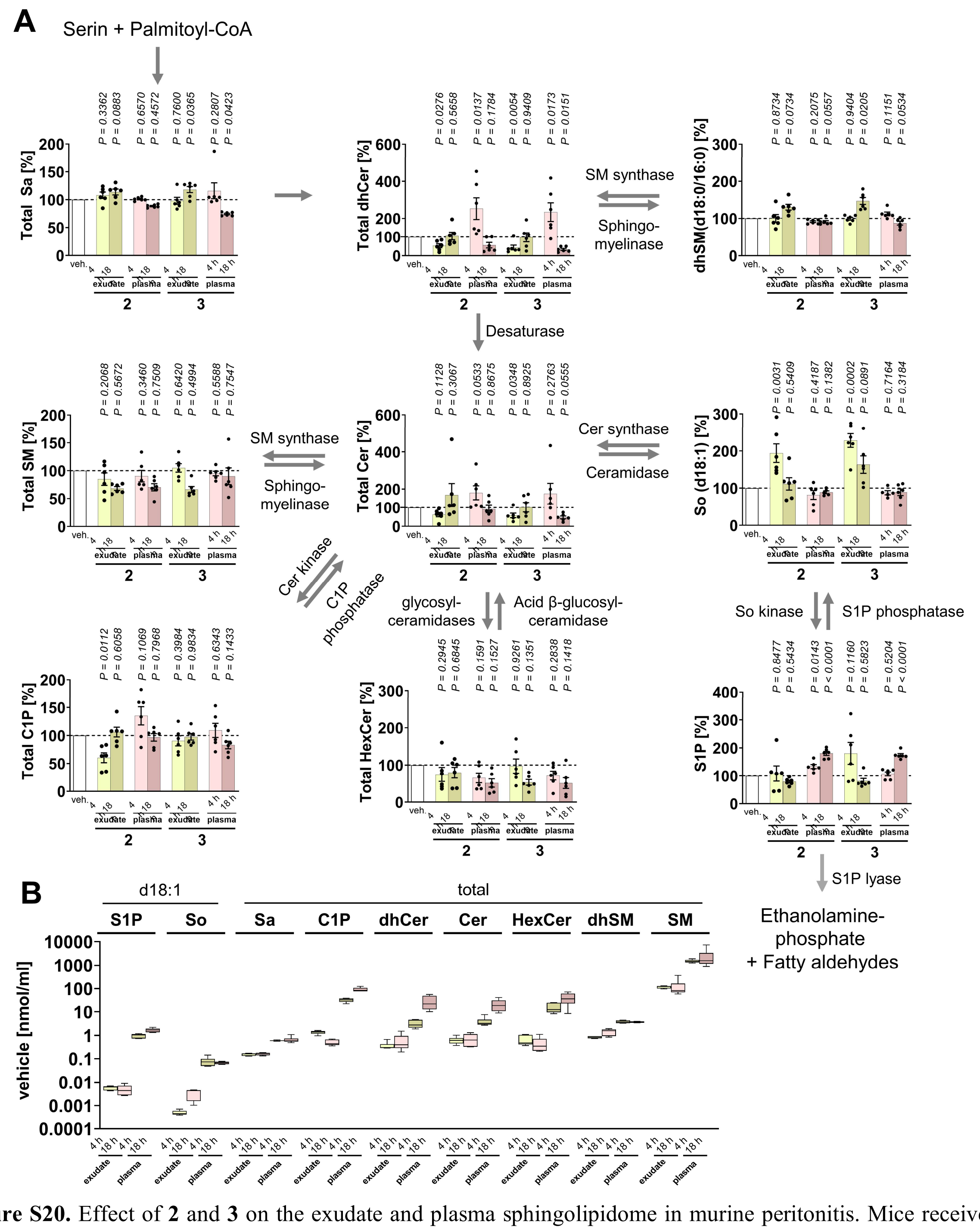

Figure S20. Effect of **2** and **3** on the exudate and plasma sphingolipidome in murine peritonitis. Mice received vehicle (2% DMSO in saline; 0.5 mL), **2**, or **3** (10 mg/kg, i.p.) 30 min prior to zymosan injection and were sacrificed after 4 h or 18 h for the analysis of sphingolipids in plasma and exudate**.** A) Percentage changes in the levels of sphingolipid subclasses relative to vehicle control and visualization of interconverting metabolic pathways and enzymes. Data for S1P are identical to Figure 7G. Abbreviations: Ceramide-1-phosphate (C1P), (dihydro)ceramide ((dh)Cer), (dihydro)sphingomyelin ((dh)SM), hexosylceramides (HexCer), sphingosine-1-phosphate (S1P), sphinganine (Sa), sphingosine (So). Mean + SEM and single data from n = 5-6 mice. *P* values given vs. vehicle control; two-tailed unpaired Student *t* test of log data. B) Comparison of absolute concentrations of sphingolipid classes in vehicle control. Mean + SEM from n = 5-6 mice.

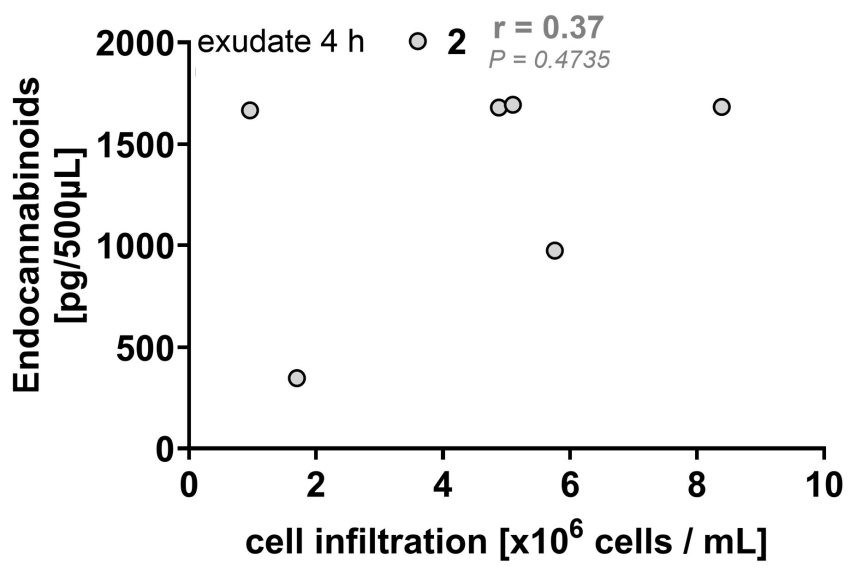

Figure S21. Endocannabinoid levels in the peritoneal exudate of **2**-treated mice do not correlate with cell infiltration in peritonitis. Pearson correlation between peritoneal cell infiltration and total endocannabinoid concentrations, calculated from mean cell numbers and mean endocannabinoid levels. Mean from n = 6 mice.

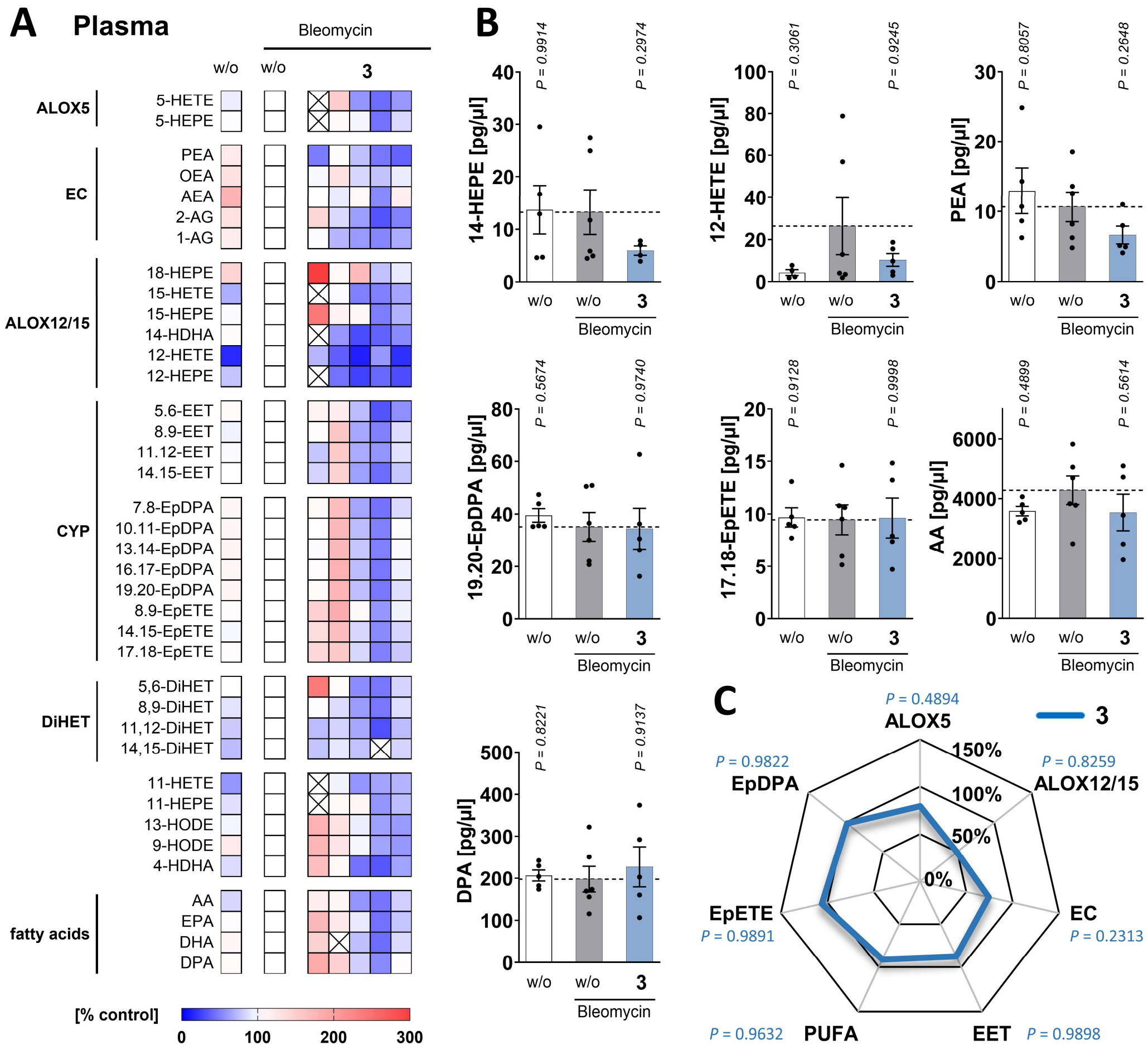

Figure S22. Limited effect of meroterpenoid **3** on circulating lipid mediator profiles. Mice received vehicle (1% DMSO in saline; 0.5 mL) or **3** (10 mg/kg, i.p.) 1 h before bleomycin or mock injection three times a week over 2 weeks. Mice were sacrificed within 48 h after the last administration, and the lipid mediator profile of plasma was analyzed. A) The heatmap depicts percentage changes in lipid mediator concentrations relative to vehicle control; crossed sections indicate significant outliers. B) Levels of 14-HDHA, 12-HETE, PEA, 19,20-EpDPA, 17,18-EpETE, AA/20:4, and 22:5/DPA [per µL plasma]. C) Percentage changes in the concentrations of ALOX5 products, ALOX12/15 products, endocannabinoids (EC), EETs, EpETEs, EpDPAs, and PUFAs. Mean (A-w/o and w/o + Bleomycin, C), individual values (A-compound **3**), or mean + SEM and single data (B) from n = 4-6 mice. *P* values given vs. bleomycin/vehicle group (B, C); ordinary one-way ANOVA of log data + Dunnett *post hoc* tests. The lipid mediators belonging to each subclass are defined in panel A.

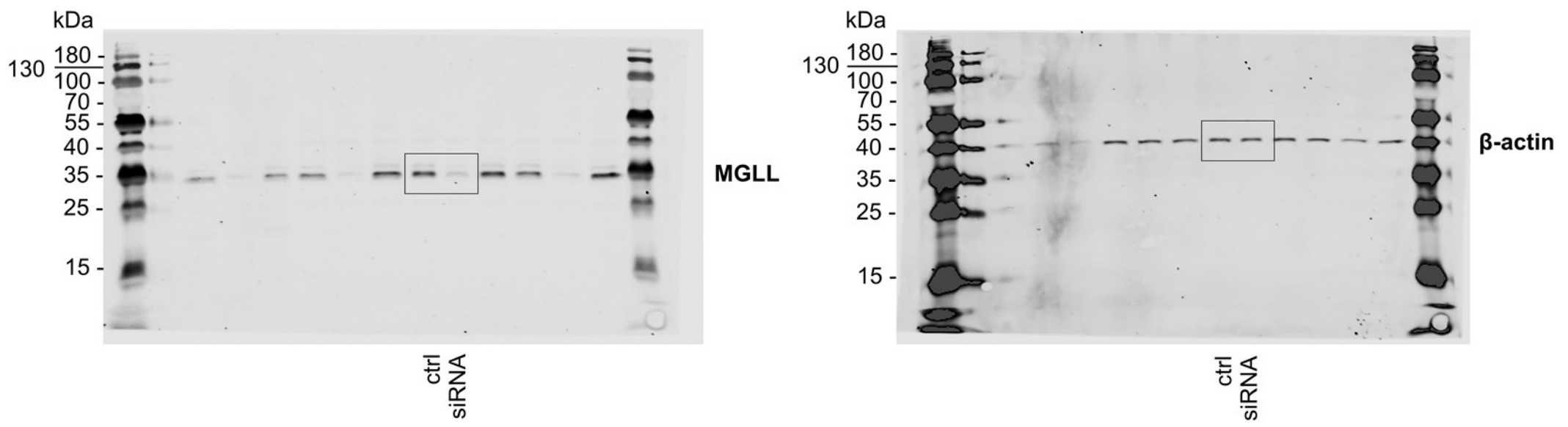

Figure S23. Uncropped Western blots corresponding to Figure 9B. MGLL and β-actin protein levels in MCF7 cells transfected with control siRNA or *MGLL* siRNA.

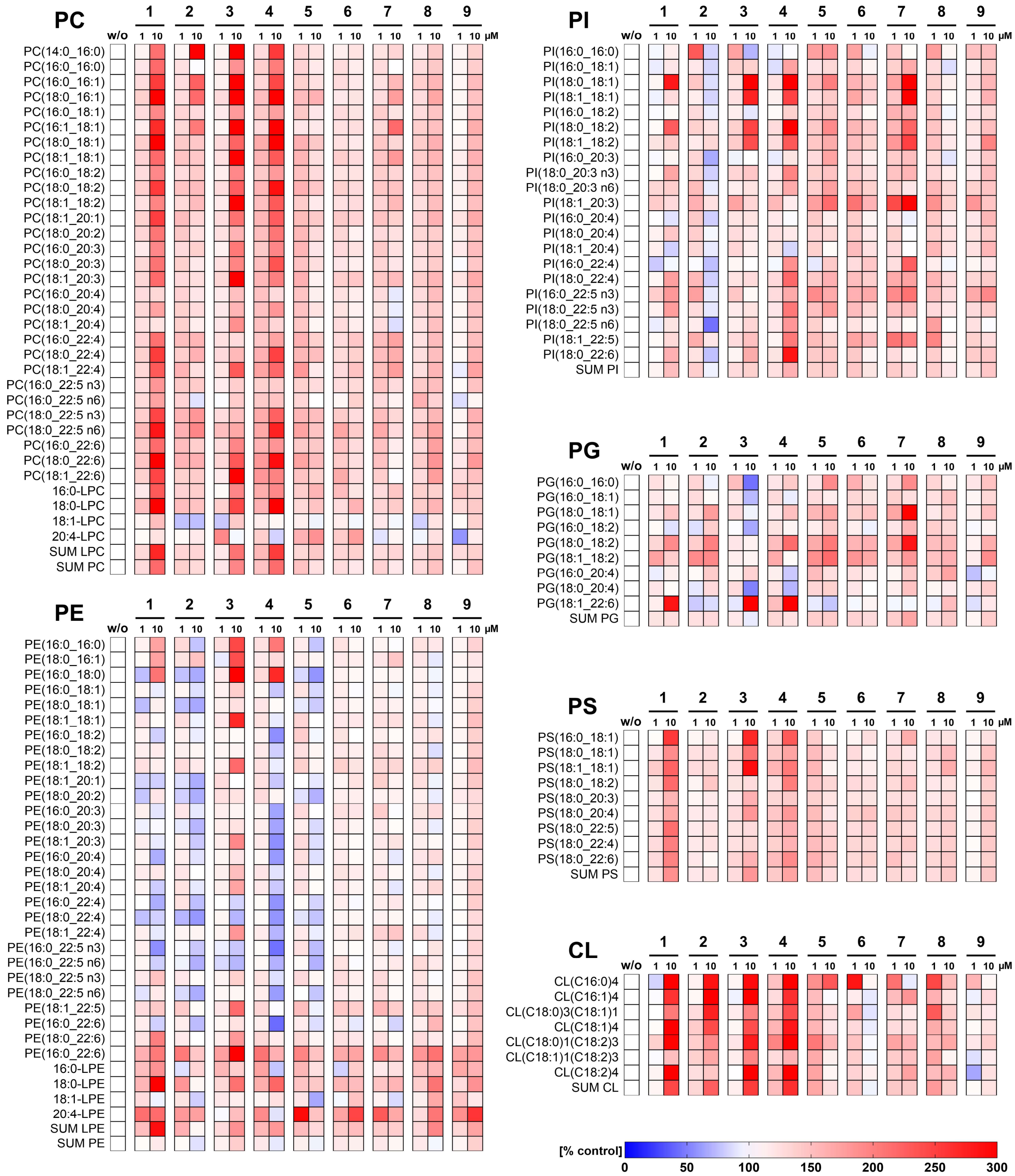

Figure S24. Effect of meroterpenoids on the glycerophospholipidome of PBMCs. Cells were incubated with vehicle (DMSO, 0.1%) or meroterpenoids for 48 h. Heatmaps depict percentage changes in the concentrations of individual glycerophospholipid species and the subclass sum (‘SUM’) relative to vehicle control. Data are given as mean from n = 3 independent experiments. Data for subclass sums are identical to Figure 10B.

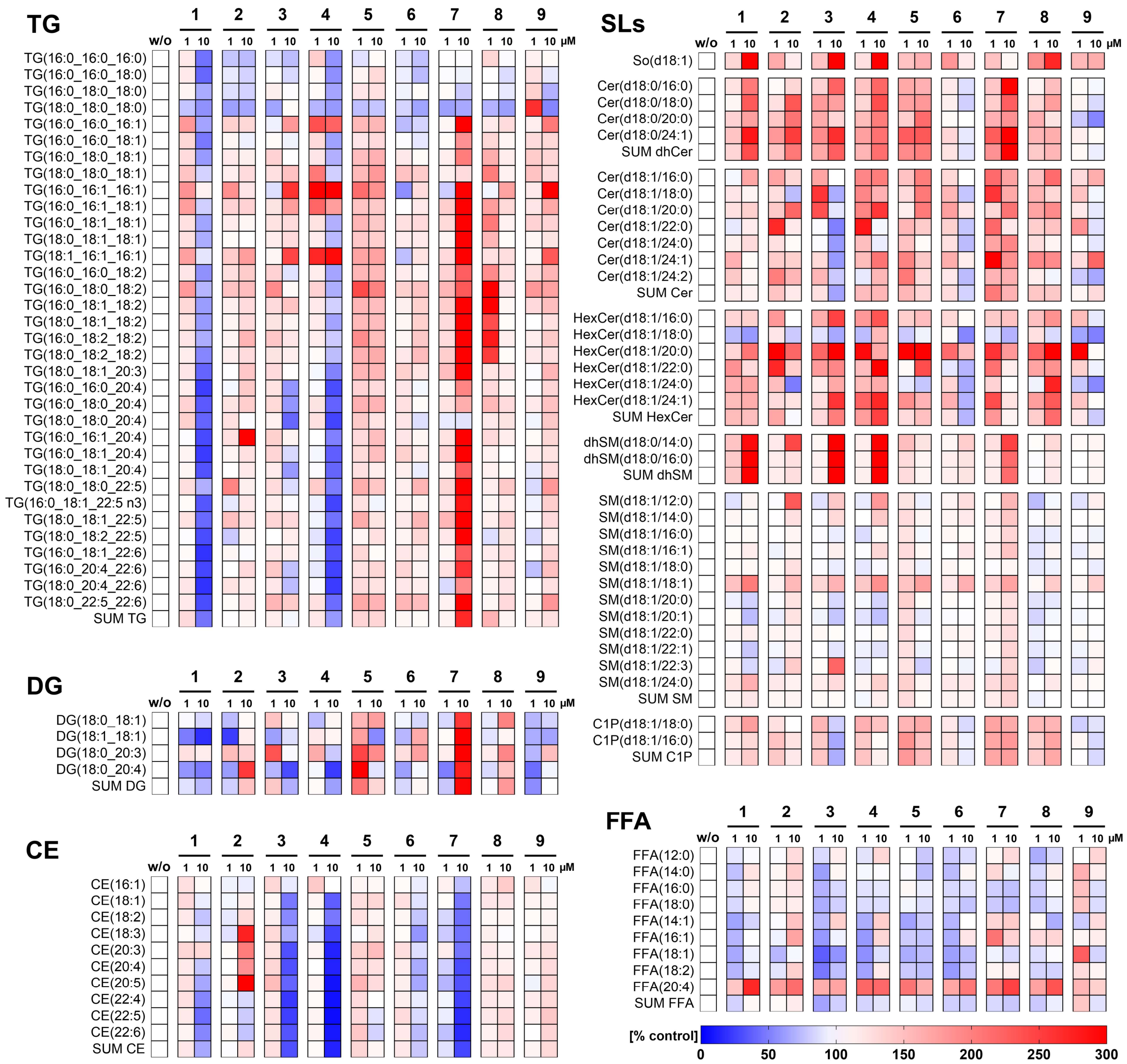

Figure S25. Effect of meroterpenoids on the composition of neutral lipids, sphingolipids (SLs), and FFAs in PBMCs. Cells were incubated with vehicle (DMSO, 0.1%) or meroterpenoids for 48 h. Heatmaps depict percentage changes in the concentrations of individual lipid species and the subclass sum ('SUM') relative to vehicle control. Data are given as mean from n = 3 independent experiments. Data for subclass sums are identical to Figure 10B.

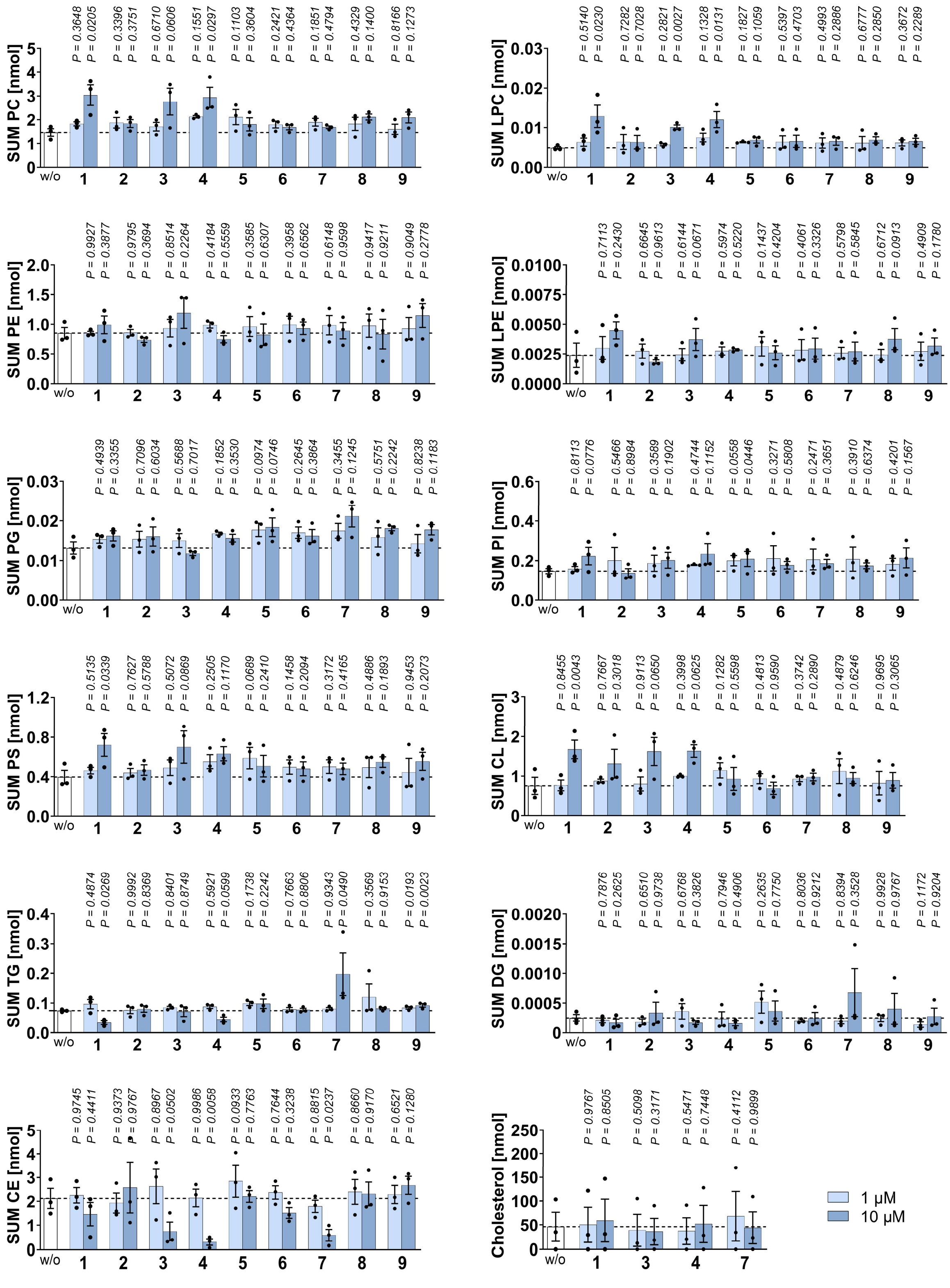

Figure S26. Absolute amounts of glycerophospholipid and neutral lipid subclasses in PBMCs treated with meroterpenoids. Cells were incubated with vehicle (DMSO, 0.1%) or meroterpenoids for 48 h. The amounts of lipid subclasses are shown in nmol per 1×10^6 cells. Data are identical to Figure 10B (all lipids) and Figure 7E (LPC, LPE). Mean + SEM and single data from n = 3 independent experiments. *P* values given vs. vehicle control; repeated measures one-way ANOVA of log data + Dunnett *post hoc* tests.

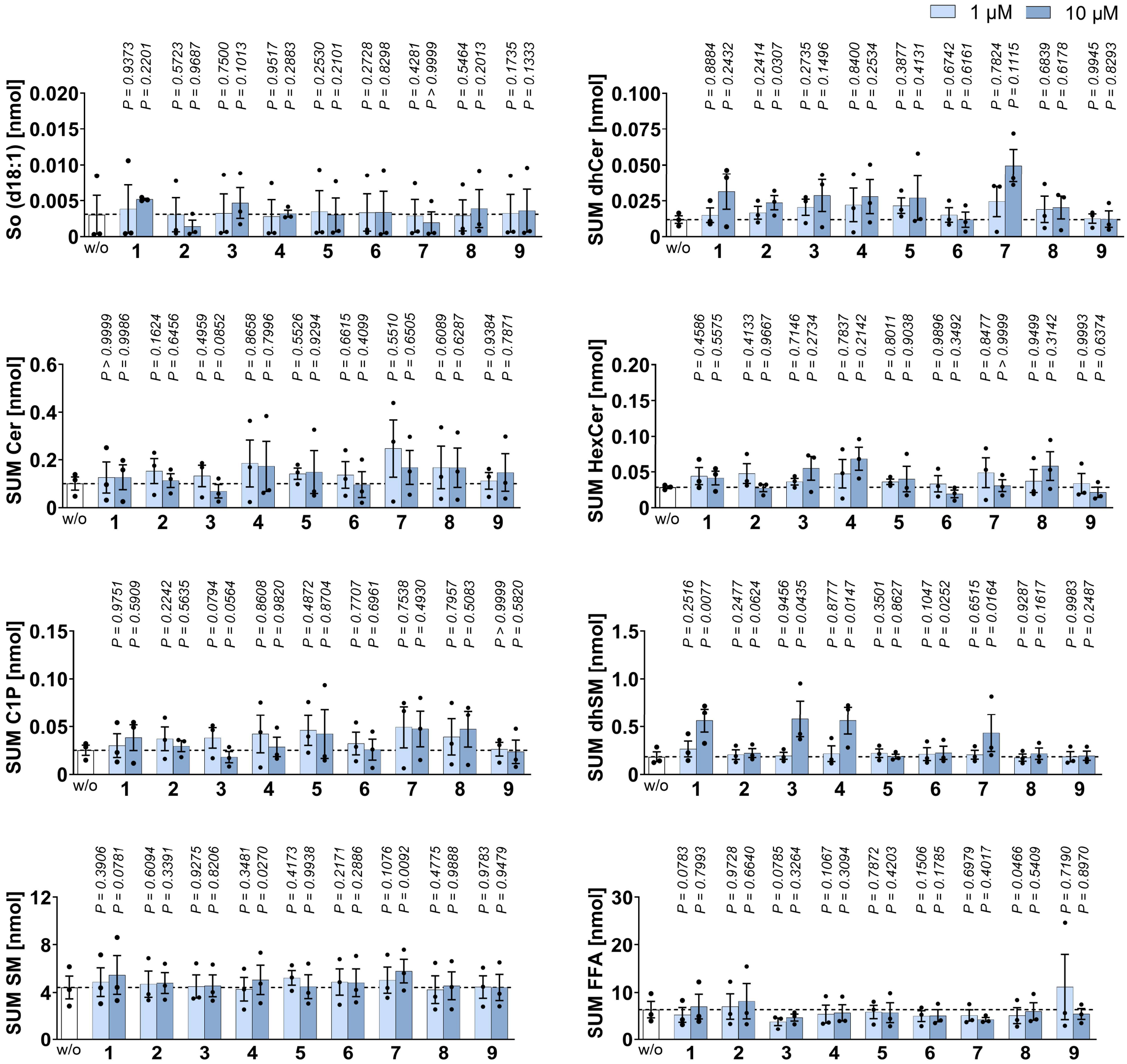

Figure S27. Absolute amounts of sphingolipid subclasses and FFAs in PBMCs treated with meroterpenoids. Cells were incubated with vehicle (DMSO, 0.1%) or meroterpenoids for 48 h. The amounts of lipid subclasses are shown in nmol per 1×10^{6} cells. Data are identical to Figure 10B (all lipids). Mean + SEM and single data from n = 3 independent experiments. *P* values given vs. vehicle control; repeated measures one-way ANOVA of log data + Dunnett *post hoc* tests.

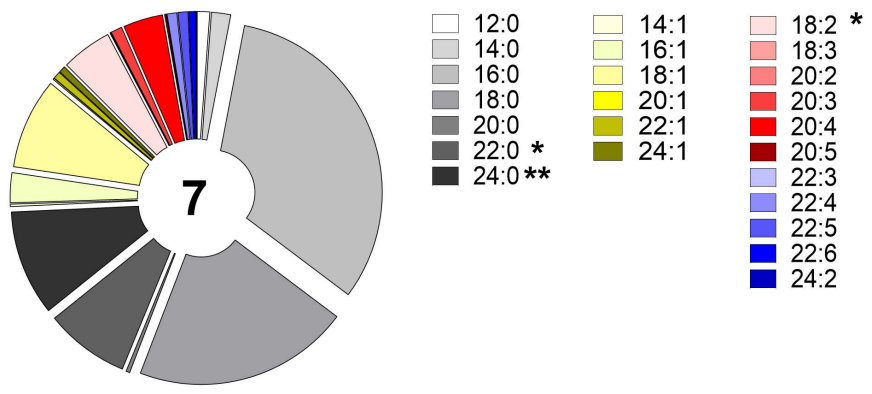

Figure S28. Proportion of fatty acids across all lipid species in PBMCs treated with **7**. Cells were incubated with vehicle (DMSO, 0.1%) or **7** (10 µM) for 48 h. The fatty acid composition of the vehicle control is shown in Figure 10C. Mean from n = 3 independent experiments. *P* values given vs. vehicle control; two-tailed paired Student *t* test.

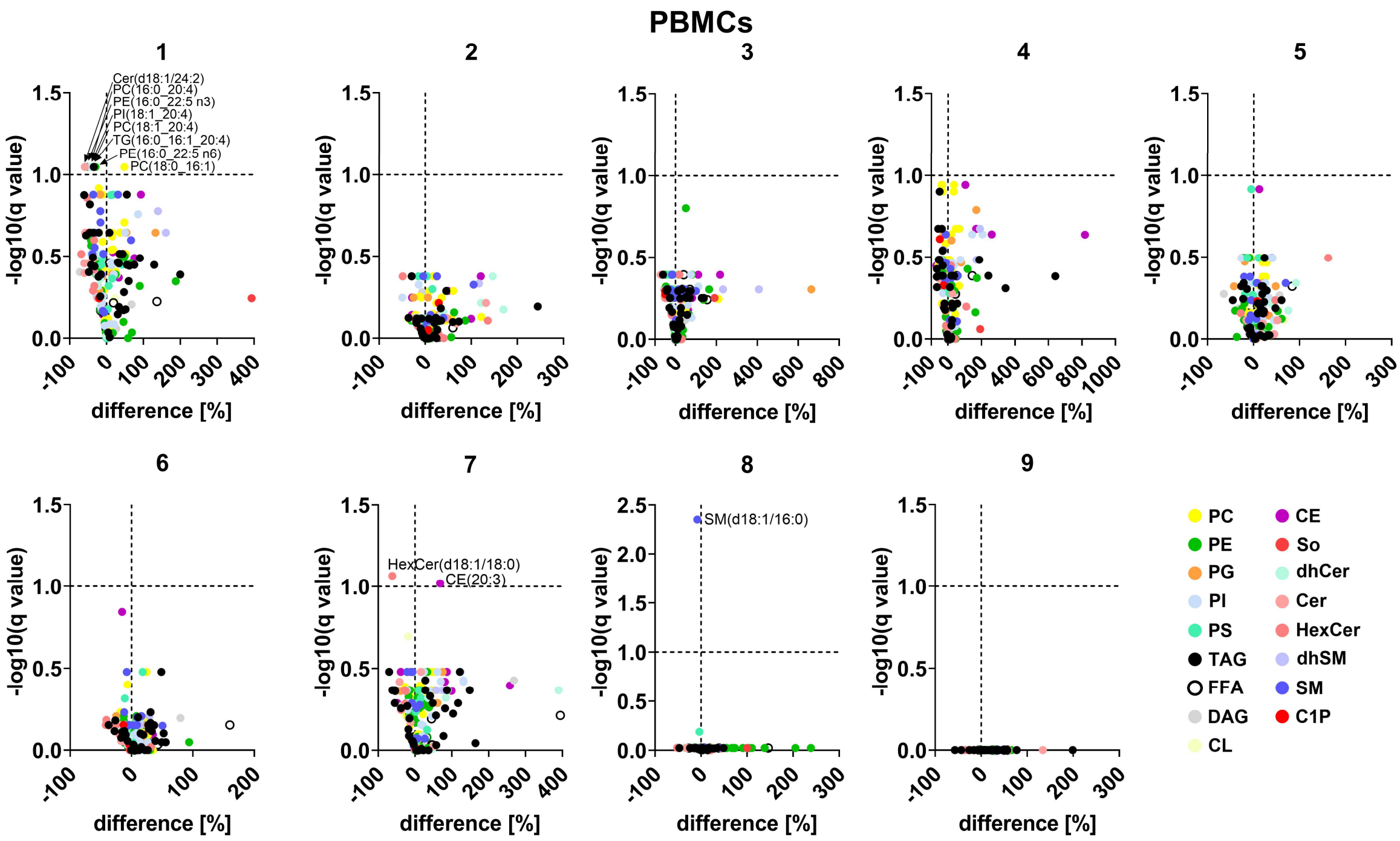

Figure S29. Effect of meroterpenoids on the relative lipid composition of PBMCs. Cells were incubated with vehicle (DMSO, 0.1%) or meroterpenoids (10 µM) for 48 h. Volcano plots depict the mean percentage difference relative to vehicle control and the negative $\log_{10}(q$ value) calculated vs. vehicle control from n = 3 independent experiments; two-tailed multiple paired Student *t* tests with correction for multiple comparisons (false discovery rate 10%).

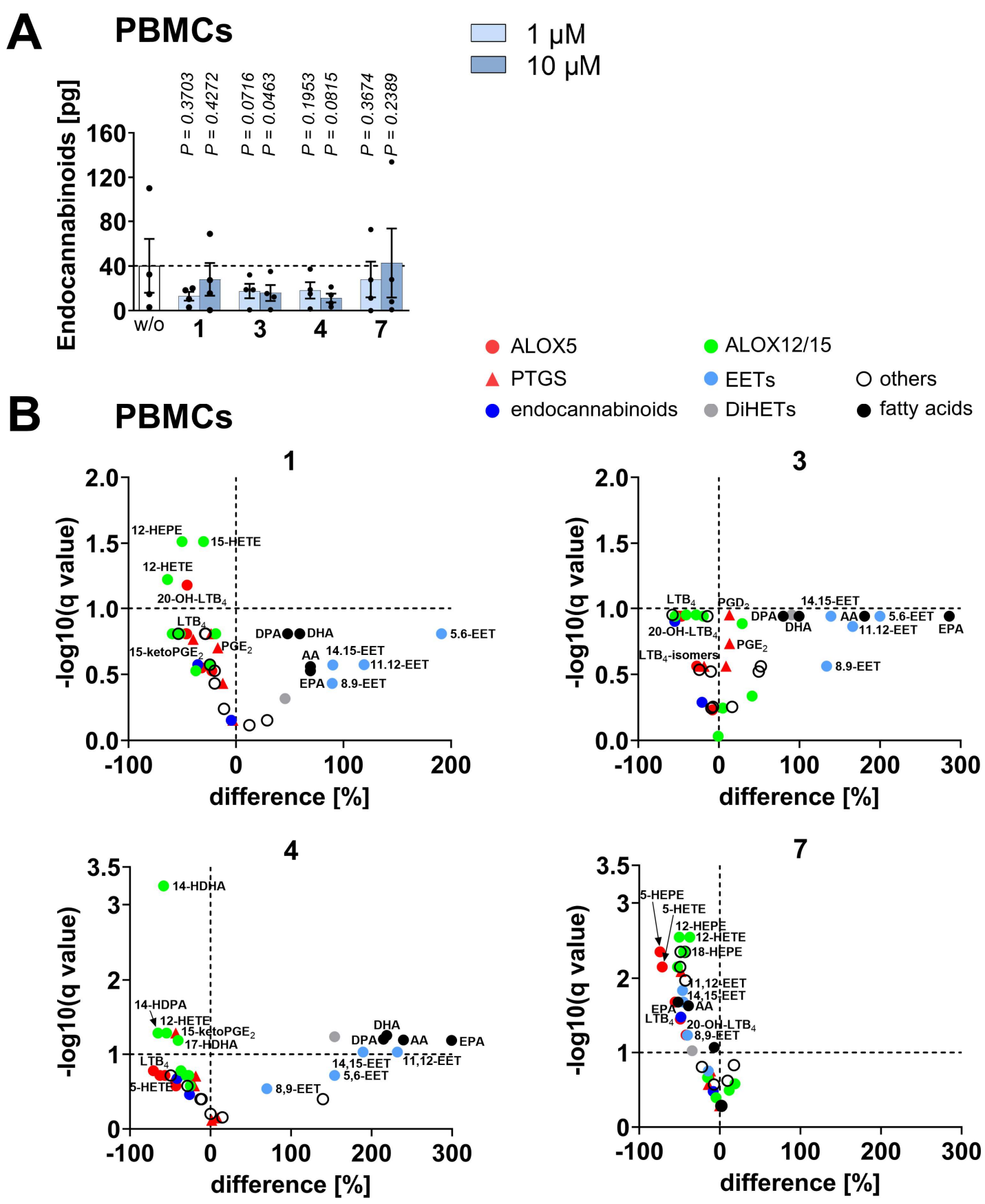

Figure S30. Changes in the capacity for lipid mediator biosynthesis following long-term exposure of PBMCs to **1**, **3**, **4** or **7**. Cells were pretreated (48 h) with vehicle (DMSO, 0.1%) or meroterpenoids (10 µM unless otherwise noted), washed, and activated with calcium ionophore A23187 (10 min). A) Endocannabinoid levels in pg per $1{\times}10^6$ PBMCs. B) Volcano plots depict the mean percentage difference relative to vehicle control and the negative $\log_{10}(q$ value) calculated vs. vehicle control; two-tailed multiple paired Student *t* tests with correction for multiple comparisons (false discovery rate 10%). Mean (B) or mean + SEM and single data (A) from n = 3-4 (B) or n = 4 (A) independent experiments. *P* values given vs. vehicle control; repeated measures one-way ANOVA of log data or mixed-effects model (REML) of log data + Dunnett *post hoc* tests (A).

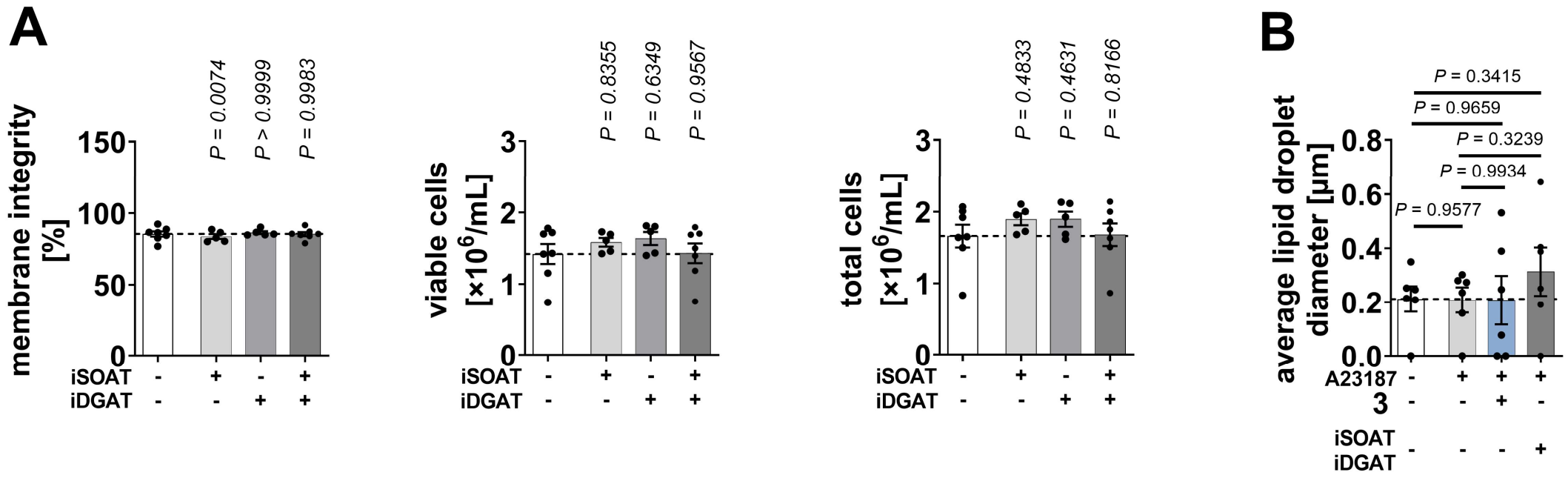

Figure S31. Combined SOAT and/or DGAT1/2 inhibition is compatible with PBMC survival and hardly affects lipid droplet diameter. A,B) PBMCs were treated for 48 h with vehicle (DMSO, 0.1%), **3** (10 µM), TMP-153 (iSOAT, 500 nM), A-922500 (10 nM) + PF-06424439 (20 nM) (iDGAT), or TMP-153 (100 nM) + A-922500 (10 nM) + PF-06424439 (20 nM) (iSOAT + iDGAT). A) Effect on membrane integrity and viable and total cell numbers. Mean + SEM and single data from n = 5-7 independent experiments. B) PBMCs were subsequently treated with calcium ionophore A23187 (5 min), and perilipin-2 was analyzed by immunofluorescence microscopy. Average lipid droplet diameter was calculated by dividing the total area of perilipin-2 staining by the number of individual perilipin-2 spots for each image. Two representative images (shown in Figure S33, Supporting Information) were analyzed per treatment group in n = 3 independent experiments. Data are presented as mean + SEM and single data points. *P* values given vs. vehicle control (A) or as indicated by bars (B); mixed-effects model (REML) + Dunnett *post hoc* tests (A) or two-tailed unpaired Student *t* test for pairwise comparisons (B).

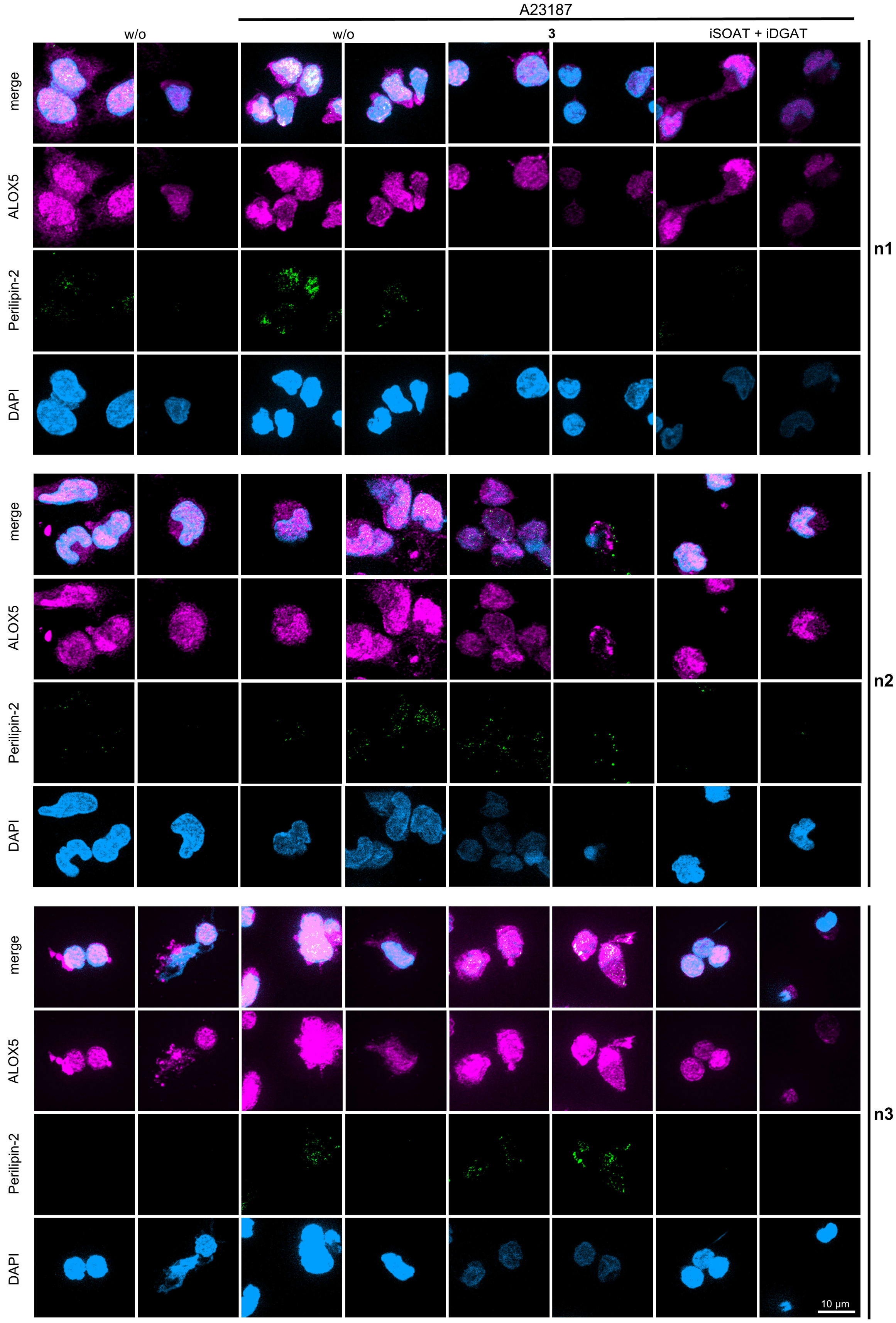

Figure S32. Effect of **3** and combined SOAT and DGAT inhibition on the abundance and subcellular distribution of perilipin-2 and ALOX5 in activated PBMCs. Cells were preincubated with vehicle (DMSO, 0.1%), **3** (10 µM), or TMP-153 (100 nM) + A-922500 (10 nM) + PF-06424439 (20 nM) (iSOAT + iDGAT) for 48 h. After medium exchange, cells were treated with calcium ionophore A23187 (5 min). Immunofluorescence images show perilipin-

2 (green, exposure time 35 ms), ALOX5 (violet, exposure time 200 ms), and nuclei (DAPI, light blue, exposure time 10 ms). Two representative images per treatment group are shown from n = 3 independent experiments.

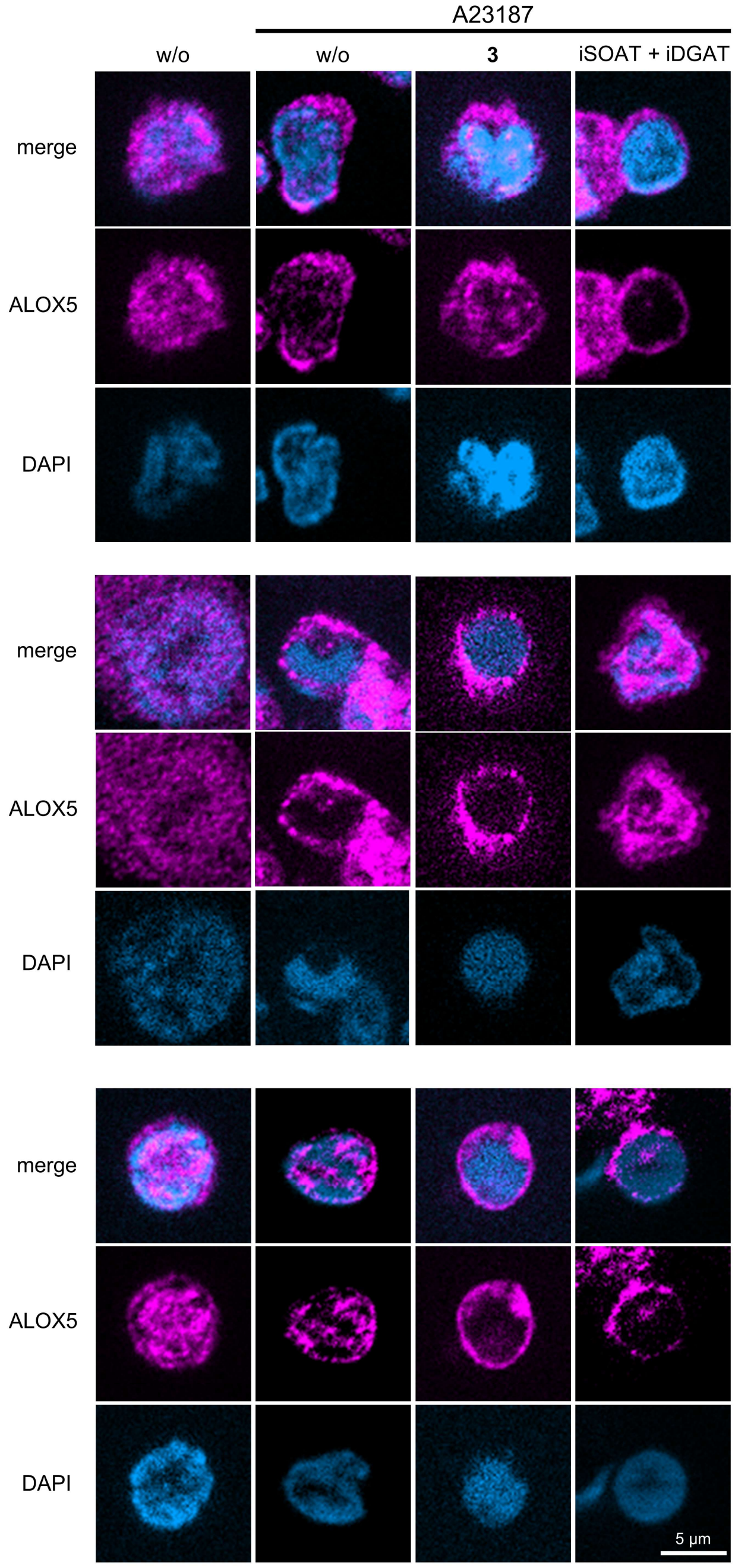

Figure S33. ALOX5 translocation in A23187-activated PBMCs is not affected by meroterpenoid **3** or dual SOAT/DGAT inhibition. Cells were preincubated with vehicle (DMSO, 0.1%), **3** (10 µM), or TMP-153 (100 nM) + A-922500 (10 nM) + PF-06424439 (20 nM) (iSOAT + iDGAT) for 48 h. After medium exchange, cells were treated with calcium ionophore A23187 (5 min). Immunofluorescence images show ALOX5 (violet, exposure time 200 ms) and nuclei (DAPI, light blue, exposure time 10 ms). Selected cells from single slices (Z = 1 µM) are shown from n = 3 independent experiments.

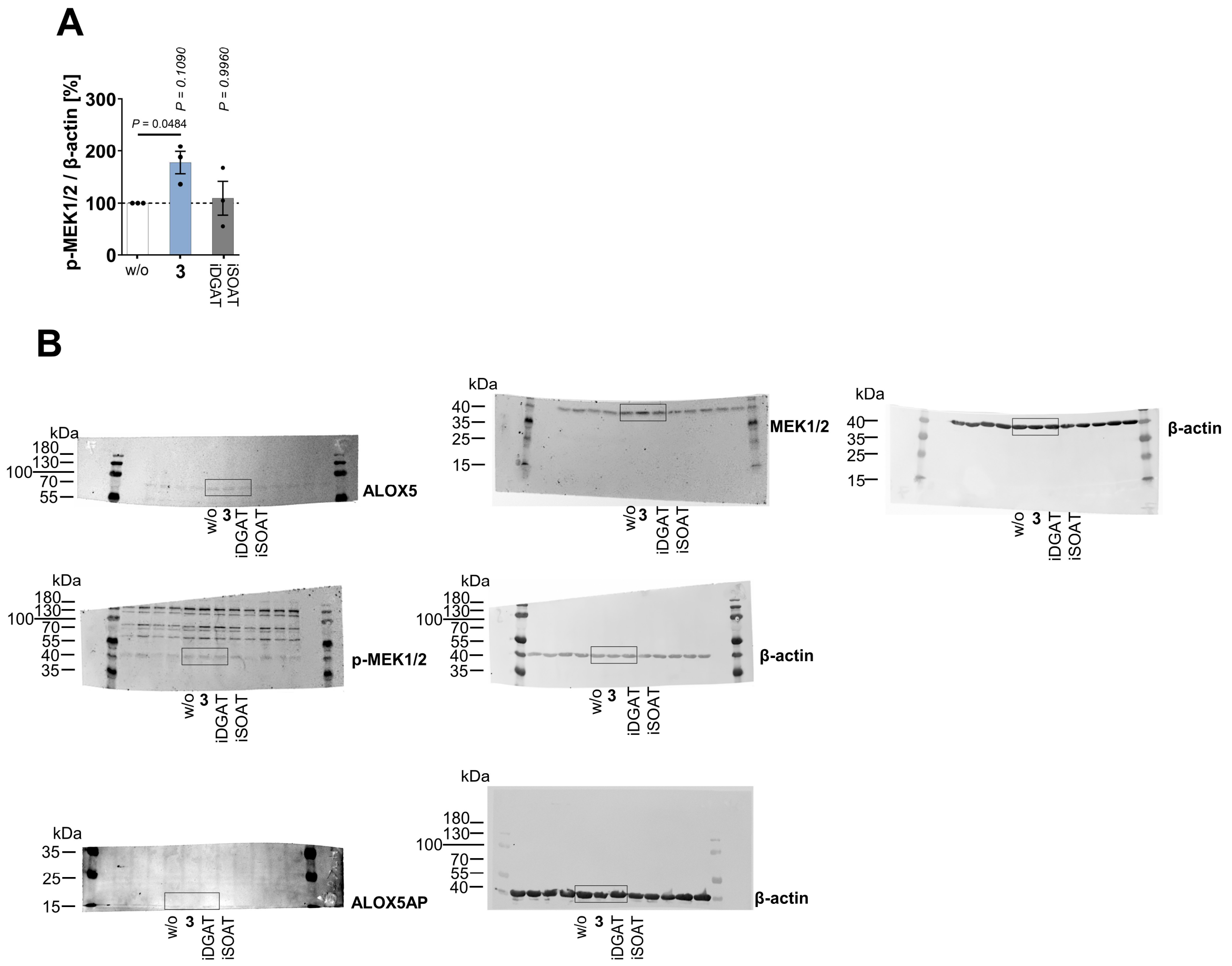

Figure S34. Effect of meroterpenoid **3** and combined SOAT/DGAT inhibition on the expression of key enzymes and regulatory factors in ALOX5 product biosynthesis. A,B) PBMCs were incubated with vehicle (DMSO, 0.1%), **3** (10 µM), or TMP-153 (100 nM) + A-922500 (10 nM) + PF-06424439 (20 nM) (iSOAT + iDGAT) for 48 h. A) Effect of **3** and iSOAT + iDGAT on the cellular p-MEK1/2 levels. Mean + SEM and single data from n = 3 independent experiments. *P* values given vs. vehicle control or as indicated by bars; repeated measures one-way ANOVA and two-tailed paired student *t* test for pairwise comparisons. B) Uncropped versions of the blots shown in Figure 8F.

Supplementary References

[1] L. Waltl, L. A. Wein, L. Bereuter, F. Su, H. Barta, L. L. Xuan, D. Holubek, Z. M. Eshkaftaki, K. Puskac, A. Siller, P. Schlenke, H. Schennach, E. M. Pferschy-Wenzig, H. Schött, S. Racedo, S. C. Koeberle, T. Magauer, A. Koeberle, *Theranostics* 2026, **16,** 5406, https://doi.org/10.7150/thno.127100.
